# Structural Snapshots on Stepwise Anionic Oxoborane Formation: Access to an Acyclic BO Ketone Analogue and Its Metathesis Chemistry with CO_2_ and CS_2_

**DOI:** 10.1021/acs.inorgchem.4c05354

**Published:** 2025-02-05

**Authors:** Marius Heitmann, Daniel Duvinage, Christopher Golz, Emanuel Hupf, Jens Beckmann, Malte Fischer

**Affiliations:** † Institut für Anorganische Chemie, Georg-August-Universität Göttingen, Tammannstraße 4, D-37077 Göttingen, Germany; ‡ Institut für Anorganische Chemie und Kristallographie, Universität Bremen, Leobener Street 7, D-28359 Bremen, Germany; § Institut für Organische und Biomolekulare Chemie, Georg-August-Universität Göttingen, Tammannstraße 2, D-37077 Göttingen, Germany

## Abstract

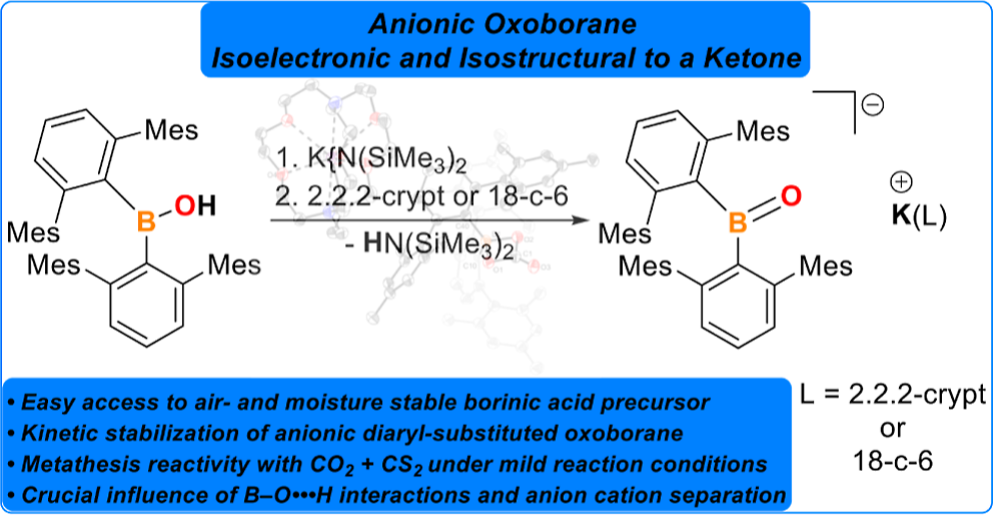

In this work, we disclose the synthesis and characterization of non-acid/base-stabilized anionic oxoboranes [^Mes^Ter_2_BO][K(L)] (^Mes^Ter = –C_6_H_3_-2,6-(2,4,6-Me_3_-C_6_H_2_)_2_, L = [2.2.2]-cryptand or 18-crown-6), which are isoelectronic and isostructural with aryl-substituted ketones. The stepwise synthetic formation of these ion-separated oxoboranes is demonstrated on the one hand by the treatment of the parent borinic acid ^Mes^Ter_2_BOH with *N*-heterocyclic carbenes (NHCs) to give [^Mes^Ter_2_BO][HNHC] derivatives, and on the other hand by a deprotonation-sequestration sequence. Bearing polarized boron–oxygen moieties, their inherent reactivity toward both carbon disulfide and carbon dioxide reveals a unique π-bond metathesis reactivity to yield [(^Mes^Ter)_2_B-μ-E_2_C=E][K(L)] (E = O, S) derivatives.

## Abstract 2

Easy access to air- and moisture stable borinic acid precursor. Kinetic stabilization of anionic diaryl-substituted oxoborane. Metathesis reactivity with CO_2_ + CS_2_ under mild reaction conditions. Crucial influence of B–O···H interations and anion cation separation.

## Introduction

Ketones such as the parent diaryl ketone benzophenone, represent a vital class of organic compounds widely encountered in synthetic contexts due to their reactive carbonyl functional group (R_2_C=O). Their significance extends across various domains, including medicinal chemistry and biochemical processes.

The field of main group compounds with multiple bonds has expanded rapidly since the formulation of the “double bond rule”.^1^ However, isolating “bottleable” compounds with double bond character between p-block elements and oxygen remains difficult to achieve.

**Scheme 1:**
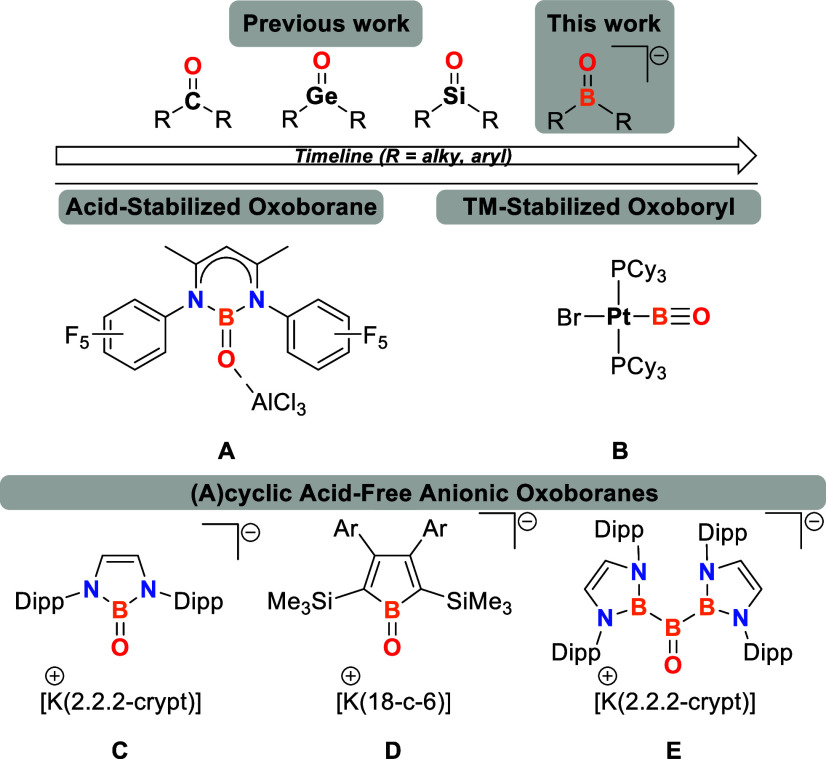
Ketones and Heavier Analogues (Top Row, previous and this work) and Examples of the First Lewis Acid-Stabilized Oxoborane A, the Transition Metal Stabilized Oxoboryl B, (a) cyclic Acid-Free Anionic Oxoborane Derivatives C–E

Remarkable progress has been made in recent years in isolating isoelectronic carbonyl derivatives of the heavier Group 14 elements without electronic stabilization by π-donating substituents. Since the isolation of the first heavier ketones, namely a monomeric terminal germanone of type R_2_Ge=O by Tamao et al. and a terminal silanone of type R_2_Si=O by Iwamoto et al. through kinetic stabilization,^2^ thus fulfilling Kipping’s dream,^3^ the foundations have been laid for striking advancements in the field (Scheme 1, top).^4^ Recent studies compellingly demonstrate that several unusual terminal group 15 oxides can now also be successfully synthesized.^ ^4d,4e,4f,4g^−4h^

Given the indispensable role of ketones and their kinetically stabilized heavier group 14 analogues in the molecular landscape, access to isoelectronic species, such as anionic oxoboranes of the general type [R_2_B=O]^−^,^5^ and investigating their unique properties hold substantial potential for fundamental understanding of cross-relationships and their translation into practical applications. However, this pursuit is coupled with various synthetic challenges and is further related to the high polarity of the boron–oxygen fragment, the Lewis acidic nature of boron, and its propensity for oligo- and polymerization.^1^ Consequently, monomeric [R_*n*_BO]^*m*^ (*n* = 1, *m* = 0; *n* = 2, *m* = −1) species are typically observed only in the gas phase, in low-temperature matrices, or indirectly through trapping products in subsequent reactions.^1,6^

Cowley and co-workers demonstrated in 2005 how Lewis-acid stabilization of the Lewis basic moiety, or vice versa, can be used to isolate monomeric oxoboranes, a strategy subsequently adopted frequently for the isolation of ’tamed’ oxoboranes (*cf*. **A** in Scheme 1).^7^ Similarly, Braunschweig, Yamashita, and Miyada employed the strategy of stabilizing multiple bonds between boron and oxygen in the vicinity of transition metals (*cf*. **B** in Scheme 1).^8^ Recent breakthroughs in isolating compounds containing boron–oxygen multiple bonds include the successful isolation of acid/base-free cyclic or acyclic anionic, neutral, and parent oxoboranes.^9^ However, non-Lewis-pair stabilized oxoboranes have so far only been isolated with unsaturated cyclic frameworks, aided by resonance forms, and the first and only acyclic example so far is supported by diboryl substitution (*cf*. the carbamide/urea derivative **C**, the heterocyclopentadiene derivative **D**, and the bis(boryl)-substituted derivative **E**).^9^

Accordingly, the isolation of an acyclic anionic oxoborane, which is isoelectronic and structurally related to a ketone, still remains elusive. The challenges in isolating such a species are further highlighted by recent work, which describes the formation of carbanions instead of terminal anionic oxoboranes upon deprotonation of a borinic acid derivative with pendant aryl functionalities and subsequent sequestration of the cation.^10^

In this study, we bridge this gap and demonstrate the stepwise synthesis, characterization, and unique reactivity of an anionic oxoborane bearing two sterically demanding terphenyl substituents, thereby establishing its structural similarity to classic ketones such as benzophenone.

## Results and Discussion

The recently reported bis(*m*-terphenyl)boron fluoride (^Mes^Ter)_2_BF (**1**) (^Mes^Ter = –C_6_H_3_-2,6-(2,4,6-Me_3_-C_6_H_2_)_2_) was identified as a suitable candidate to access a monomeric anionic oxoborane with two flanking aryl substituents.^11^ This consideration is based on the sterically demanding nature of the σ-donating *m*-terphenyl ligand class,^12^ which aims to prevent the usually observed oligo- and polymerization processes and aggregations *via* hydrogen bonding of the reactive boron–oxygen moiety of the targeted anionic oxoborane species.^13^

**Scheme 2:**
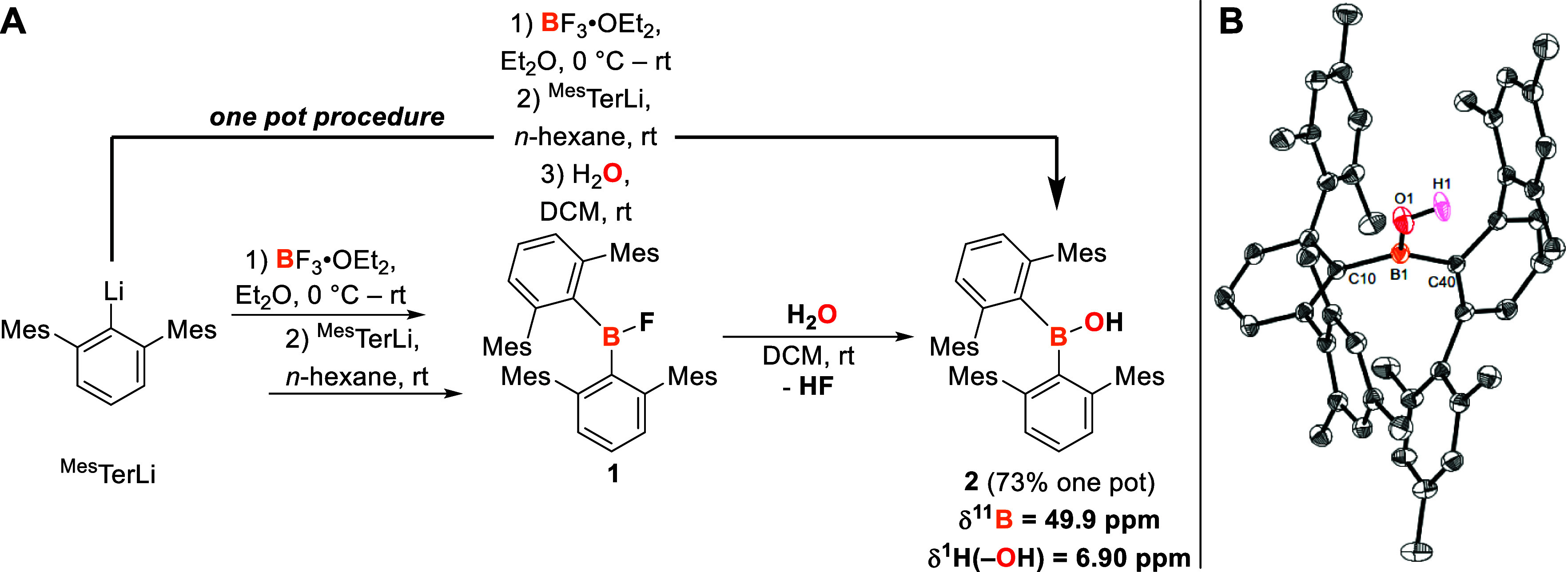
(A) Synthesis of the Borinic Acid (^Mes^Ter)_2_BOH (2) (NMR Chemical Shift Given in C_6_D_6_ as Solvent and at 298 K); (B): Molecular Structure of ^Mes^Ter_2_BOH (2) in the Crystal after Hirshfeld Atom Refinement (Monoclinic Modification) Anisotropic displacement parameters are drawn at the 50% probability level. Only the freely and anisotropically refined H1 atom is shown, while all other hydrogen atoms, although also refined freely and anisotropically, are omitted for clarity. Selected bond lengths (Å) and angles (deg) of both polymorphs: Monoclinic polymorph: B1–O1 1.3528(18), B1–C10 1.600(2), B1–C40 1.598(2), O1–H1 0.978(19), C10–B1–O1 114.10(12), C40–B1–O1 120.28(12), C10–B1–C40 124.94(12); orthorhombic polymorph: B1–O1 1.3558(7), B1–C10 1.6120(4), O1–H1 0.894(13), C10–B1–O1 113.67(2), C10–B1–C10′ 132.65(5).

By treating **1** with water in dichloromethane, the air- and water-stable borinic acid (^Mes^Ter)_2_BOH (**2**) can be accessed as a colorless crystalline solid through recrystallization from DCM/hexane or purification by column chromatography (Scheme 2, **A**).^14,15^ Notably, **2** can also be synthesized in a one-pot procedure starting directly from ^Mes^TerLi and BF_3_·OEt_2_,^14,16^ yielding 73% overall, thereby enhancing its synthetic accessibility.

Borinic acid **2** was characterized by multinuclear NMR spectroscopy, elemental combustion analysis (EA), high-resolution mass spectrometry (HRMS), infrared (IR) spectroscopy and single crystal X-ray diffraction (SC-XRD) (Scheme 2, **B**).^14^

Notably, **2** crystallized in the form of two polymorphs (monoclinic *P*2_1_/*n* and orthorhombic *Fdd*2) dependent on the applied crystallization conditions.^14,17^

**Scheme 3:**
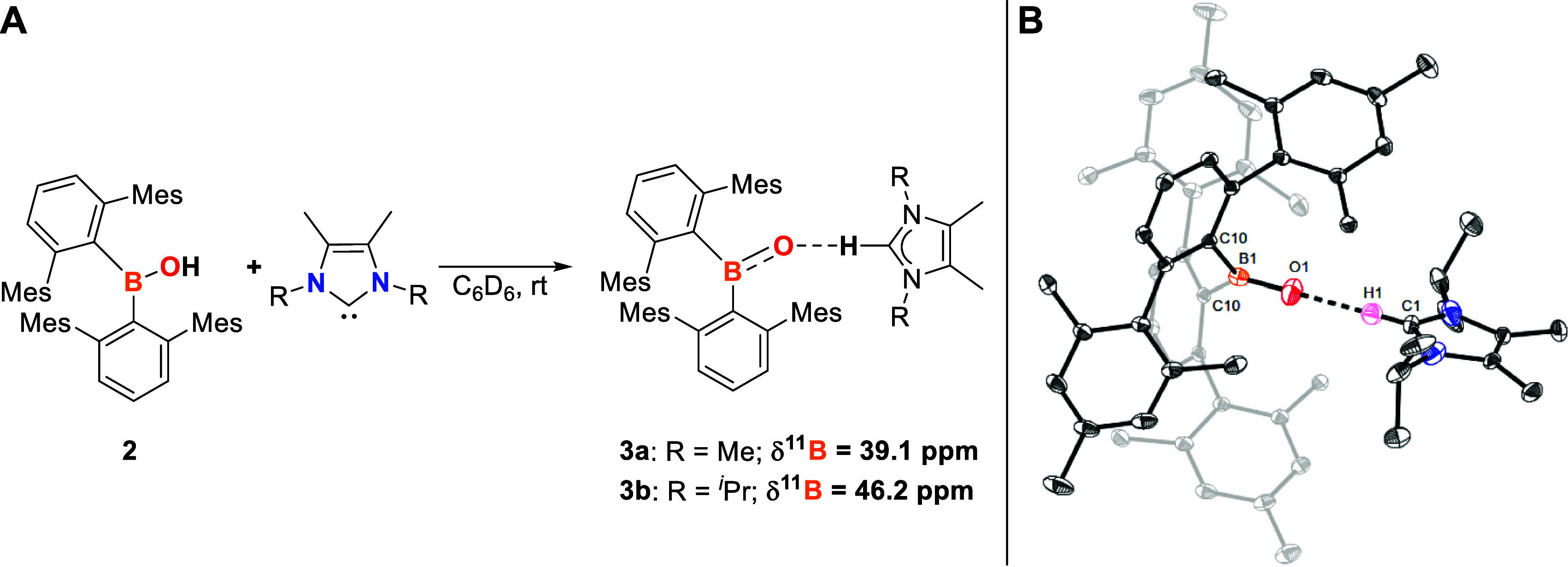
(A) Reactivity of 2 towards the NHCs IMe_4_ and I^*i*^Pr_2_Me_2_ to Yield **3a** and **3b** (NMR Chemical Shift Given in C_6_D_6_ as Solvent and at 298 K); (B) Molecular Structure of [(^Mes^Ter)_2_BO][HI^*i*^Pr_2_Me_2_] (**3b**) in the Crystal after Hirshfeld Atom Refinement Anisotropic displacement parameters are drawn at the 50% probability level. Only the freely and anisotropically refined H1 atom is shown, while all other hydrogen atoms, although also refined freely and anisotropically, are omitted for clarity. Selected bond lengths (Å) and angles (deg): B1–O1 1.2961(11), B1–C10 1.6528(7), O1···H1 1.7973(92), O1–B1–C10 118.30(3), C10–B1–C10′ 123.40(7), B1–O1–H1 180, C1–H1–O1 180.

With **2** in hand, it was reacted with the *N*-heterocyclic carbene (NHC) 1,3,4,5-tetramethyl-2-imidazole-2-ylidene (IMe_4_) to examine whether the steric bulk of the flanking *m*-terphenyl substituents is sufficient to prevent coordination of the generally Lewis basic IMe_4_ to the Lewis acidic boron atom and following the report by Cui et al., in which IMe_4_ was used to abstract a BOH hydrogen atom.^7c,18^ The reaction occurred immediately and led to a slight, yet noticeable color change from colorless to pale yellow. Multinuclear NMR spectroscopy revealed the formation of [(^Mes^Ter)_2_BO][HIMe_4_] (**3a**) (Scheme 3, A), evident from its ^11^B NMR chemical shift at δ = 39.1 ppm. This represents a 10.8 ppm shift toward higher field compared to the parent borinic acid (Scheme 2, **A**), consistent with increased electron density at boron. The formation of the corresponding imidazolium ion was characterized by the ^1^H NMR chemical shift of the C–H moiety, appearing at δ = 11.66 pm as a broad singlet.^14^

Crystals of **3a** suitable for SC-XRD were obtained from a saturated diethyl ether solution at −30 °C, disclosing an ion pair consisting of an oxoborane anion and imidazolium cation (Figure S20).^14^ The B1–O1 bond length of **3a** (1.293(2) Å) is approximately 0.6 Å shorter than that found in **2** [1.3528(18) Å], thus suggesting oxygen-to-boron π-donation and increased B=O double bond character.^14^ Consistently, the adjacent B–C bonds are elongated when compared to **2** [mean 1.64 Å (**3a**) vs mean 1.60 Å (**2**)]. The short B–O bond distance is in the middle of reported compounds with B–O double bond character [1.273(8)–1.329(6) Å].^7−9^ As in **2**, the boron atom in **3a** is in a trigonal planar coordination environment (Σ Spherical Angle B1 = 359.8°). The cation and anion are interacting with each other through a C–H···O hydrogen bond. The precise and accurate position of the hydrogen bonds was determined by Hirshfeld atom refinement (HAR).^19^ The observed O1···H1 separation of 1.832(26) Å and B1–O1–H1 and C1–H1–O1 bond angles of 159 and 153°, respectively, account for medium strength hydrogen bonding.^14^

By using the sterically more demanding 1,3-di*iso*propyl-4,5-dimethyl-2-imidazole-2-ylidene (I^*i*^Pr_2_Me_2_) in the reaction with **2**, [(^Mes^Ter)_2_BO][HI^*i*^Pr_2_Me_2_] (**3b**) is obtained (Scheme 3, A).

The molecular structure reveals that in comparison to **3a**, the C–H···O hydrogen bonding interaction is slightly stronger [1.7973(92) Å] and the B1–O1···H1–C1 moiety is in a linear arrangement (O1–B1–H4 and C4–H4–O1 both 180°), whereas the B1–O1 bond length of 1.2961(11) Å is in the same range (Scheme 3, B). These values are in good agreement to the anionic oxoborane imidazolium salt [{HC(CMe)_2_(NDipp)_2_}BO][HI^*i*^Pr_2_Me_2_].^7c^

**Scheme 4:**
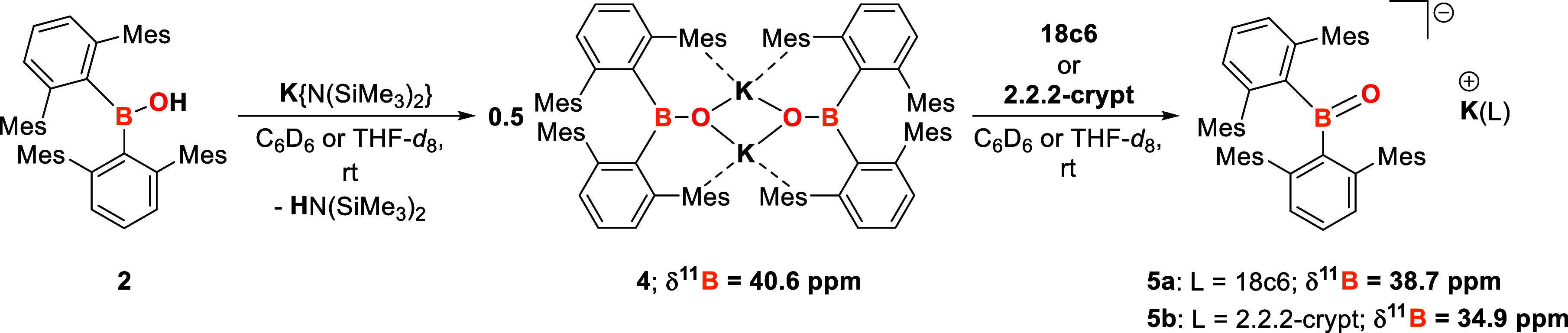
Reaction of 2 with K{N(SiMe_3_)_2_} to Yield [(^Mes^Ter)_2_BOK]_2_ (4) and Subsequent Sequestration of Potassium by 18c6 and 2.2.2-crypt to Give [(^Mes^Ter)_2_BO](K(18c6)] (**5a**) and [(^Mes^Ter)_2_BO][K(2.2.2-Crypt)] (**5b**), Respectively (NMR Chemical Shift Given in C_6_D_6_ as Solvent and at 298 K)

Considering our goal of isolating an anionic oxoborane, which is isoelectronic and structurally related to a ketone, we utilized the deprotonation of **2** with potassium bis(trimethylsilyl)amide (K{N(SiMe_3_)_2_}) and subsequent sequestration of the potassium cation by 18-crown-6 (18c6) and [2.2.2]-cryptand (2.2.2-crypt) (Scheme 4).

**Figure 1:**
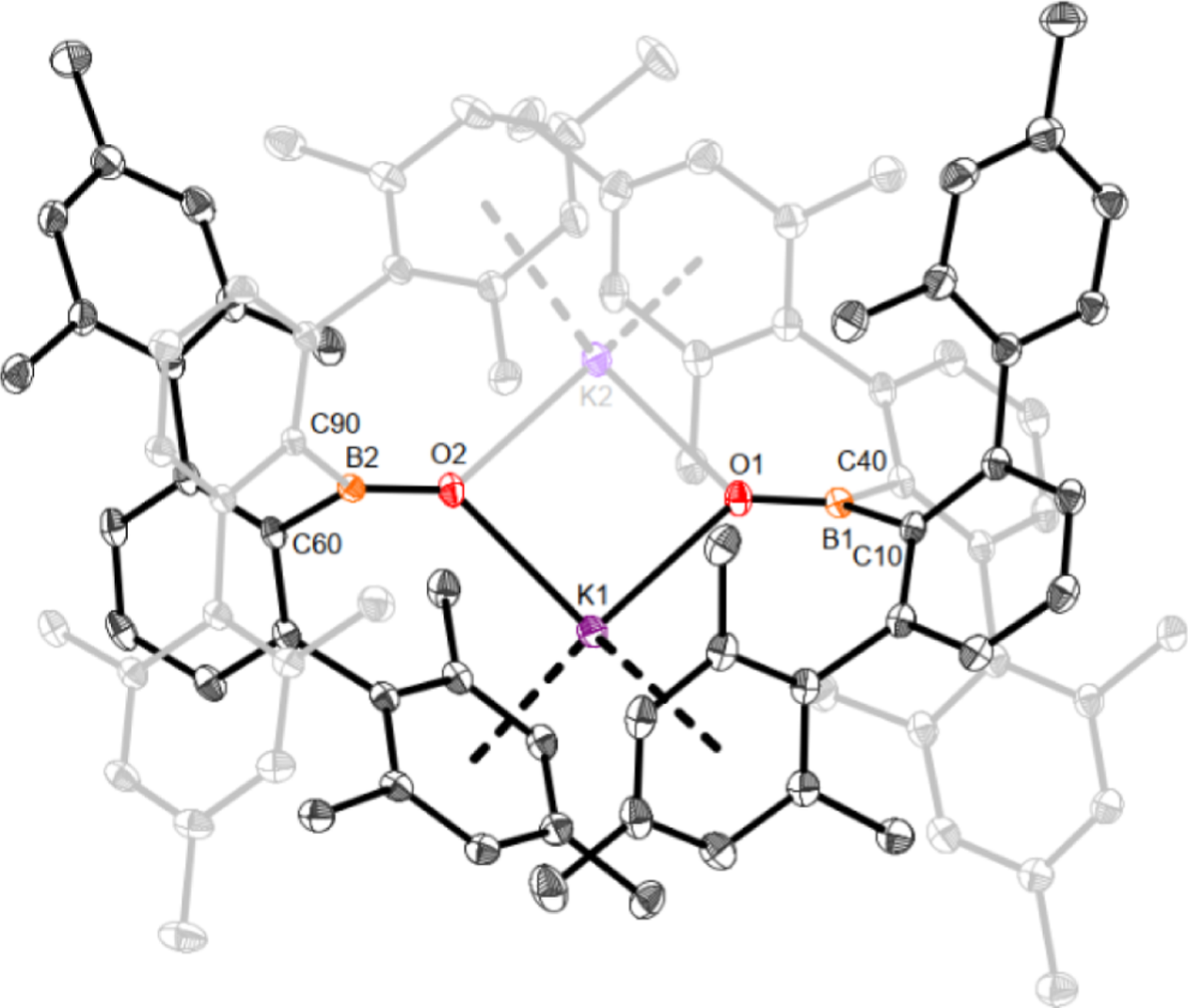
Molecular structure of [^Mes^Ter_2_BOK]_2_ (4) in the crystal. Anisotropic displacement parameters are drawn at the 50% probability level (hydrogen atoms and lattice solvent have been omitted for clarity). Selected bond lengths (Å) and angles (deg): B1–O1 1.3054(18), B1–C10 1.642(2), B1–C40 1.645(2), B2–O2 1.3058(17), B2–C60 1.647(2), B2–C90 1.649(2), O1–K1 2.5856(10), O1–K2 2.5838(11), O2–K1 2.5890(11), O2–K2 2.5740(10), O1–B1–C10 122.91(12), O1–B1–C40 123.01(12), C10–B1–C40 114.07(11), O2–B2–C60 122.06(12), O2–B2–C90 123.14(12), C60–B2–C90 114.80(11), O1–K1–O2 92.66(3), O1–K2–O2 93.05(3), K1–O1–K2 87.00(3), K1–O2–K2 87.13(3).

The reaction of **2** with K{N(SiMe_3_)_2_} in either THF or benzene proceeds immediately at room temperature, cleanly yielding the corresponding potassium oxoborane [(^Mes^Ter)_2_BOK]_2_ (**4**). **4** was characterized by multinuclear NMR spectroscopy, EA, HRMS, and SC-XRD (Scheme 4 and Figure 1).

The molecular structure of **4** in the solid state reveals a dimer with two formally anionic [(^Mes^TerBO)]^−^ units bridged by flanking potassium–arene contacts (distance between the potassium counterions and the aryl centroids 2.88–3.12 Å). The oxygen–potassium distances of mean 2.58 Å match the single bond covalent radii of the respective atoms (2.59 Å).^20^ Both boron atoms adopt, as expected, trigonal planar coordination environments and the B1–O1 and B2–O2 bond lengths of 1.3054(18) and 1.3058(17) Å are longer than those observed for **3a** and **3b** and reflect the stronger ionic interaction of the anionic oxoborane moiety and potassium in **4** when compared to the hydrogen bonding interactions in **3a** and **3b**.^1^

The solution NMR data for compound **4** in C_6_D_6_ are consistent with the molecular structure observed in the solid state. This is attributed to interactions between the potassium cations and one flanking mesityl group of each ^Mes^Ter ligand. As a result, six distinct signals for the mesityl methyl groups are observed in the corresponding ^1^H NMR spectrum at δ = 1.36, 2.00, 2.02, 2.04, 2.27, and 2.28 ppm.^14^ In line with the reduced symmetry compared to the starting material **2**, more than three signals are observed for the aromatic hydrogen atoms. The ^11^B{^1^H} NMR signal is shifted to higher field relative to **2**, appearing at δ = 40.6 ppm. In comparison to other potassium salts derived from borinic acids with *N*-heterocyclic backbones, this signal is shifted to lower field by over 17 ppm, indicating a less electron-rich boron center due to the absence of pπ–pπ interactions.^9b,15^ Interestingly, NMR data collected in THF-*d*_8_ shows signals indicative of higher symmetry and accordingly, one signal each for the *ortho* and *para* methyl group of the ^Mes^Ter functionalities, which we attribute to coordination of THF to the potassium atoms, competitively preventing potassium arene interactions.^14^ Notably, growing crystals from THF solutions always led to the same molecular structure shown in Figure 1.

For the sequestration of the potassium ion, both 18c6 and 2.2.2-crypt were employed in reactions with **4**. The successful formation of the separated ion pairs [(^Mes^Ter)_2_BO][K(18c6)] (**5a**) and [(^Mes^Ter)_2_BO][K(2.2.2-crypt)] (**5b**) is evident by the formation of two layers, in the case of **5b**, when apolar solvents such as benzene are used. This is further supported by the clean detection of the respective signals of the cationic and anionic moieties in the positive and negative modes of electrospray ionization mass spectrometry (ESI-MS).

In accordance to increased oxygen-to-boron π-donation upon generation of these free anionic oxoboranes, ^11^B NMR signals at δ = 38.7 (C_6_D_6_)/36.0 (THF-*d*_8_) (**5a**) and 34.9 ppm (**5b**, THF-*d*_8_) are detected.^14^

Infrared (IR) spectroscopy is a widely utilized tool for assessing the strength of the B–O bond and its multiple-bond character.^7,9^ In previously reported three-coordinate oxoboranes–neutral, anionic, and/or stabilized–the B–O stretching vibrations span a range from 1462 cm^–1^, observed in a bis(boryl)-substituted anionic oxoborane,^9g^ to 1667 cm^–1^ in a neutral *N*-heterocyclic oxoborane.^9d^ Computational studies estimate the free anion’s B–O stretch of **5** at 1482 cm^–1^, placing it at the lower end of this range. For the compounds reported here, containing B–O moieties, multiple signals appear within this region.^14^ However, due to the complexity of the spectra, we refrain from providing detailed interpretations at this stage, as a definitive assignment remains challenging. Unfortunately, despite our best efforts, no crystals of **5a** and **5b** suitable for SC-XRD could be obtained, as all attempts resulted only in microcrystalline material or oily residues.^14^

**Figure 2:**
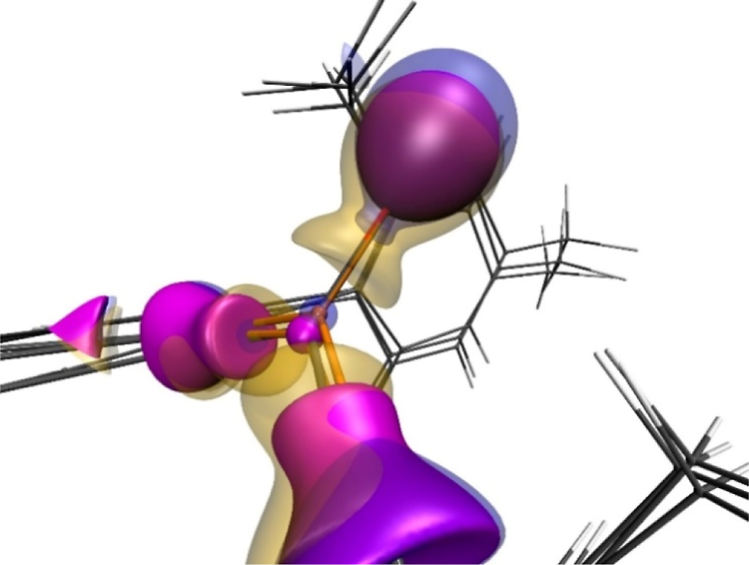
Laplacian of the electron density of (^Mes^Ter)_2_CO (transparent yellow), [(^Mes^Ter)_2_BO]^−^ (transparent blue), and (^Mes^Ter)_2_BF (solid magenta) at iso-surface values of −0.3 e bohr^–5^.

We conducted a computational analysis of the B–O interaction within the anion [(^Mes^Ter)_2_BO]^−^. Natural bond orbital (NBO)^21^ analysis revealed two bonding orbitals with a Wiberg bond index (WBI) of 1.35 and natural population analysis (NPA) charges of +0.96 (B) and −1.02 (O). These values closely resemble those of Aldridge’s anionic oxoborane (WBI 1.40, *q*_NPA_(B/O) +0.99/–1.03),^9b^ supporting the description of a B=O double bond. The π-bond character is also evident in the HOMO – 1, akin to a C=O bond in the related ketone (^Mes^Ter)_2_CO (Figure S77). However, the NPA charge separation of the ketone is smaller compared to the B–O interaction, indicating greater ionicity in the anionic oxoborane (Table S8). This is further corroborated by an electron density analysis using the atoms in molecules (AIM) approach.^22^ The Laplacian of the electron density at the B–O bond critical point [∇^2^ρ(*r*) = 27.2 e·Å^–5^] is significantly higher than that of [^Mes^Ter)_2_CO (∇^2^ρ(*r*) = −5.3 e·Å^–5^], and is comparable to the B–F bond in (^Mes^Ter)_2_BF [∇^2^ρ(*r*) = 28.5 e·Å^–5^].^11^ Additionally, other key AIM-derived parameters at the bond critical point, including electron density and energy over electron density ratios, reveal that the B–O bonding situation lies between the more covalent C–O bond and the predominantly ionic B–F bond (Tables S6 and S7). This trend is further reflected in the delocalization index, which decreases from δ = 1.35 in (^Mes^Ter)_2_CO to δ = 0.64 in [(^Mes^Ter)_2_BO]^−^. The respective Laplacians illustrating this behaviour are shown in Figure 2.

To further elucidate the bonding characteristics and quantify the degrees of covalency and ionicity, we performed an interacting quantum atoms (IQA) analysis.^23^ Due to the computational costs, the IQA analysis was carried out on the smaller phenyl-substituted analogues Ph_2_CO, [Ph_2_BO]^−^, and Ph_2_BF. In IQA, the total interaction energy (*E*_int_) between two atomic basins is expressed as the sum of the total Coulomb potential energy (*E*_C_), representing the ionic contribution, and the exchange–correlation energy (*E*_XC_), reflecting the covalent component. The B–O interaction in [Ph_2_BO]^−^ exhibits the highest total interaction energy of *E*_int_ = −1017 kcal mol^–1^ with predominant ionic contribution (*E*_C_ = −898 kcal mol^–1^, 88%), similar to the B–F interaction in Ph_2_BF (*E*_int_ = −605 kcal mol^–1^, *E*_C_ = −530 kcal mol^–1^, 88%). In contrast, the C–O interaction in benzophenone shows reduced ionic contributions at 66% (*E*_int_ = −759 kcal mol^–1^, *E*_C_ = −504 kcal mol^–1^). Thus, the B–O interaction in [(^Mes^Ter)_2_BO]^−^ displays characteristics of both the covalent C=O bond and the ionic B–F bond, highlighting how different theoretical approaches complement each other to provide a comprehensive picture of the bonding situation.

With **5a** and **5b** being rare examples of anionic oxoboranes featuring the unquenched B=O functionality and two flanking aryl groups, close analogies to classic ketones such as benzophenone can be drawn. This naturally prompted the investigation of their reactivity.

**Scheme 5:**
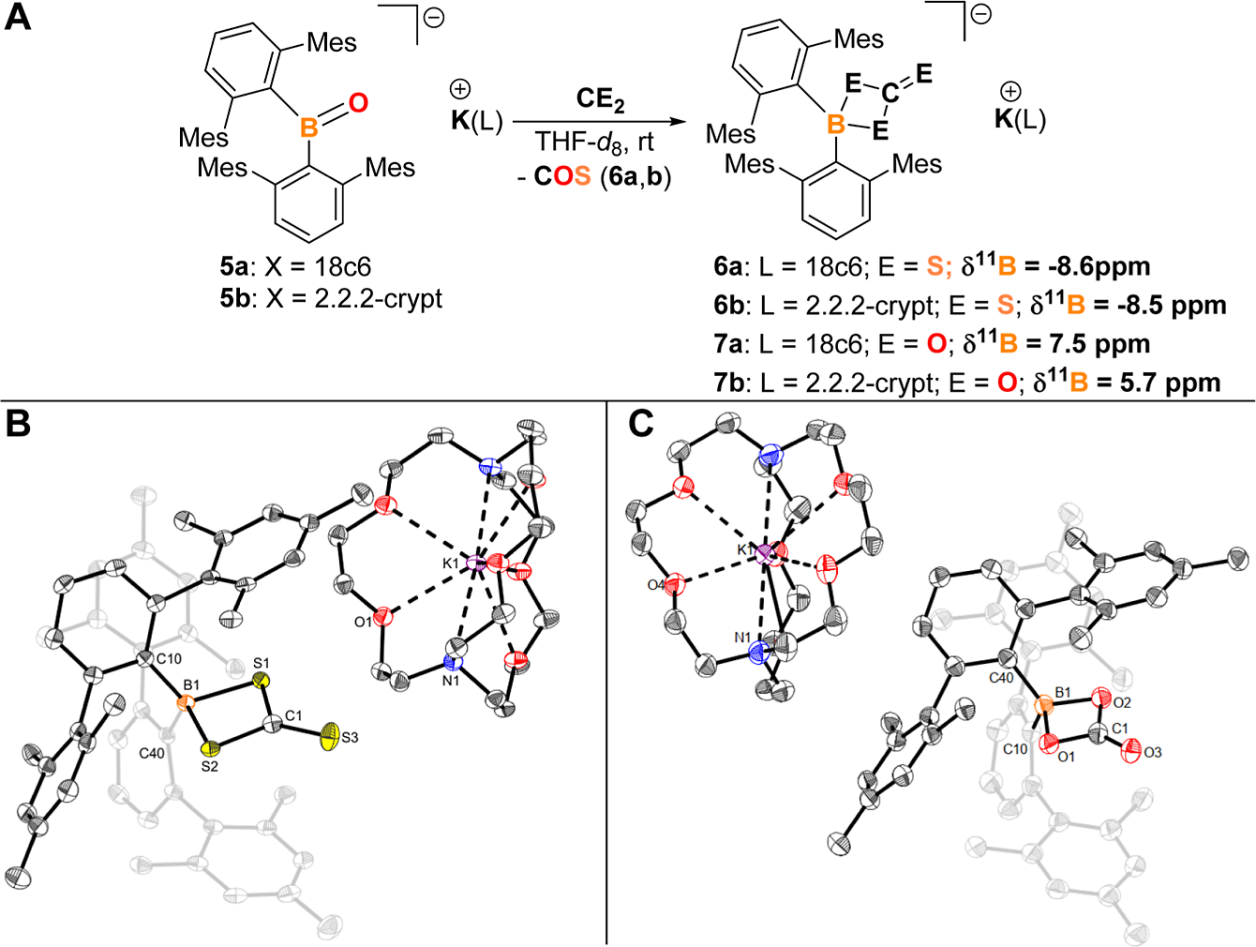
(A) Reaction of **5a,b** with CS_2_ and CO_2_ to Yield [(^Mes^Ter)_2_B-μ-S_2_C=S)][K(L)] (**6a**: L = 18c6*; 6b: L = 2.2.2-Crypt) and [(^Mes^Ter)_2_B-μ-O_2_C = O)][K(L)] (**7a**: L = 18c6; 7b: L = 2.2.2-Crypt) (NMR Chemical Shift Given in THF-*d*_8_ as Solvent and at 298 K); (B) Molecular Structure of [(^Mes^Ter_2_B)-μ-S_2_C=S][K(2.2.2-crypt)] (**6b**) in the Crystal Anisotropic displacement parameters are drawn at the 50% probability level (hydrogen atoms and lattice solvent have been omitted for clarity). Selected bond lengths (Å) and angles (deg): B1–S1 1.979(2), B1–S2 1.984(2), B1–C10 1.646(3), B1–C40 1.651(3), S1–C1 1.7337(19), S2–C1 1.725(2), S3–C1 1.652(2), S1–B1–S2 87.06(9), C10–B1–C40 118.56(15); C: molecular structure of [(^Mes^Ter_2_B)-μ-O_2_C = O][K(2.2.2-crypt)] (**7b**) in the crystal. Anisotropic displacement parameters are drawn at the 50% probability level (hydrogen atoms and lattice solvent have been omitted for clarity). Selected bond lengths (Å) and angles (deg): B1–O1 1.545(5), B1–O2 1.541(5), B1–C10 1.629(5), B1–C40 1.639(5), O1–C1 1.330(4), O2–C1 1.341(5), O3–C1 1.210(5), O1–B1–O2 84.8(2), C10–B1–C40 116.8(3).

Aldridge et al. reported the reactivity of the first nonstabilized *N*-heterocyclic anionic oxoborane toward an excess of carbon disulfide (CS_2_), leading to the formation of the corresponding monomeric thioxoborane anion and carbonyl sulfide (COS).^9b^ By contrast, the reactions of **5a** and **5b** with CS_2_ result in the formation of the first examples of anionic 1,3,2-dithiaboretane-4-thiones, [(^Mes^Ter)_2_B-μ-S_2_C=S][K(X)] (**6a**: X = 18c6; **6b**: X = 2.2.2-crypt), and the identities of **6a** and **6b** were unambiguously verified by SC-XRD (Scheme 5, A,B, and Figure S47). Noteworthy, the formations of **6a** and **6b** are independent of the amount of CS_2_ used, as even an equimolar ratio of CS_2_ results in the formation of **6b** alongside unreacted **5b**. **6a** and **6b** can be understood as [2 + 2] cycloaddition products of CS_2_ and the intermediately formed anionic thioxoborane upon COS release. Indirect evidence for such a cycloaddition-cycloreversion sequence is obtained from the reaction of **5a** and CS_2_ at a certain concentration in C_6_D_6_, which leads to the immediate formation of colorless crystals suitable for single-crystal X-ray diffraction. These crystals reveal the scrambling of oxygen and sulfur across all three positions, characteristic of a compound with the general formula [(^Mes^Ter)_2_B(-μ-S_2-x_O_*x*_)C=(S_1–*x*_O_*x*_)] (Figure S48).^14^ The crystallographically determined ratio between oxygen and sulfur is 0.22:0.78 for the bridging unit and 0.27:0.73 for the exocyclic position.

The molecular structures of the clean B=O/B=S metathesis products **6a** and **6b** reveal boron–sulfide distances of 1.979(2) and 1.984(2) (**6b**), and 1.9824(16) Å (**6a**), respectively, which are consistent with B–S single bond character and elongated compared to the terminal thioxoborane anion by Aldridge and co-workers [1.774(1) Å].^9b^ The four-coordinate boron environment is best described as tetrahedral according to the τ_4_ and τ_4_^′^ structural parameters (0.89 and 0.88 for **6b**, respectively).^24^ The pronounced double bond character of the exocyclic carbon–sulfur bonds in **6a** and **6b** is evident from their significantly shorter bond lengths [1.662(2) Å (**6a**) 1.652(2) Å (**6b**)] compared to the bridging C–S bond lengths [1.7222(13) Å (**6a**); 1.7337(19) Å and (1.725(2) Å (**6b**)]. The ^13^C{^1^H} NMR signal of the S_2_C=S moiety at δ = 236.0 ppm (236.2 ppm for **6a**) and the ^11^B NMR chemical shift of δ = −8.5 ppm (−8.6 ppm for **6a**) also strongly indicate this four-membered ring system. As with **5a** and **5b**, ESI mass spectrometry allows for the quick identification of the separated ion pair in case of **6b**.^14^

CO_2_ was also found to react with **5a** and **5b** to give the corresponding first examples of anionic 1,3,2-dioxaboretane-4-ones [(^Mes^Ter)_2_B-μ-O_2_C=O][K(X)] (**7a**: X = 18c6; **7b**: X = 2.2.2-crypt), which were comprehensively characterized, including determination of the structure of **7b** in the solid state by SC-XRD (Scheme 5, A,C).^14^

The information obtained from the molecular structure of the [2 + 2] cycloaddition product **7b**, formed between the anionic oxoborane moiety of parent **5b** and carbon dioxide, is in good agreement with its heavier sulfur congener **6b**. Accordingly, the boron–oxygen distances of 1.545(5) and 1.541(5) Å are elongated compared to the respective single bond covalent radii (1.48 Å),^20^ due to the four-coordinate and formally negatively charged boron atom. The exocyclic carbon–oxygen bond length of 1.210(5) Å is significantly shorter than those observed within the four-membered heterocycle [1.330(4) and 1.341(5) Å, respectively], indicating a double bond. As expected, the geometry indices τ_4_ and τ_4′_ support the tetrahedral coordination environment at the boron atom (both 0.90).^24^

It is worth noting that classic ketones do neither react with CS_2_ nor CO_2_, demonstrating the drastic influence of the increased basicity of the anionic boron–oxygen moiety.

**Scheme 6:**
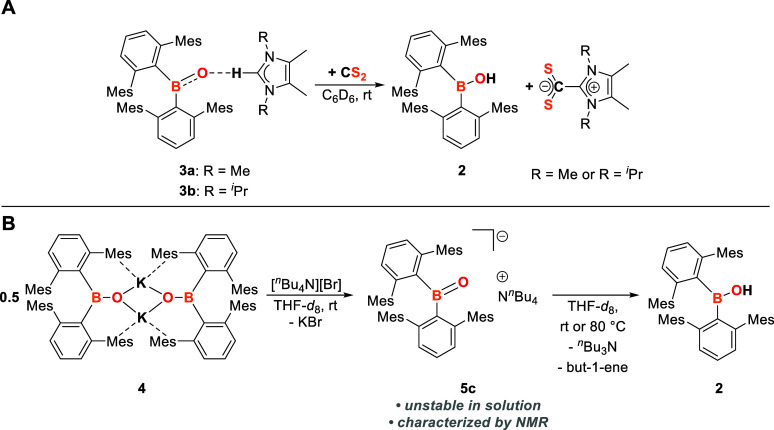
(A) Reaction of **3a,b** with CS_2_ to Give 2 and S_2_CIMe_4_ and S_2_CI^*i*^Pr_2_Me_2_, respectively; (B) In Situ Synthesis of **5c** by Reacting **4** with [^*n*^Bu_4_N][Br] and Its Degradation to Yield 2, ^*n*^Bu_3_N, and But-1-ene

Finally, the necessity of separating the anionic and cationic moieties within these systems to enable metathesis chemistry is confirmed by the reactions of **3a**,**b** with CS_2_. Upon the addition of CS_2_ to **3a**,**b**, a color change to orange/red is observed. Upon storing the reaction mixtures at room temperature, the formation of (^Mes^Ter)_2_BOH (**2**) is clearly indicated by ^1^H NMR spectroscopy (with the characteristic singlet signal of the BOH unit at δ = 6.90 ppm; *cf*. Figure S61), accompanied by the precipitation of red solids. In the case of **3b**, the latter can be recrystallized by slow cooling of the reaction mixtures from +90 °C to room temperature. SC-XRD confirms the formation of the literature-known CS_2_–carbene adduct S_2_CI^*i*^Pr_2_Me_2_ (Scheme 6, **A**).^25^

Additionally, effort to replace the K(L) (L = 2.2.2-crypt or 18c6) countercation in **5a**,**b** to give systems with the simpler tetra-*n*-butylammonium cation were explored by reacting [(^Mes^Ter)_2_BOK]_2_ (**4**) with tetra-*n*-butylammonium bromide in THF-*d*_8_ at room temperature. Subsequent NMR analysis confirmed the formation of the desired anionic oxoborane [(^Mes^Ter)_2_BO][^*n*^Bu_4_N] (**5c**); however, **5c** was proved unstable, degrading relatively quickly, with around 50% remaining after 3 days at room temperature. This Hoffmann type elimination yielded (^Mes^Ter)_2_BOH (**2**), tri-*n*-butylamine, and but-1-ene, as verified by comparison with literature data (Scheme 6, **B**, Figures S63–S66, and Table S1).^26^ This behavior further highlights the inherent basicity of the reported diaryl-substituted anionic oxoboranes, which even trigger the degradation of an ammonium salt at room temperature.

## Conclusion

In this study, we extended the family of anionic oxoboranes to include derivatives that are both isoelectronic and isostructural to classic diaryl-substituted ketones. The key to success lies in the kinetic stabilization of the precursor borinic acid **2** by two sterically demanding terphenyl substituents. This precursor can be synthesized straightforwardly in a one-pot procedure, and its monomeric nature has been verified by SC-XRD. Reactions with selected NHCs yield anionic oxoborane imidazolium salts **3a**,**b**, featuring hydrogen bonding along the B–O···H–C axis. The formation of ion-separated anionic oxoboranes **5a**,**b** is facilitated by a deprotonation followed by sequestration strategy.

Moreover, metathesis chemistry with carbon disulfide has been demonstrated to produce the crystalline anionic 1,3,2-dithiaboretane-4-thiones **6a** and**6b**. This reactivity stands out compared to previously reported anionic oxoboranes, emphasizing the influence of the all-carbon substitution pattern in the systems reported here.

Additionally, the importance of anion–cation separation for enabling metathesis chemistry becomes evident. When the contact ion pairs **3a**,**b** react with carbon disulfide, they form the parent borinic acid **2** and carbon disulfide–NHC adducts instead. Also, the K(L) (L = 2.2.2-crypt or 18c6) counter cations were found to be crucial for accessing long time stable anionic oxoboranes with diaryl substitution pattern as an attempt to synthesize a system with a tetra-*n*-butylammonium countercation was successful, yet proved to be comparatively unstable, degrading to the parent borinic acid **2**, tri-*n*-butylamine and but-1-ene.

Furthermore, **5a**,**b** have been shown to cleanly activate CO_2_ under mild conditions, yielding the first examples of anionic 1,3,2-dioxaboretane-4-ones **7a**,**b**. This further establishes the boron–oxygen linkages in these systems, often considered thermodynamic sinks, as effective platforms for small molecule activation.

The computational analysis of the B–O interaction within anionic oxboranes reveals greater bond polarity compared to the more covalent C–O bond in ketones. This increased polarity, along with the negative charge, accounts for the higher basicity. In this regard, the (formal) B–O double bond is comparable to the recently reported triarylantimony and triarylbismuth oxides Dipp_3_SbO and Dipp_3_BiO, respectively.^4f,4h^

## Experimental Section

All manipulations of air- and moisture-sensitive materials were carried out using standard Schlenk-line and glovebox techniques under an inert atmosphere of argon or dinitrogen. Solvents were purified by a solvent purification system, degassed by sparging with argon and stored over 3 Å molecular sieves. CS_2_ was purchased from commercial suppliers, stirred over molecular sieves, vacuum transferred freeze–pump–thaw degassed three times, and stored over 3 Å molecular sieves prior to use. BF_3_·OEt_2_ and K{N(SiMe_3_)_2_} were purchased from commercial suppliers and used as received. 18c6 and 2.2.2-cryp were obtained from commercial suppliers and recrystallized prior to use. ^Mes^TerLi,^27^ IMe_4_,^28^ and I^*i*^Pr_2_Me_2_^28^ were synthesized according to literature procedures. All reactions were operated under argon atmosphere in an MBraun glovebox with oxygen and water concentrations below 0.1 ppm as monitored by an O_2_/H_2_O Combi-Analyzer if not stated otherwise. NMR spectra were measured in benzene-*d*_6_ (C_6_D_6_) or THF-*d*_8_ (C_4_D_8_O) (dried over CaH_2_, distilled by trap-to-trap transfer in vacuo, degassed by three freeze–pump–thaw cycles and transferred to the glovebox). NMR samples were prepared under argon in NMR tubes with J. Young Teflon valves. NMR spectra were measured on a Bruker AVANCE 400, 500, and 600 MHz spectrometers. ^1^H and ^13^C NMR spectra were referenced internally to residual protio-solvent (^1^H) or solvent (^13^C) resonances (C_6_D_6_: δ_H_ = 7.16 ppm; δ_C_ = 128.06 ppm; C_4_D_8_O: δ_H_ = 3.58 ppm; δ_C_ = 67.21 ppm). Chemical shifts are reported in δ values in ppm relative to tetramethylsilane and referenced using the chemical shift of the solvent ^2^H lock resonance frequency and frequency ratios of 32.083974% for ^11^B and 94.094011% for ^19^F. Mass spectra were recorded using a Bruker APEX IV micrOTOF mass spectrometer and were measured by the Zentrale Massenabteilung (Fakultät für Chemie, Georg-August-Universität Göttingen). Elemental analyses were obtained from the Analytische Labor (Georg-August-Universität Göttingen) using an Elementar Vario EL 3 analyzer. IR spectra of the compounds were measured in the solid state on an Agilent Technologies Cary 630 FTIR spectrometer with Dial Path Technology. Further exact details of syntheses, characterization, crystallographic and computational data are given in the Supporting Information.

## Supporting Information Available

Additional references have been cited within the Supporting Information.^29−47^ General consideration, synthesis and characterization of compounds, crystallographic details, computational details and additional references (PDF)
It contains molecular geometry data, including atomic symbols and their respective X, Y, and Z coordinates in three-dimensional space (XYZ)

## Supplementary Material

ic4c05354_si_001.pdf

ic4c05354_si_002.xyz
