# Supplementary material for: Structural Snapshots on Stepwise Anionic Oxoborane Formation: Access to an Acyclic BO Ketone Analogue and Its Metathesis Chemistry with CO2 and CS2
Source: Inorg Chem. 2025 Feb 5;64(6):3028–37. doi: 10.1021/acs.inorgchem.4c05354 (PMC11836929; doi:10.1021/acs.inorgchem.4c05354)
Supplement: Supplementary file 1 — ic4c05354_si_001.pdf [file ic4c05354_si_001.pdf]

Supporting Information

Structural Snapshots of Stepwise Anionic Oxoborane Formation: Access to an Acyclic BO Ketone Analogue and its Metathesis Chemistry with CO_2 and CS_2

Marius Heitmann,[a] Daniel Duvinage,[b] Christopher Golz,[c] Emanuel Hupf,*[b] Jens Beckmann,*[b] Malte Fischer*[a]

[a] Institut für Anorganische Chemie, Georg-August-Universität Göttingen, Tammannstraße 4, D-37077 Göttingen (Germany)
email: malte.fischer@uni-goettingen.de
[b] Institut für Anorganische Chemie und Kristallographie, Universität Bremen, Leobener Str. 7, D-28359 Bremen (Germany)
[c] Institut für Organische und Biomolekulare Chemie, Georg-August-Universität Göttingen, Tammannstraße 2, D-37077 Göttingen (Germany)
email: emanuel.hupf@uni-bremen.de, j.beckmann@uni-bremen.de

Table of Contents

General Considerations **S2**

Synthesis and Characterization of Compounds **S3**

Crystallographic Details **S56**

Computational Details **S65**

References **S71**

General Considerations

Materials and Synthetic Methods

All manipulations of air- and moisture-sensitive materials were carried out using standard Schlenk-line and glovebox techniques under an inert atmosphere of argon or dinitrogen. Solvents were purified by a Solvent Purification System, degassed by sparging with argon and stored over 3 Å molecular sieves. CS_2 was purchased from commercial suppliers, stirred over molecular sieves, vacuum transferred, freeze-pump-thaw degassed three times, and stored over 3 Å molecular sieves prior to use. BF_3•OEt_2 was purchased from commercial suppliers and used as received. 18c6 and 2.2.2-crypt were obtained from commercial suppliers and recrystallized prior to use. K{N(SiMe$_3$)$_2$} was purchased from commercial sources and used as received. ^{Mes}TerLi,[S1] IMe$_4$,[S2] and I^{i}Pr$_2$Me$_2$[S2] were synthesized according to literature procedures. All reactions were operated under an argon atmosphere in an MBraun glovebox with oxygen and water concentrations below 0.1 ppm as monitored by an O_2/H_2O Combi-Analyzer if not stated otherwise.

Caution! BF_3•OEt_2 is a flammable liquid with the potential to cause fires and explosions. It may react with water to produce hydrofluoric acid, which is corrosive to the skin and eyes. Therefore, the reactions were performed on a comparatively small scale and quenched with an excess of $CaCl_2$ to bind the potentially formed HF as CaF_2.

Analytical Methods

NMR spectra were measured in benzene-d_6 (C_6D_6) or THF-d_8 (C_4D_8O) (dried over CaH_2, distilled by trap-to-trap transfer in vacuo, degassed by three freeze-pump-thaw cycles and transferred to the glovebox). NMR samples were prepared under argon in NMR tubes with J. Young Teflon valves. NMR spectra were measured on a Bruker Avance 400 MHz, 500 MHz, and 600 MHz spectrometers. ^{1}H and ^{13}C NMR spectra were referenced internally to residual protio-solvent (^{1}H) or solvent (^{13}C) resonances (C_6D_6: δ_H = 7.16 ppm; δ_C = 128.06 ppm; C_4D_8O: δ_H = 3.58 ppm; δ_C = 67.21 ppm). Chemical shifts are reported in δ values in ppm relative to tetramethylsilane and referenced using the chemical shift of the solvent ^{2}H lock resonance frequency and frequency ratios of 32.083974% for ^{11}B and 94.094011% for ^{19}F. Mass spectra were recorded using a Bruker APEX IV micrOTOF mass spectrometer and were measured by the Zentrale Massenabteilung (Fakultät für Chemie, Georg-August-Universität Göttingen). Elemental analyses were obtained from the Analytische Labor (Georg-August-Universität Göttingen) using an Elementar Vario EL 3 analyzer. IR spectra of the compounds were measured in the solid state on an Agilent Technologies Cary 630 FTIR spectrometer with Dial Path Technology.

Synthesis and Characterization of Compounds

Synthesis of (^{Mes}Ter)$_2$BOH (2)

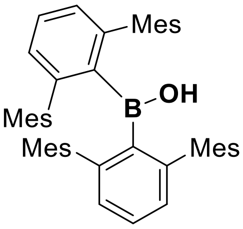

A solution of ^{Mes}TerLi (0.500 g, 1.561 mmol) in 10 mL of Et_2O was prepared, and BF_3•OEt_2 (0.31 mL, 2.497 mmol) was added dropwise at 0 °C. The reaction mixture was stirred overnight at room temperature. Subsequently, all volatile components were removed under vacuum, and the crude product was analysed using multinuclear NMR spectroscopy to confirm the formation of (^{Mes}Ter)BF_2 (Figure S1-S4). Next, 20 mL of *n*-hexane and ^{Mes}TerLi (0.500 g, 1.561 mmol) were added, and the reaction mixture was stirred for 72 h at room temperature. After this period, all volatile components were removed under vacuum, and the crude (^{Mes}Ter)$_2$BF (**1**)[S3] was analysed by multinuclear NMR spectroscopy (Figure S5-S8). Without further purification, 8 mL of DCM and 2 mL of distilled H_2O were added to the mixture. The reaction mixture was stirred overnight at room temperature. $CaCl_2$ was then added to act as a drying agent and to bind any formed HF as CaF_2. The organic phase was filtered and dried under vacuum to yield crude (^{Mes}Ter)$_2$BOH (**2**). The resulting solid was recrystallized twice by layering concentrated DCM solutions of **2** with *n*-hexane. These crystals were found to be suitable for single crystal X-ray diffraction. Notably, two different polymorphs of **2** were identified (Table S2). Significant differences were observed in the C–B–C angles, with a variation of 7.81° (polymorph a (*Fdd*2) 132.67(3)°; polymorph b ($P2_1/c$) 124.86(1)°), and in the O–H bond lengths, differing by 0.08 Å (polymorph a (*Fdd*2) 0.894 Å; polymorph b ($P2_1/c$) 0.978 Å).
(^{Mes}Ter)$_2$BOH (**2**) can also be purified by column chromatography, a step that was usually carried out using fractions collected after the twofold crystallization described above [**R$_f$**(CH_2Cl_2:Hexanes = 1:7) = 0.33].

Yield: 0.745 g (1.138 mmol; 73%).
^{1}H NMR (400 MHz, C_6D_6, 298 K): δ = 1.81 (s, 24H, *o*-C*H*$_3$-C_6H_3), 2.23 (s, 12H, *p*-C*H*$_3$-C_6H_3), 6.65 (d, $^3J_{H,H}$ = 7.6 Hz, 4H, *m*-CH$_{Aryl}$-B), 6.77 (s, 8H, *m*-C*H*$_{Aryl}$-C_6H_3), 6.90 (s, 1H, BOH), 6.95 (t, $^3J_{H,H}$ = 7.6 Hz, 2H, *p*-CH$_{Aryl}$-B) ppm.
^{1}H NMR (400 MHz, THF-d_8, 298 K): δ = 1.59 (s, 24H, *o*-C*H*$_3$-C_6H_3), 2.28 (s, 12H, *p*-C*H*$_3$-C_6H_3), 6.58 (d, $^3J_{H,H}$ = 7.6 Hz, 4H, *m*-CH$_{Aryl}$-B), 6.73 (s, 8H, *m*-C*H*$_{Aryl}$-C_6H_3), 6.78 (s, 1H, BOH), 7.13 (t, $^3J_{H,H}$ = 7.6 Hz, 2H, *p*-CH$_{Aryl}$-B) ppm.
^{13}C{^{1}H} NMR (126 MHz, C_6D_6, 298 K): δ = 21.1 (*p*-$\underline{C}H_3$-C_6H_3), 22.2 (*o*-$\underline{C}H_3$-C_6H_3), 128.5 (*p*-CH$_{Aryl}$-B), 129.1 (*m*-$\underline{C}H_{Aryl}$-C_6H_3), 130.5 (*m*-CH$_{Aryl}$-B), 136.6 ($C_{q,Aryl}$), 137.4 ($C_{q,Aryl}$), 139.7 (br, $C_{q,Aryl}$B), 141.4 ($C_{q,Aryl}$), 146.7 ($C_{q,Aryl}$) ppm.
^{13}C{^{1}H} NMR (126 MHz, THF-d_8, 298 K): δ = 21.0 (*p*-$\underline{C}H_3$-C_6H_3), 22.1 (*o*-$\underline{C}H_3$-C_6H_3), 128.6 (*p*-CH$_{Aryl}$-B), 129.1 (*m*-$\underline{C}H_{Aryl}$-C_6H_3), 130.7 (*m*-CH$_{Aryl}$-B), 136.7 ($C_{q,Aryl}$), 137.6 ($C_{q,Aryl}$), 140.0 (br, $C_{q,Aryl}$B), 141.8 ($C_{q,Aryl}$), 147.1 ($C_{q,Aryl}$) ppm.
^{11}B/^{11}B{^{1}H} NMR (96 MHz, C_6D_6, 298 K): δ = 49.9 ppm.
^{11}B/^{11}B{^{1}H} NMR (96 MHz, THF-d_8, 298 K): δ = 46.9 ppm.
HR/MS: m/z calcd.: 677.3933 [M+Na$^+$]; measured (ESI, positive): m/z = 677.3943.
EA: Anal. calcd. for $C_{48}H_{51}BO$: C, 88.05; H, 7.85; Found: C, 87.52; H, 7.91.

Note: The NMR data of (^{Mes}Ter)BF_2 and (^{Mes}Ter)$_2$BF were previously reported in CD_2Cl_2 as solvent.[S3]
NMR data of ^{Mes}TerBF$_2$:

^{1}H NMR (400 MHz, C_6D_6, 298 K): δ = 2.09 (s, 12H, *o*-C*H*$_3$-C_6H_3), 2.15 (s, 6H, *p*-C*H*$_3$-C_6H_3), 6.83 (s, 4H, *m*-C*H*$_{Aryl}$-C_6H_3), 6.93 (d, $^{3}J_{H,H}$ = 7.6 Hz, 2H, *m*-CH$_{Aryl}$-B), 7.24 (t, $^{3}J_{H,H}$ = 7.6 Hz, 2H, *p*-CH$_{Aryl}$-B) ppm.
^{13}C{^{1}H} NMR (126 MHz, C_6D_6, 298 K): δ = 20.6 (*o*-<u>C</u>H$_3$-C_6H_3), 21.2 (*p*-<u>C</u>H$_3$-C_6H_3), 127.6 (*m*-CH$_{Aryl}$-B), 128.7 (*m*-<u>C</u>H$_{Aryl}$-C_6H_3), 131.6 (*p*-CH$_{Aryl}$-B), 136.0 ($C_{q,Aryl}$), 137.5 ($C_{q,Aryl}$), 138.5 ($C_{q,Aryl}$), 146.4 ($C_{q,Aryl}$) ppm. Note: $C_{q,Aryl}$B was not observed.
^{11}B/^{11}B{^{1}H} NMR (96 MHz, C_6D_6, 298 K): δ = 26.1 ppm.
^{19}F/^{19}F{^{1}H} NMR (377 MHz, C_6D_6, 298 K): δ = -69.4 ppm.

NMR data of ^{Mes}Ter$_2$BF (1):
^{1}H NMR (400 MHz, C_6D_6, 298 K): δ = 1.83 (s, 24H, *o*-C*H*$_3$-C_6H_3), 2.24 (s, 12H, *p*-C*H*$_3$-C_6H_3), 6.63 (d, $^{3}J_{H,H}$ = 7.6 Hz, 4H, *m*-CH$_{Aryl}$-B), 6.83 (s, 8H, *m*-C*H*$_{Aryl}$-C_6H_3), 6.92 (t, $^{3}J_{H,H}$ = 7.6 Hz, 2H, *p*-CH$_{Aryl}$-B) ppm.
^{13}C{^{1}H} NMR (126 MHz, C_6D_6, 298 K): δ = 21.2 (*p*-<u>C</u>H$_3$-C_6H_3), 22.32 (*o*-<u>C</u>H$_3$-C_6H_3), 22.34 (*o*-<u>C</u>H$_3$-C_6H_3), 128.7 (*m*-<u>C</u>H$_{Aryl}$-C_6H_3), 129.8 (*m*-<u>C</u>H$_{Aryl}$-C_6H_3), 130.4 (*m*-<u>C</u>H$_{Aryl}$-C_6H_3), 136.3 ($C_{q,Aryl}$), 137.3 ($C_{q,Aryl}$), 140.9 ($C_{q,Aryl}$), 147.6 (d, $J_{F,C}$ = 4.3 Hz, $C_{q,Aryl}$) ppm. Note: $C_{q,Aryl}$B could not be observed.
^{11}B/^{11}B{^{1}H} NMR (96 MHz, C_6D_6, 298 K): δ = 49.4 ppm.
^{19}F/^{19}F{^{1}H} NMR (377 MHz, C_6D_6, 298 K): δ = 18.6 ppm.

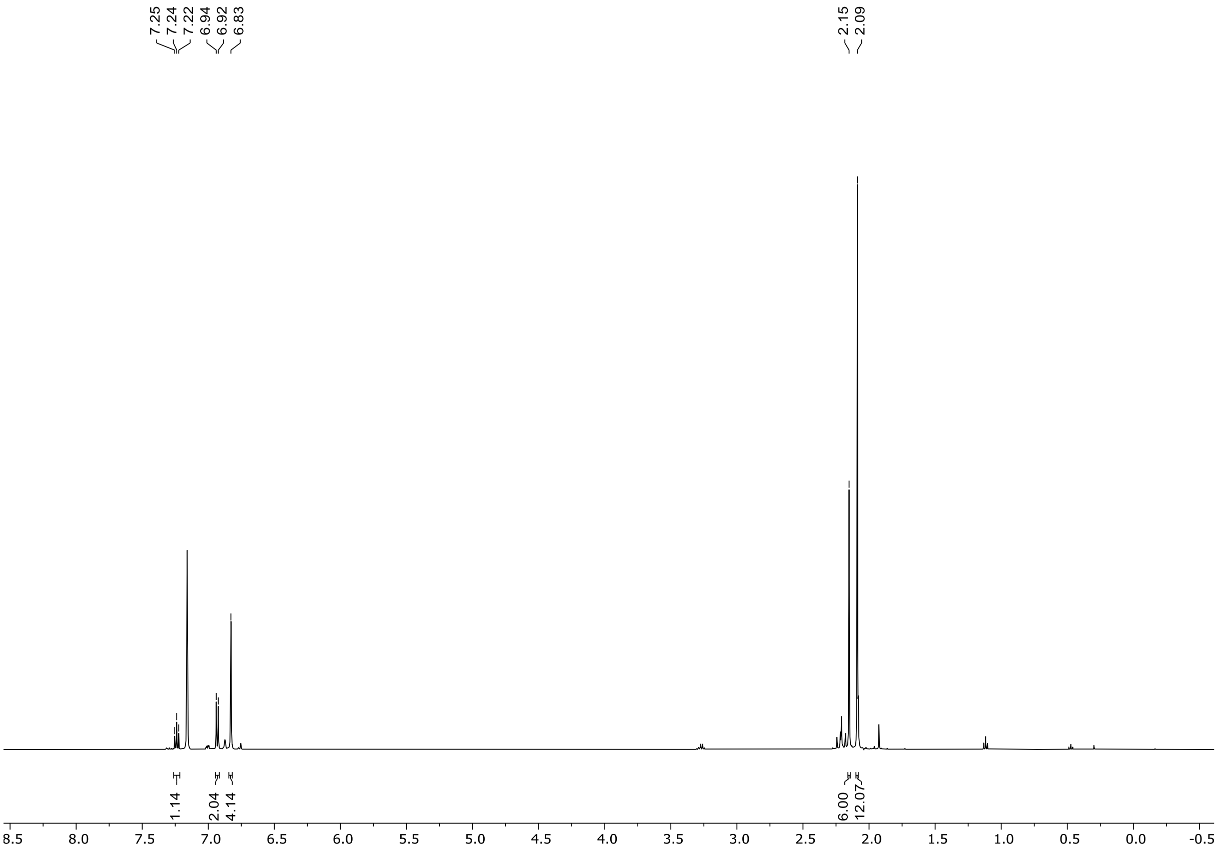

Figure S1. ^{1}H NMR spectrum of crude (^{Mes}Ter)BF_2 (400 MHz, C_6D_6, 298 K).

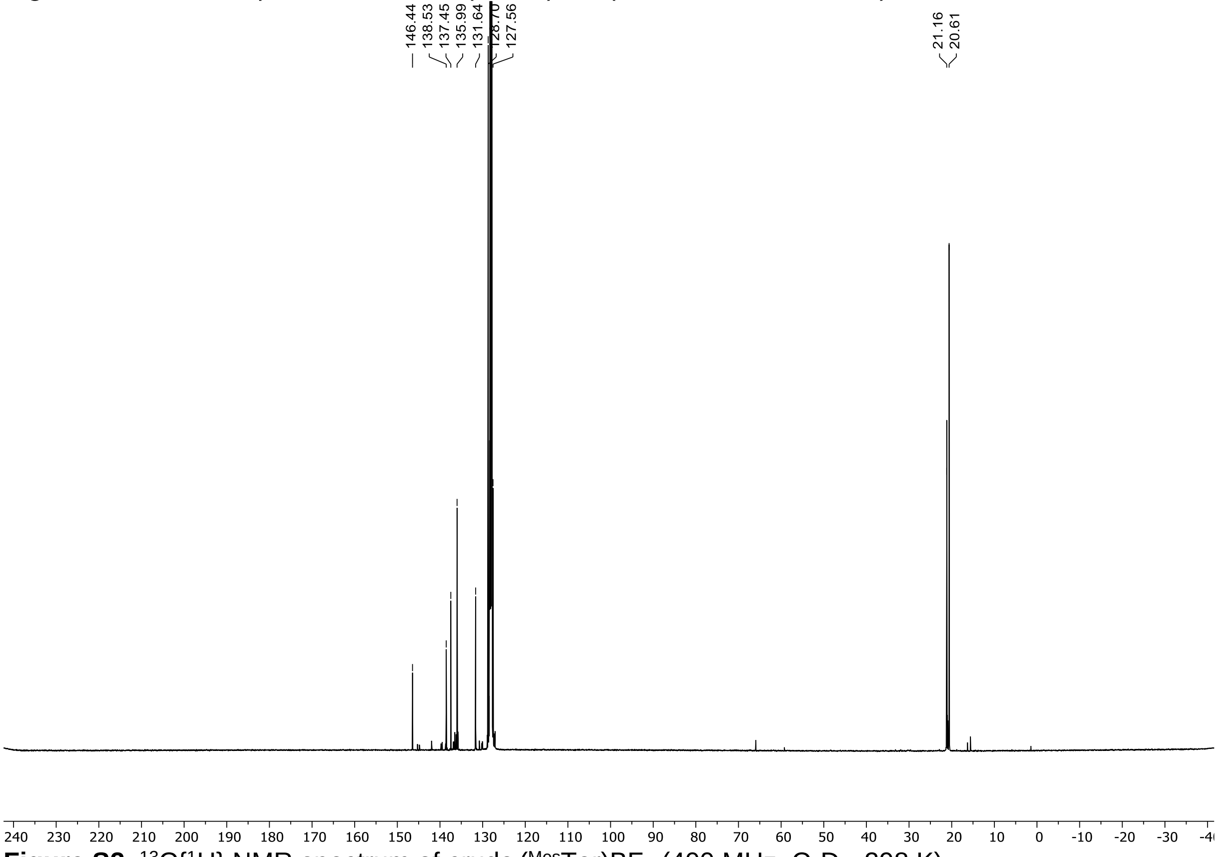

Figure S2. ^{13}C{^{1}H} NMR spectrum of crude (^{Mes}Ter)BF_2 (400 MHz, C_6D_6, 298 K).

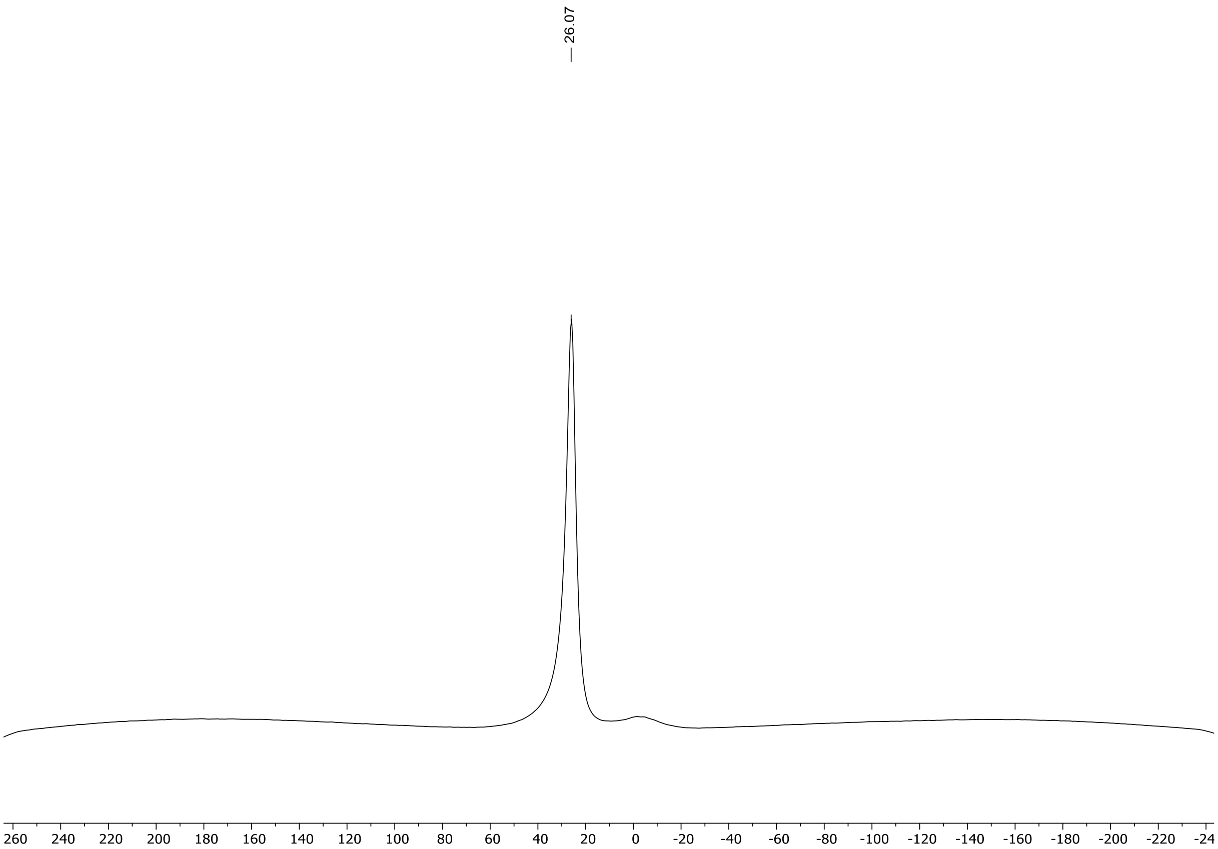

Figure S3. $^{11}B\{^{1}H\}$ NMR spectrum of crude (^{Mes}Ter)BF_2 (96 MHz, C_6D_6, 298 K).

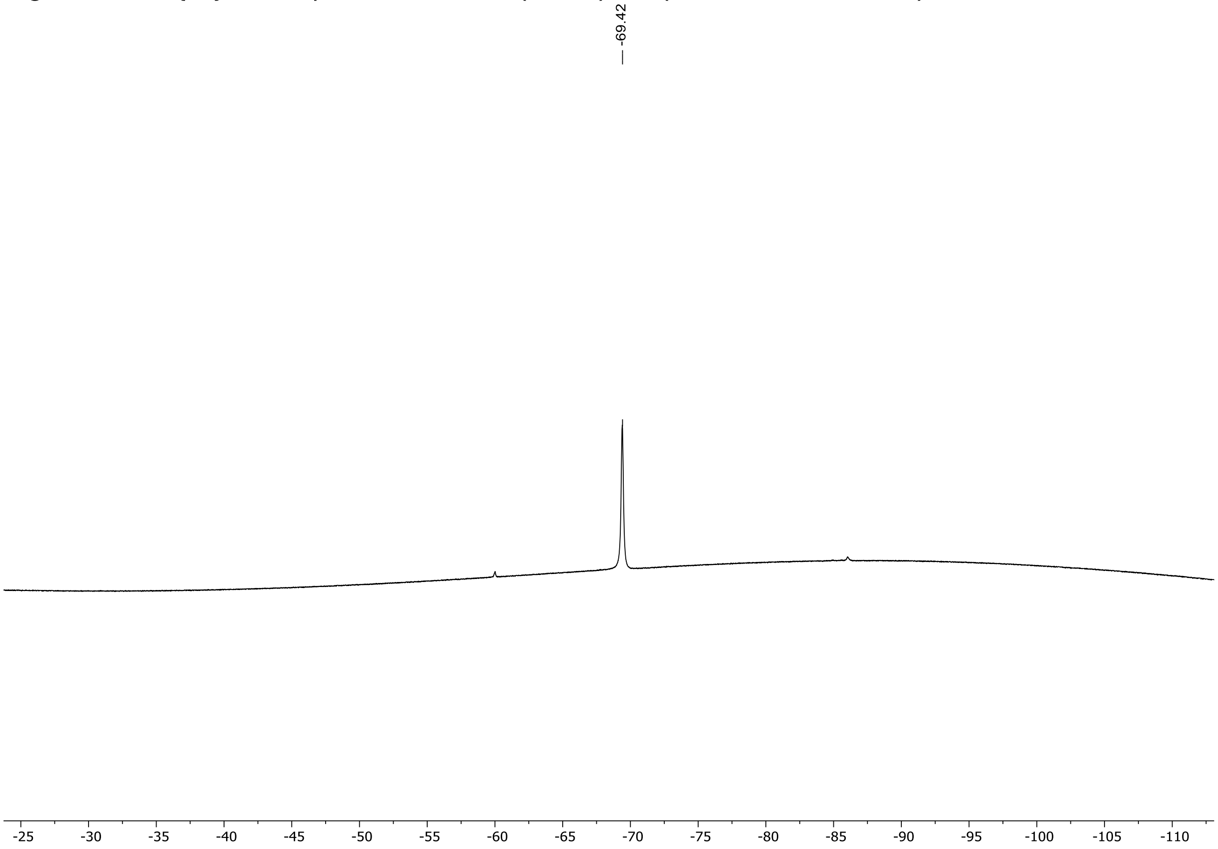

Figure S4. $^{19}F\{^{1}H\}$ NMR spectrum of crude (^{Mes}Ter)BF_2 (96 MHz, C_6D_6, 298 K).

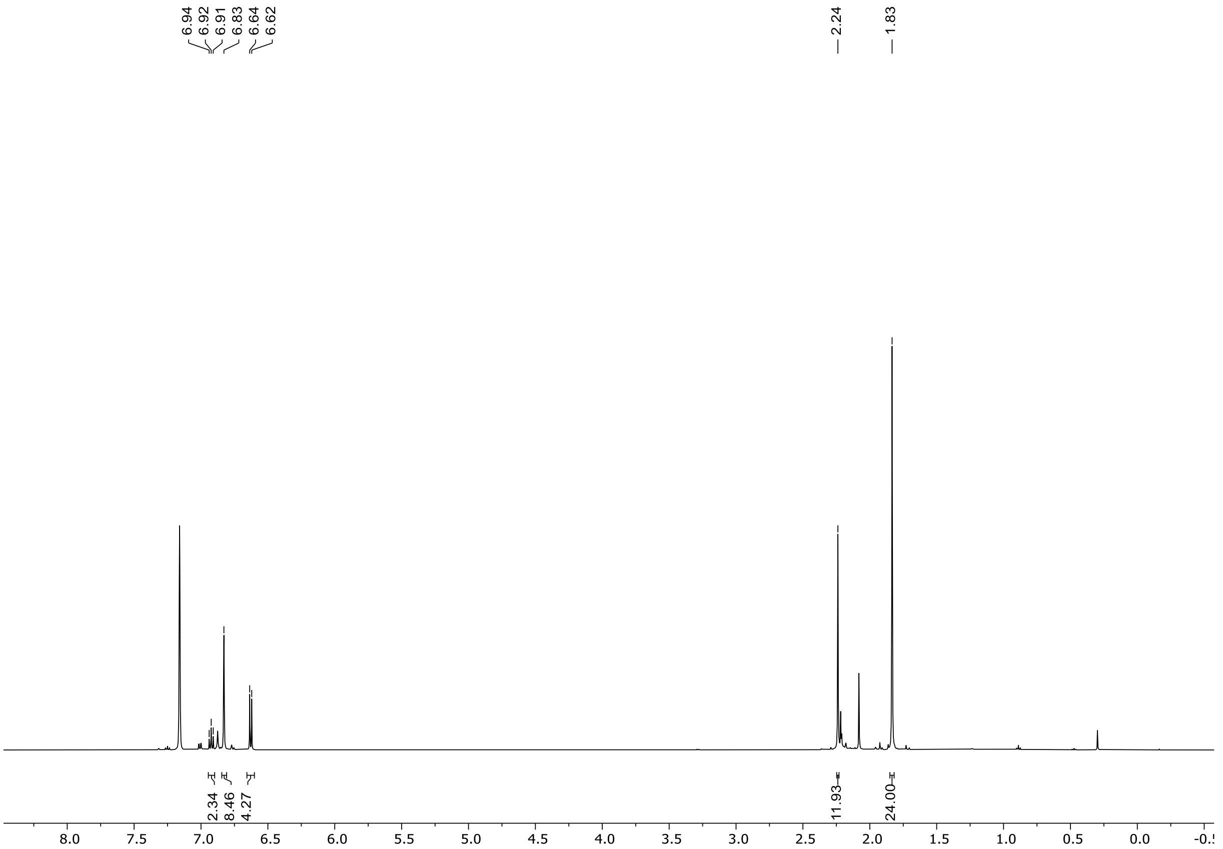

Figure S5. ^{1}H NMR spectrum of crude ($^{Mes}Ter)_2BF$ (**1**) (400 MHz, C_6D_6, 298 K).

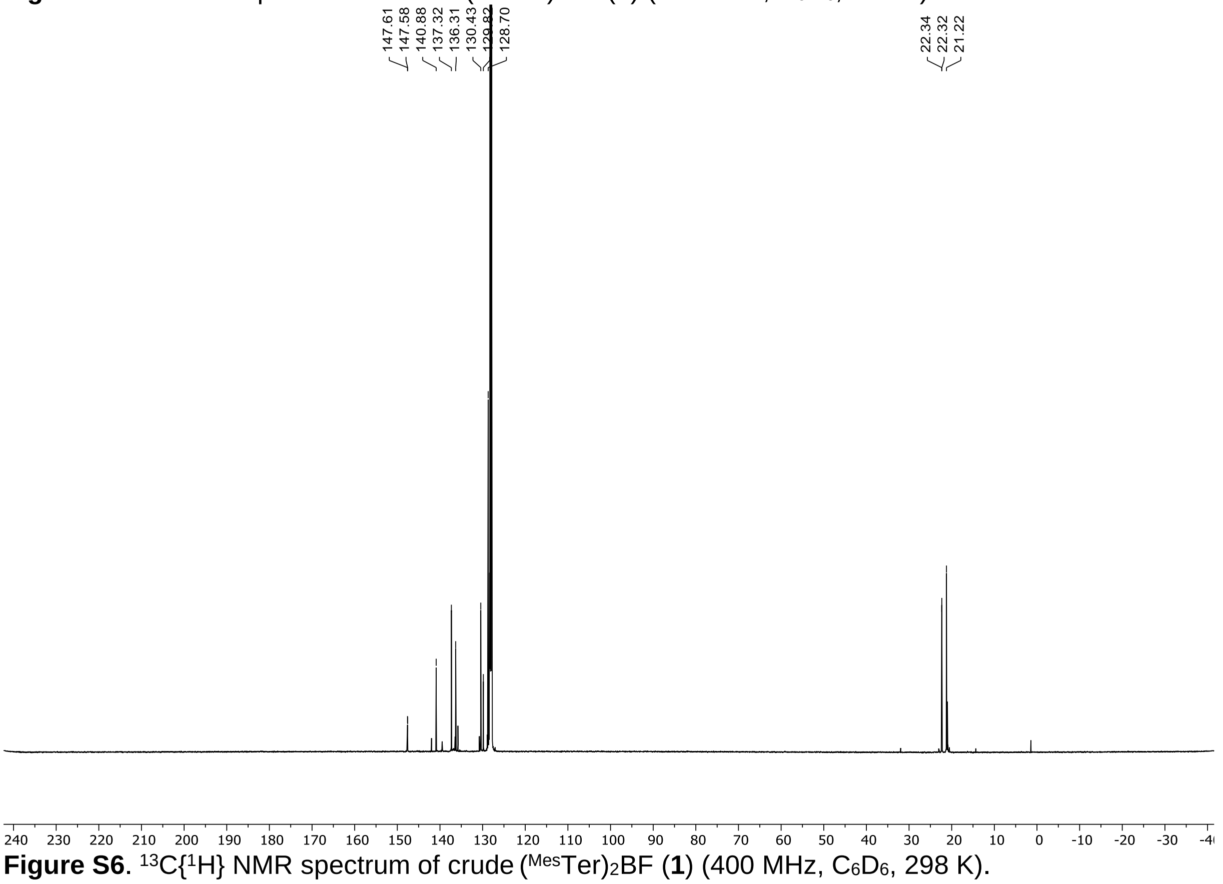

Figure S6. $^{13}C\{^{1}H\}$ NMR spectrum of crude ($^{Mes}Ter)_2BF$ (**1**) (400 MHz, C_6D_6, 298 K).

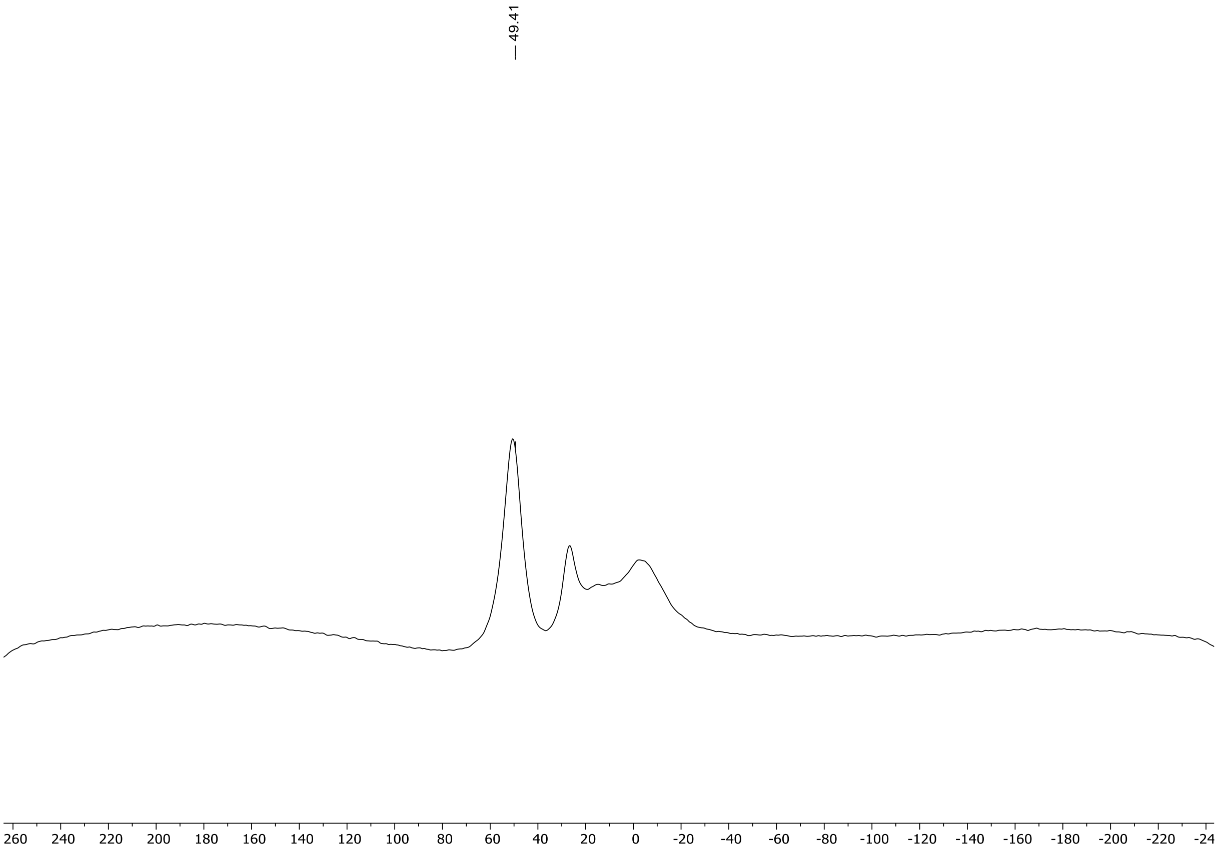

Figure S7. $^{11}B\{^1H\}$ NMR spectrum of crude (^{Mes}Ter)$_2$BF (**1**) (96 MHz, C_6D_6, 298 K); the other observed signals correspond to unknown impurities and the glass background signal.

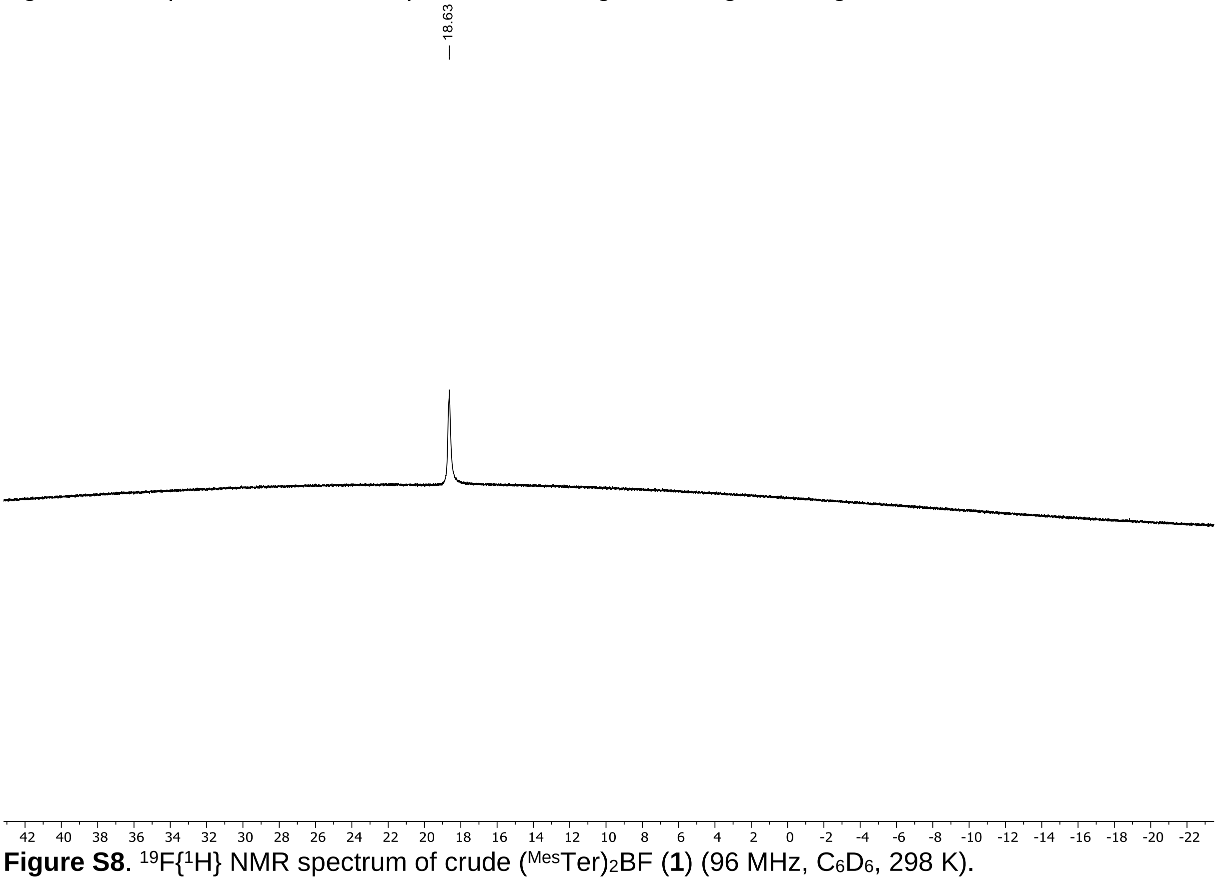

Figure S8. $^{19}F\{^1H\}$ NMR spectrum of crude (^{Mes}Ter)$_2$BF (**1**) (96 MHz, C_6D_6, 298 K).

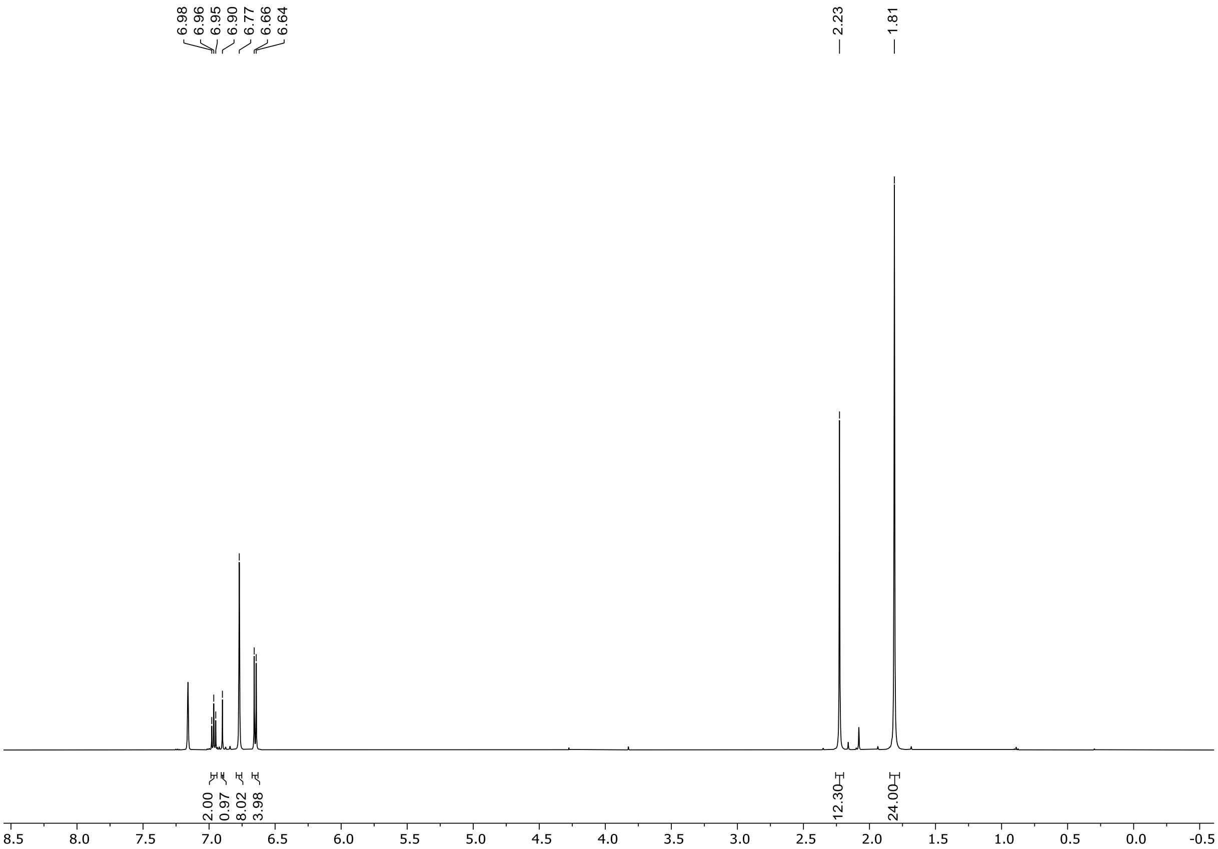

Figure S9. ^{1}H NMR spectrum of (^{Mes}Ter)$_2$BOH (**2**) (400 MHz, C_6D_6, 298 K).

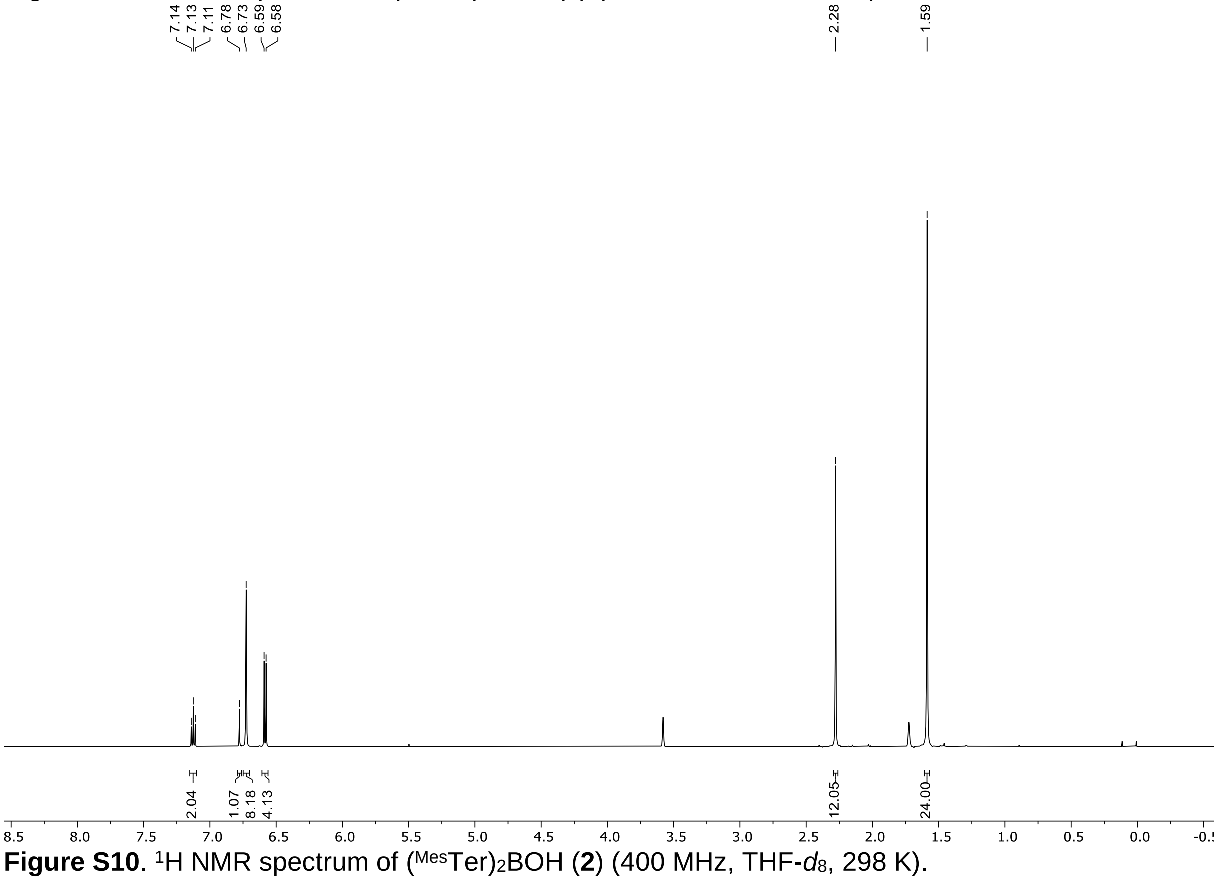

Figure S10. ^{1}H NMR spectrum of (^{Mes}Ter)$_2$BOH (**2**) (400 MHz, THF-d_8, 298 K).

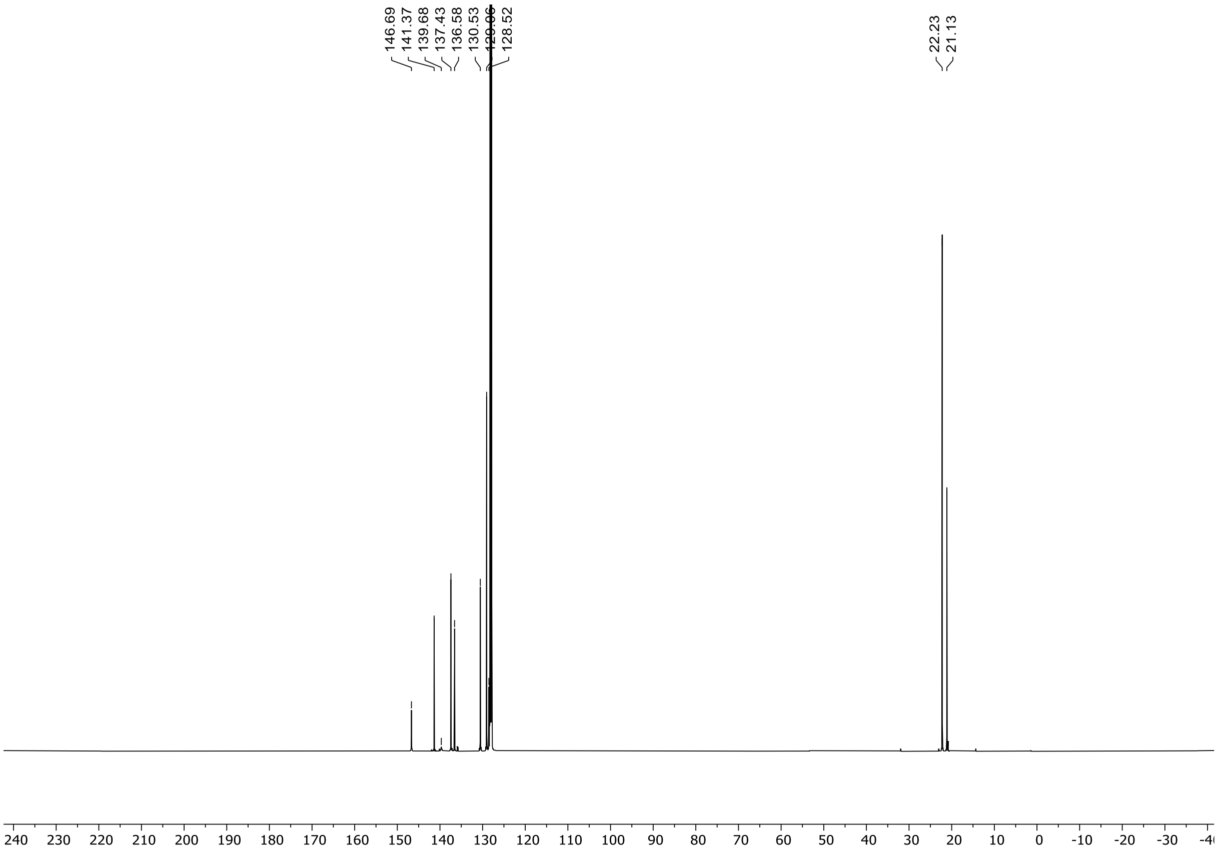

Figure S11. $^{13}C\{^{1}H\}$ NMR spectrum of (^{Mes}Ter)$_2$BOH (**2**) (126 MHz, C_6D_6, 298 K).

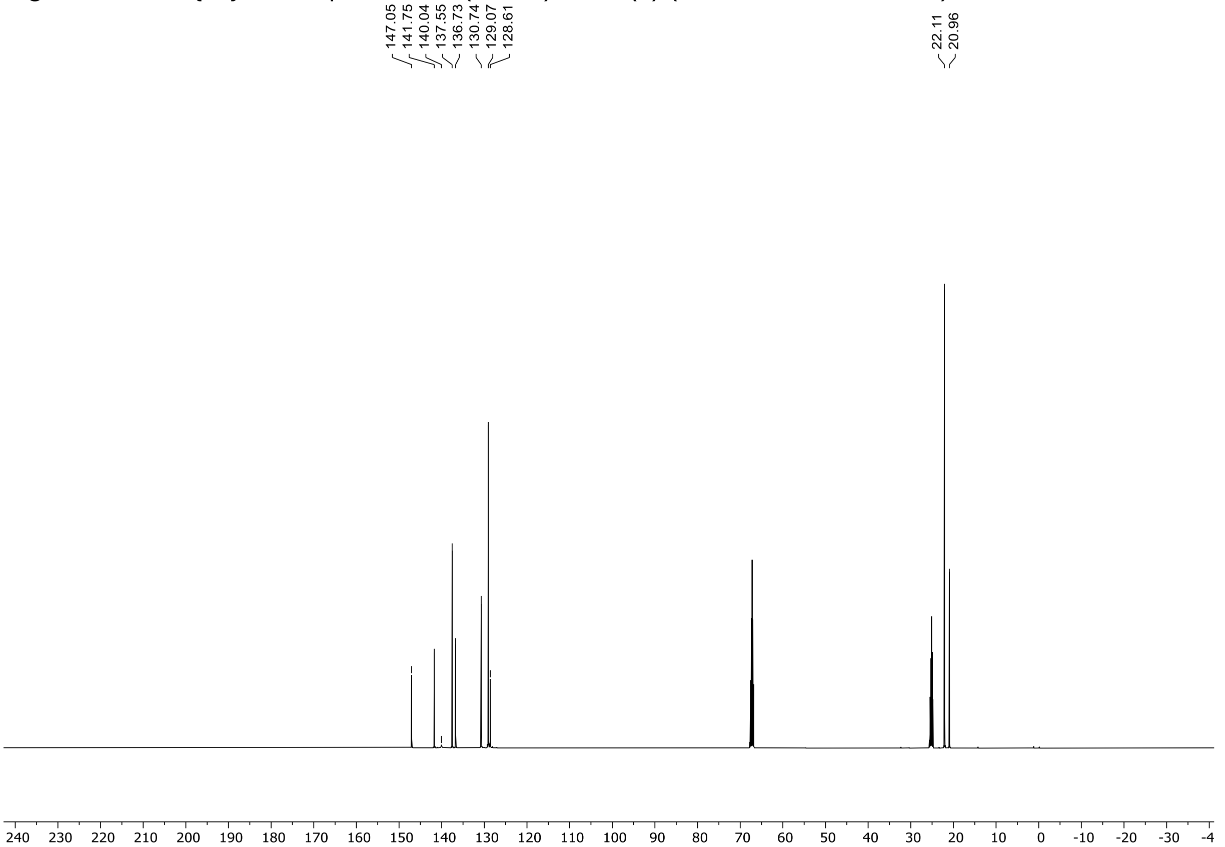

Figure S12. $^{13}C\{^{1}H\}$ NMR spectrum of (^{Mes}Ter)$_2$BOH (**2**) (126 MHz, THF-d_8, 298 K).

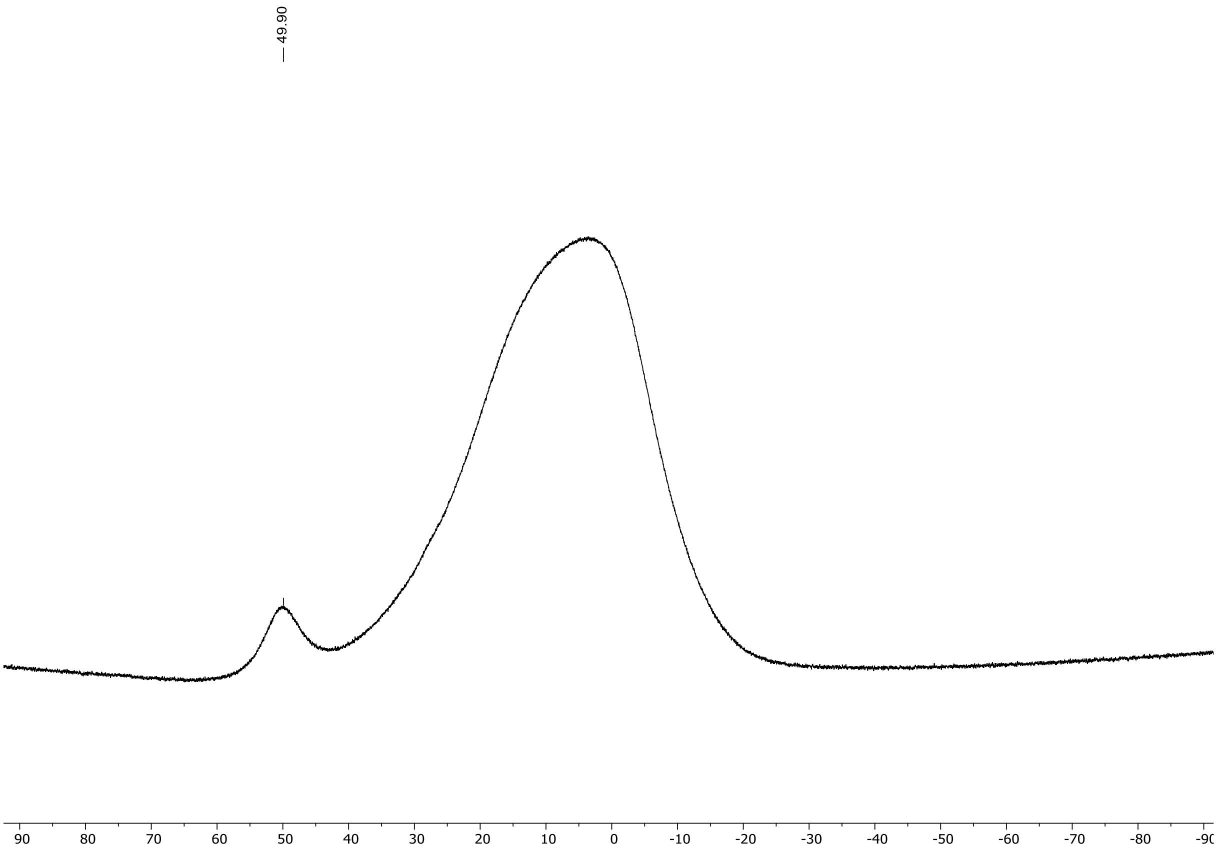

Figure S13. $^{11}B\{^{1}H\}$ NMR spectrum of (^{Mes}Ter)$_2$BOH (**2**) (96 MHz, C_6D_6, 298 K).

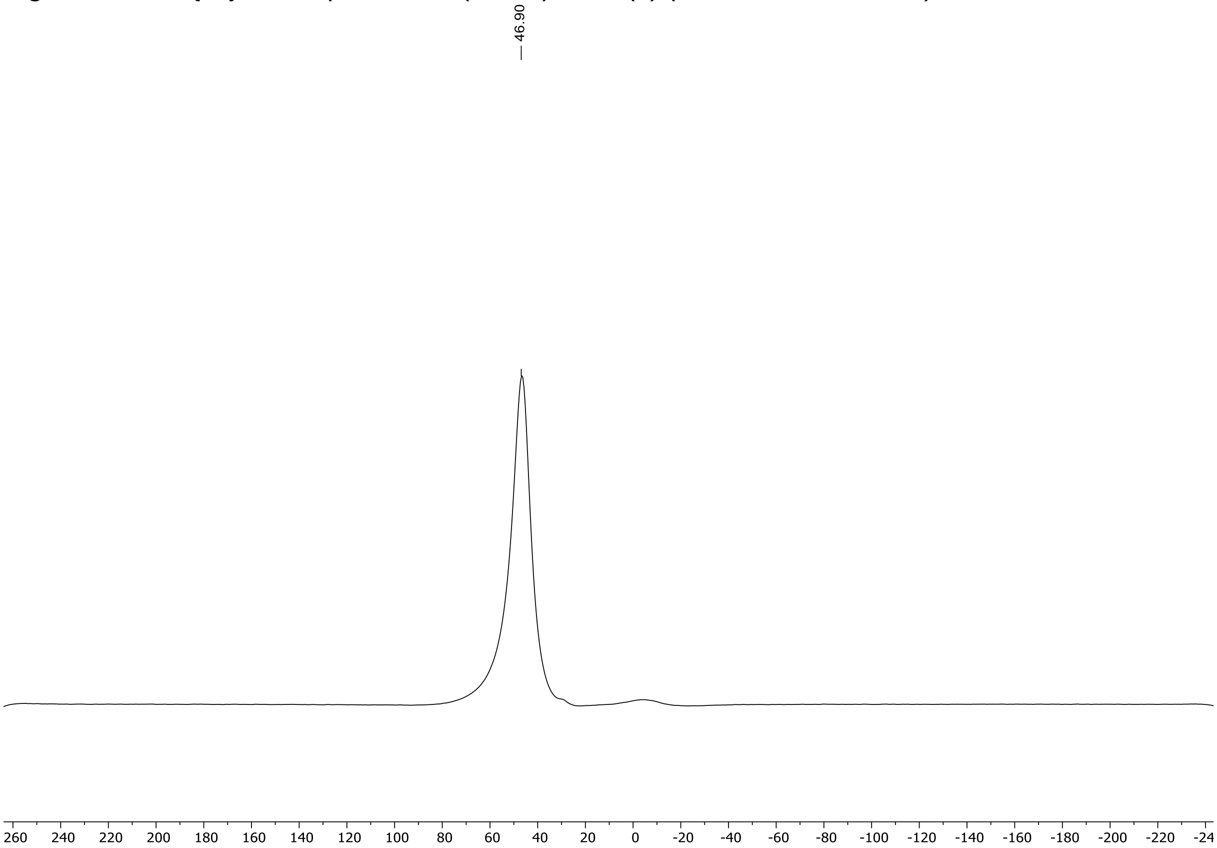

Figure S14. $^{11}B\{^{1}H\}$ NMR spectrum of (^{Mes}Ter)$_2$BOH (**2**) (96 MHz, THF-d_8, 298 K).

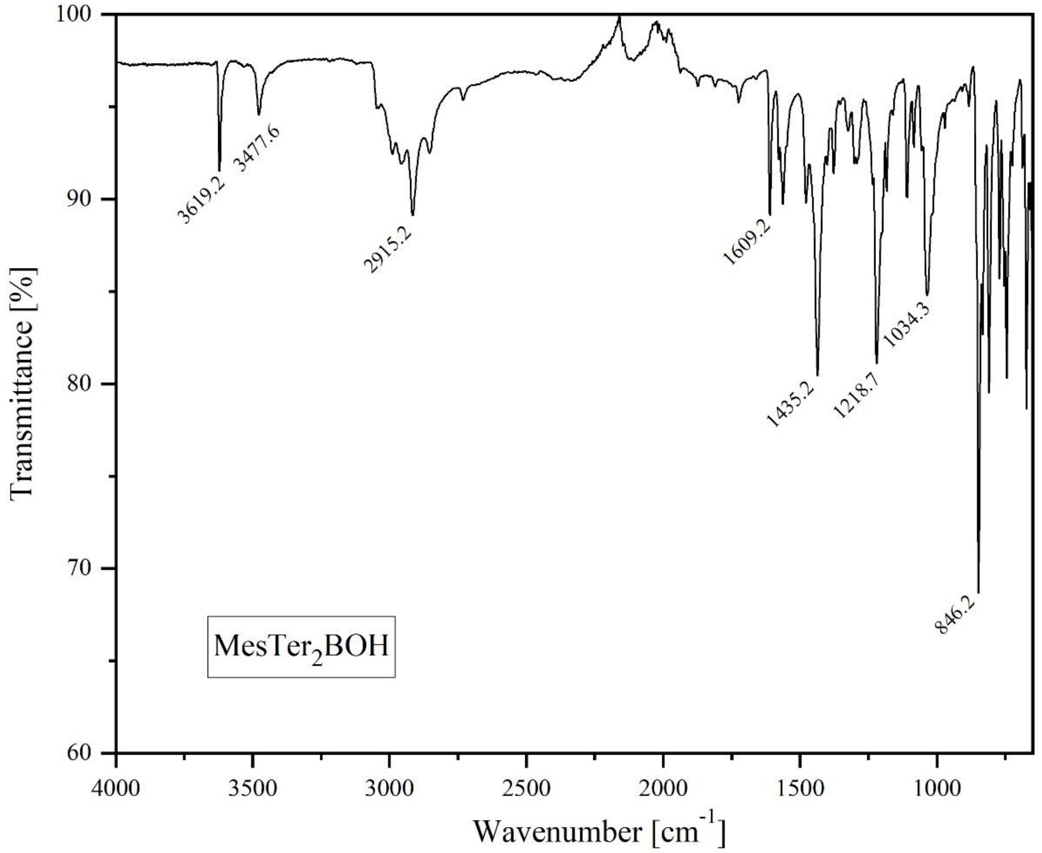

Figure S15. IR spectrum of (^{Mes}Ter)$_2$BOH (**2**); Note: despite multiple measurements of **2** dried rigorously under high vacuum, two peaks attributed to O–H bond stretching frequencies are measured. We attribute this to the presence of two observed distinct polymorphs, which primarily differ in their C–B–C angles and O–H bond lengths (*vide supra*), leading to variations in force constants and acidities in the solid state.

IR (ATR): $\tilde{\nu}$ [cm^{-1}] = 3621 (s), 3478 (m), 3047 (w), 2990 (w), 2957 (w), 2915 (m), 2854 (w), 1937 (w), 1873 (w), 1724 (w), 1609 (m), 1562 (m), 1477 (w), 1435 (s), 1400 (w), 1376 (w), 1323 (w), 1300 (w), 1291 (w), 1219 (s), 1182 (w), 1161 (w), 1108 (m), 1083 (w), 1034 (s), 970 (w), 846 (s), 832 (w), 808 (s), 769 (m), 744 (s), 724 (w), 685 (w), 672 (m).

Synthesis of [^{Mes}Ter$_2$BO][HIMe$_4$] (3a)

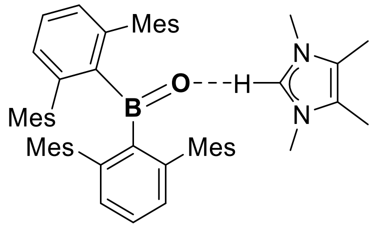

^{Mes}Ter$_2$BOH (0.030 g, 0.046 mmol) was dissolved in 0.3 mL of C_6D_6. Subsequently, IMe$_4$ (0.006 g, 0.046 mmol) in 0.3 mL of C_6D_6 was added to the solution, and the reaction mixture was analysed by ^{1}H NMR spectroscopy, which revealed the clean formation of [^{Mes}Ter$_2$BO][HIMe$_4$] (**3a**) (Figure S16). All volatile components were then removed under vacuum, yielding [^{Mes}Ter$_2$BO][HIMe$_4$] (**3a**) as a colourless solid. Crystals suitable for single crystal X-ray diffraction were obtained from a saturated Et_2O solution of **3a** at -30 °C.

Yield: 0.031 g (0.040 mmol; 87%).
^{1}H NMR (400 MHz, C_6D_6, 298 K): δ = 1.21 (s, 6H, NC$_q$CH$_3$), 2.02 (s, 24H, *o*-C*H*$_3$-C$_6$H$_3$), 2.24 (s, 12H, *p*-C*H*$_3$-C$_6$H$_3$), 2.94 (s, 6H, NCH$_3$), 6.70 (s, 8H, *m*-C*H*$_{Aryl}$-C$_6$H$_3$), 6.80 (d, $^3J_{H,H}$ = 7.6 Hz, 4H, *m*-CH$_{Aryl}$-B), 7.10 (t, $^3J_{H,H}$ = 7.5 Hz, 2H, *p*-CH$_{Aryl}$-B), 11.66 (s(br), 1H, HC$_{Im}$) ppm.
^{13}C{^{1}H} NMR (126 MHz, C_6D_6, 298 K): δ = 7.2 (NC$_q\underline{C}$H$_3$), 21.2 (*p*-$\underline{C}$H$_3$-C$_6$H$_3$), 22.9 (*o*-$\underline{C}$H$_3$-C$_6$H$_3$), 32.8 (NCH$_3$), 124.0 (N$\underline{C}_q$CH$_3$), 125.8 (*p*-CH$_{Aryl}$-B), 128.0 (*m*-$\underline{C}$H$_{Aryl}$-C$_6$H$_3$)*, 129.8 (*m*-CH$_{Aryl}$-B), 134.4 (C$_{q,Aryl}$), 137.9 (C$_{q,Aryl}$), 144.9 (C$_{q,Aryl}$), 146.1 (C$_{q,Aryl}$), 148.1 (br, HCN$_2$), 149.1 (br, C$_{q,Aryl}$B) ppm.
* = overlap with C_6D_6 signal and assigned by ^{1}H/^{13}C-HSQC
^{11}B/^{11}B{^{1}H} NMR (96 MHz, C_6D_6, 298 K): δ = 39.1 ppm.
HR/MS: m/z calcd.: 653.3968 [$C_{48}H_{50}BO]^-$ measured (ESI, negative): m/z 653.3966.
HR/MS: m/z calcd.: 125.1073 [$C_7H_{13}N_2]^+$ measured (ESI, positive): m/z 125.1077.
EA: Anal. calcd. for $C_{55}H_{63}BN_2O$: C, 84.81; H, 8.15; N, 3.60; Found: C, 83.77; H, 8.03; N 3.27.

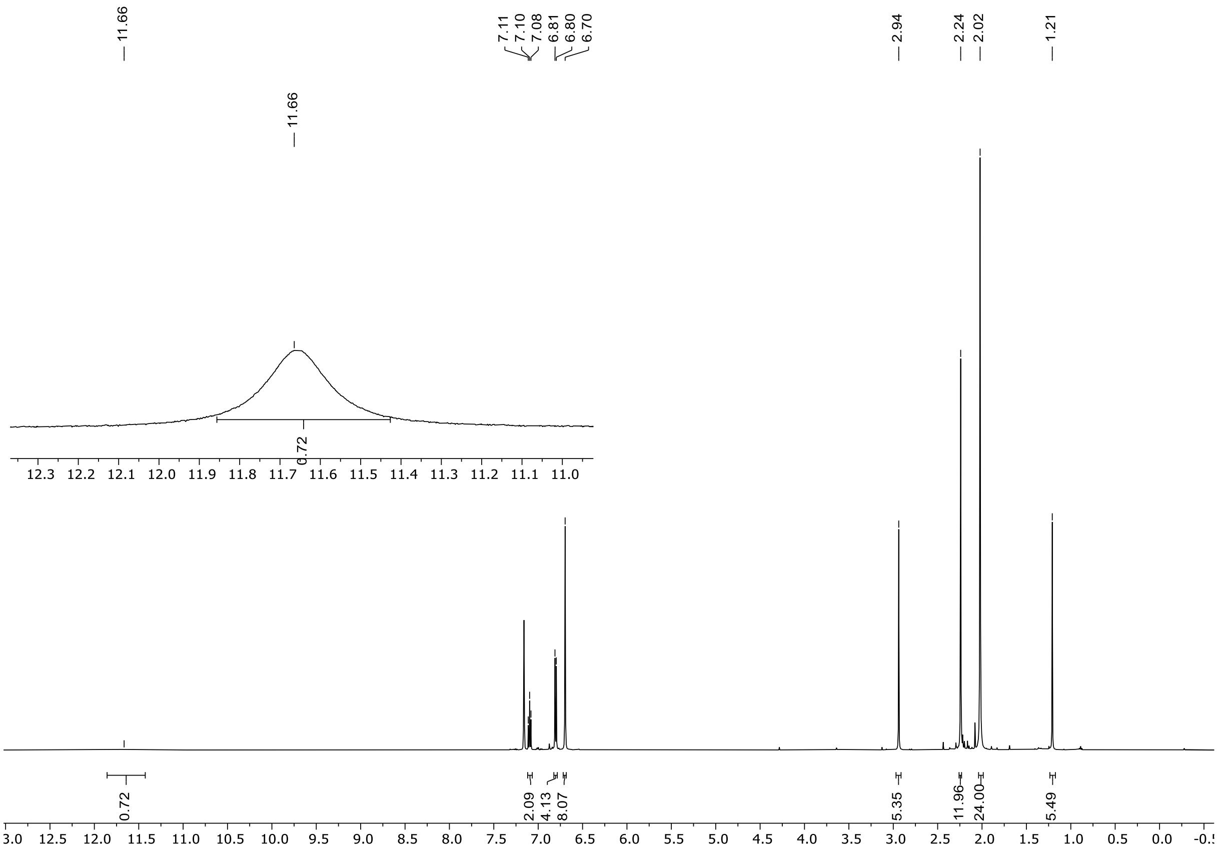

Figure S16. ^{1}H NMR spectrum of [$^{Mes}Ter_2BO$][$HIMe_4$] (**3a**) (400 MHz, C_6D_6, 298 K).

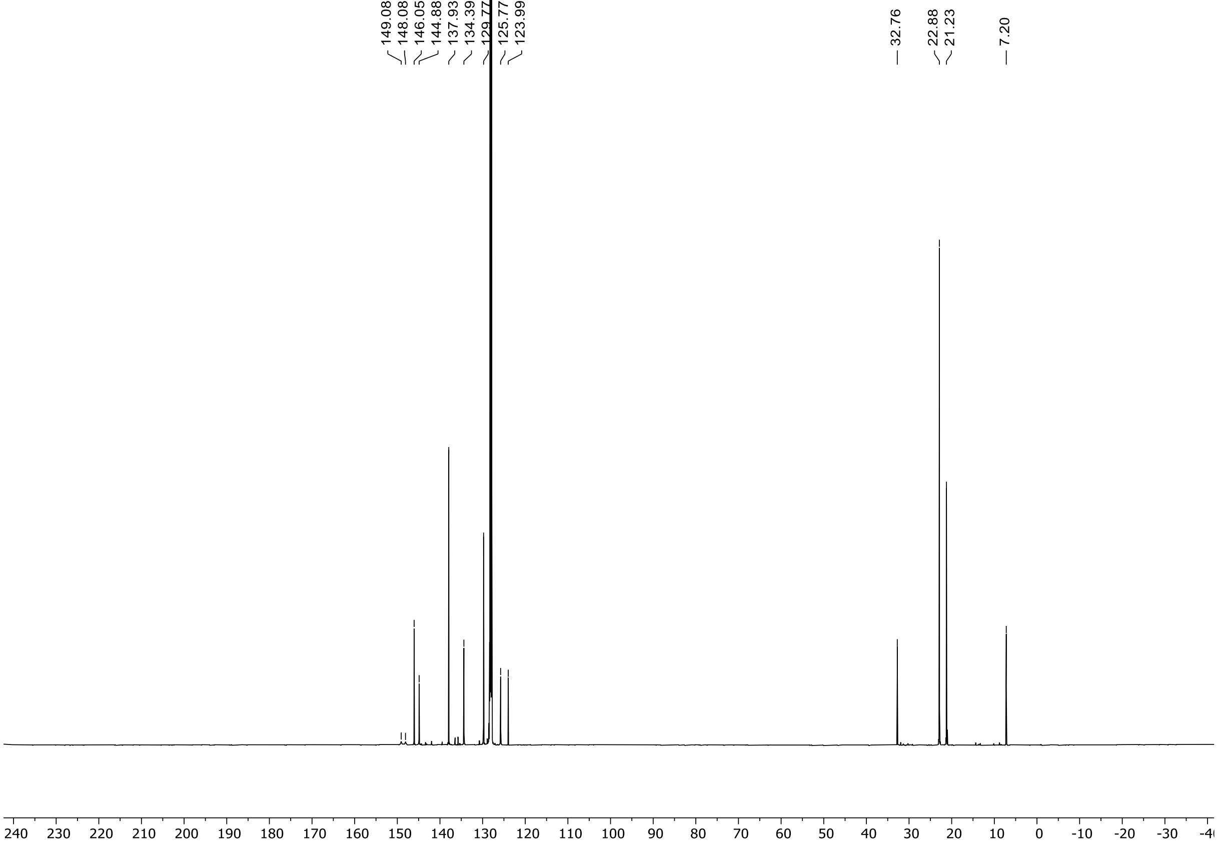

Figure S17. $^{13}C\{^{1}H\}$ NMR spectrum of [$^{Mes}Ter_2BO$][$HIMe_4$] (**3a**) (126 MHz, C_6D_6, 298 K).

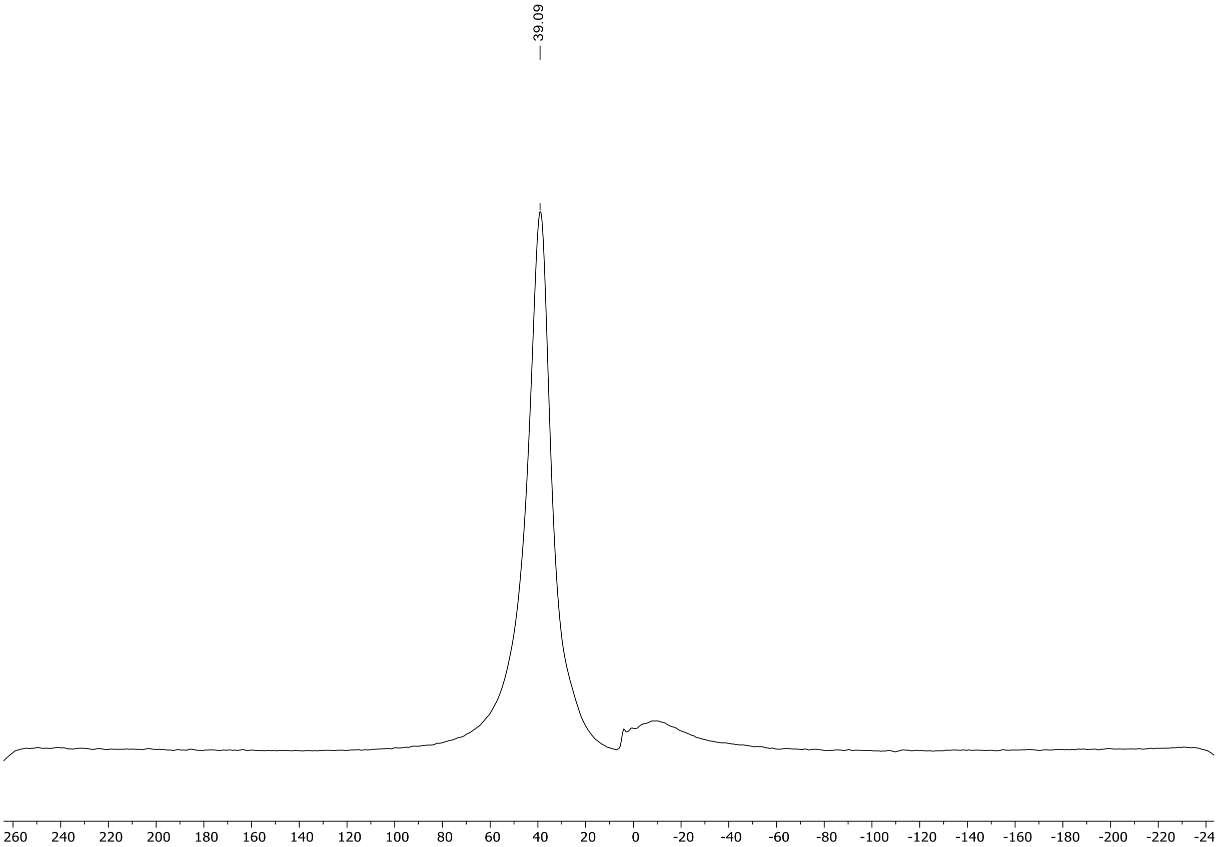

Figure S18. $^{11}B\{^{1}H\}$ NMR spectrum of [$^{Mes}Ter_2BO$][$HIMe_4$] (**3a**) (96 MHz, C_6D_6, 298 K).

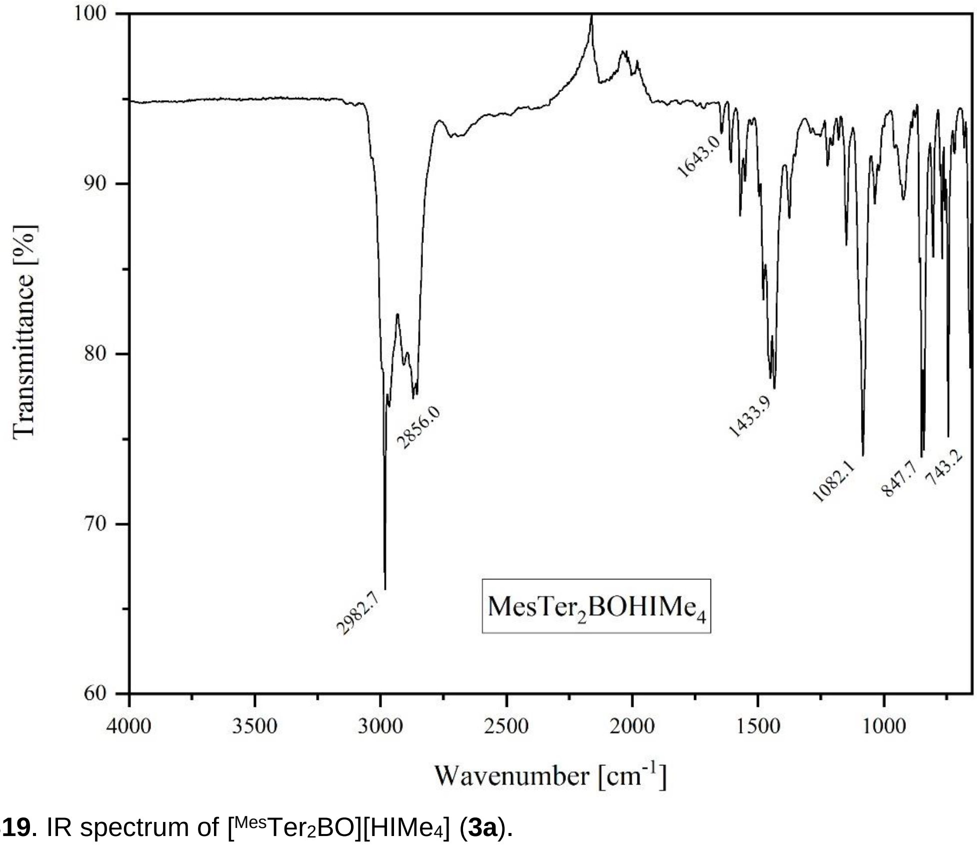

Figure S19. IR spectrum of [$^{Mes}Ter_2BO$][$HIMe_4$] (**3a**).
IR (ATR): $\tilde{v}$ [cm^{-1}] = 2983 (s), 2966 (s), 2907 (m), 2870 (s), 2856 (s), 1643 (w), 1607 (w), 1569 (m), 1551 (w), 1495 (w), 1478 (m), 1451 (s), 1434 (s), 1375 (m), 1222 (w), 1205 (w), 1179 (w), 1148 (m), 1082 (s), 1034 (w), 1018 (w), 956 (w), 921 (m), 848 (s), 839 (s), 804 (m), 767 (m), 756 (m), 743 (s), 718 (w), 680 (w), 657 (s).

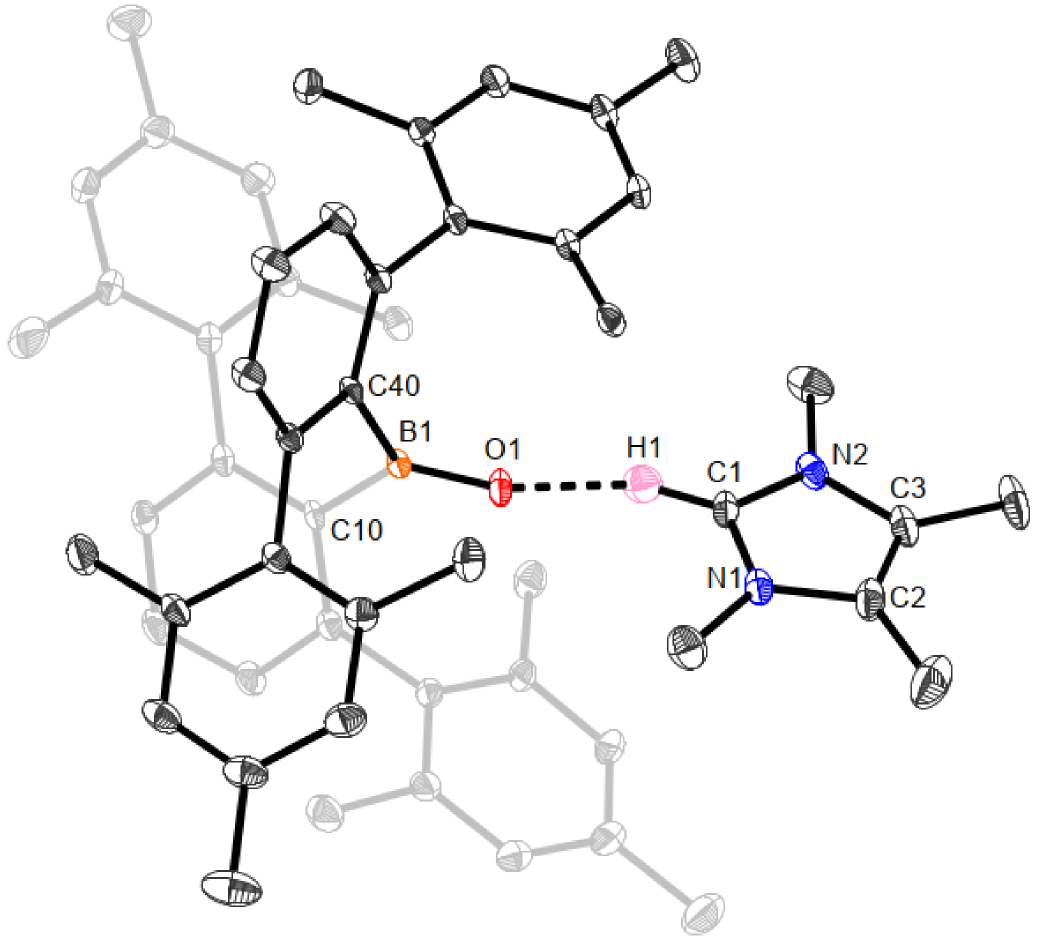

Figure S20. Molecular structure of [^{Mes}Ter$_2$BO][HIMe$_4$] (**3a**) in the crystal after Hirshfeld atom refinement (HAR). Anisotropic displacement parameters are drawn at the 50% probability level (lattice solvent have been omitted for clarity). Only the freely and anisotropically refined H1 atom is shown, while all other hydrogen atoms, although also refined freely and anisotropically, are omitted for clarity. Selected bond lengths (Å) and angles (deg): B1–O1 1.293(2), O1•••H1 1.832(26), B1–C10 1.637(2), B1–C40 1.639(2), O1–B1–C10 119.83(15), O1–B1–C40 120.59(15), C10–B1–C40 119.40(14), B1–O1–H1 159, C1–H1–O1 153, Σ∢B1 = 359.8°.

Synthesis of [(^{Mes}Ter)$_2$BO][HI^{i}Pr$_2$Me$_2$] (3b)

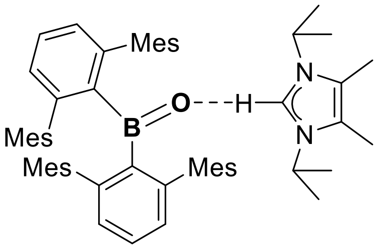

^{Mes}Ter$_2$BOH (0.030 g, 0.046 mmol) was dissolved in 0.3 mL of C_6D_6. Subsequently, I^{i}Pr$_2$Me$_2$ (0.008 g, 0.046 mmol) in 0.3 mL of C_6D_6 was added to the solution, and the reaction mixture was analysed by ^{1}H NMR spectroscopy, which revealed the clean formation of [^{Mes}Ter$_2$BO][HI^{i}Pr$_2$Me$_2$] (**3b**) (Figure S21). All volatile components were then removed under vacuum, yielding [^{Mes}Ter$_2$BO][HI^{i}Pr$_2$Me$_2$] (**3b**) as a slightly yellow solid. Crystals suitable for single crystal X-ray diffraction were obtained from a saturated benzene solution of **3b** at room temperature over the course of one week.

Yield: 0.035 g (0.042 mmol; 91%).
^{1}H NMR (400 MHz, C_6D_6, 298 K): δ = 1.43 (d, $^3J_{H,H}$ = 6.7 Hz, 12H, CH(C*H*$_3$)$_2$), 1.66 (s, 6H, NC$_q$CH$_3$), 1.85 (s, 24H, *o*-C*H*$_3$-C$_6$H$_3$), 2.24 (s, 12H, *p*-C*H*$_3$-C$_6$H$_3$), 3.97 (hept, $^3J_{H,H}$ = 6.6 Hz, 2H, C*H*(CH$_3$)$_2$), 6.66 (d, $^3J_{H,H}$ = 7.6 Hz, 4H, *m*-CH$_{Aryl}$-B), 6.77 (s, 8H, *m*-C*H*$_{Aryl}$-C$_6$H$_3$), 6.98 (t, $^3J_{H,H}$ = 7.6 Hz, 2H, *p*-CH$_{Aryl}$-B), 7.89 (s(br), 1H, HC$_{Im}$) ppm.
^{13}C{^{1}H} NMR (126 MHz, C_6D_6, 298 K): δ = 8.8 (NC$_q$$\underline{C}H_3$), 21.2 (*p*-$\underline{C}H_3$-C$_6H_3$), 22.3 (*o*-$\underline{C}H_3$-C$_6H_3$), 24.2 (CH($\underline{C}H_3$)$_2$), 49.1 ($\underline{C}$H(CH$_3$)$_2$), 122.0 (NC$_q$$\underline{C}H_3$), 127.7 (*p*-CH$_{Aryl}$-B)*, 128.9 (*m*-$\underline{C}H_{Aryl}$-C$_6H_3$), 130.5 (*m*-CH$_{Aryl}$-B), 136.2 (C$_{q,Aryl}$), 137.5 (C$_{q,Aryl}$), 141.4 (br, C$_{q,Aryl}$B), 142.0 (C$_{q,Aryl}$), 146.6 (C$_{q,Aryl}$) ppm.
* = overlap with C_6D_6 signal and assigned by ^{1}H/^{13}C-HSQC
^{11}B/^{11}B{^{1}H} NMR (96 MHz, C_6D_6, 298 K): δ = 46.2 ppm.
HR/MS: m/z calcd.: 653.3968 [$C_{48}H_{50}BO$]$^-$ measured (ESI, negative): m/z 653.3942.
HR/MS: m/z calcd.: 181.1699 [$C_{11}H_{21}N_2$]$^+$ measured (ESI, positive): m/z 181.1699.
EA: Anal. calcd. for $C_{59}H_{71}BN_2O$: C, 84.86; H, 8.57; N, 3.35; Found: C, 83.94; H, 8.32; N 3.45.

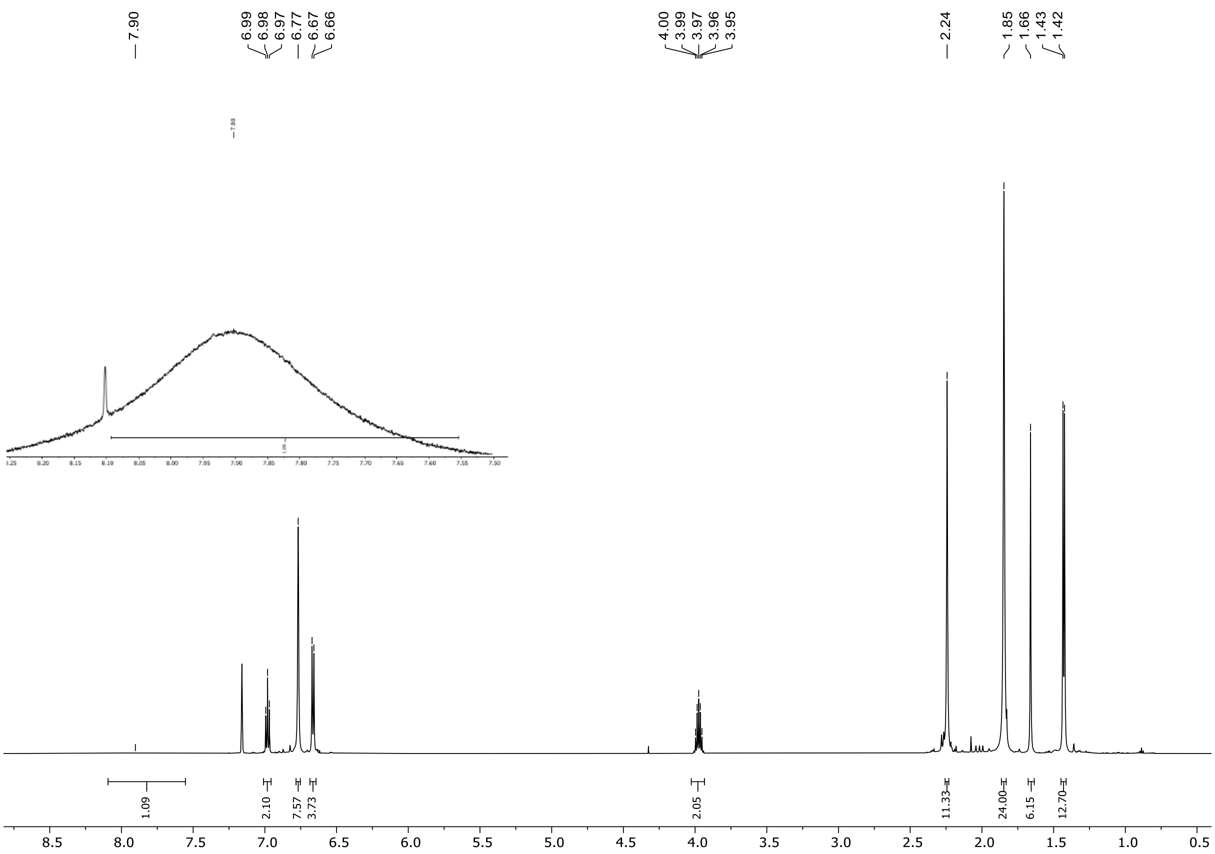

Figure S21. ^{1}H NMR spectrum of [(^{Mes}Ter)$_2$BO][HI^{i}Pr$_2$Me$_2$] (**3b**) (400 MHz, C_6D_6, 298 K).

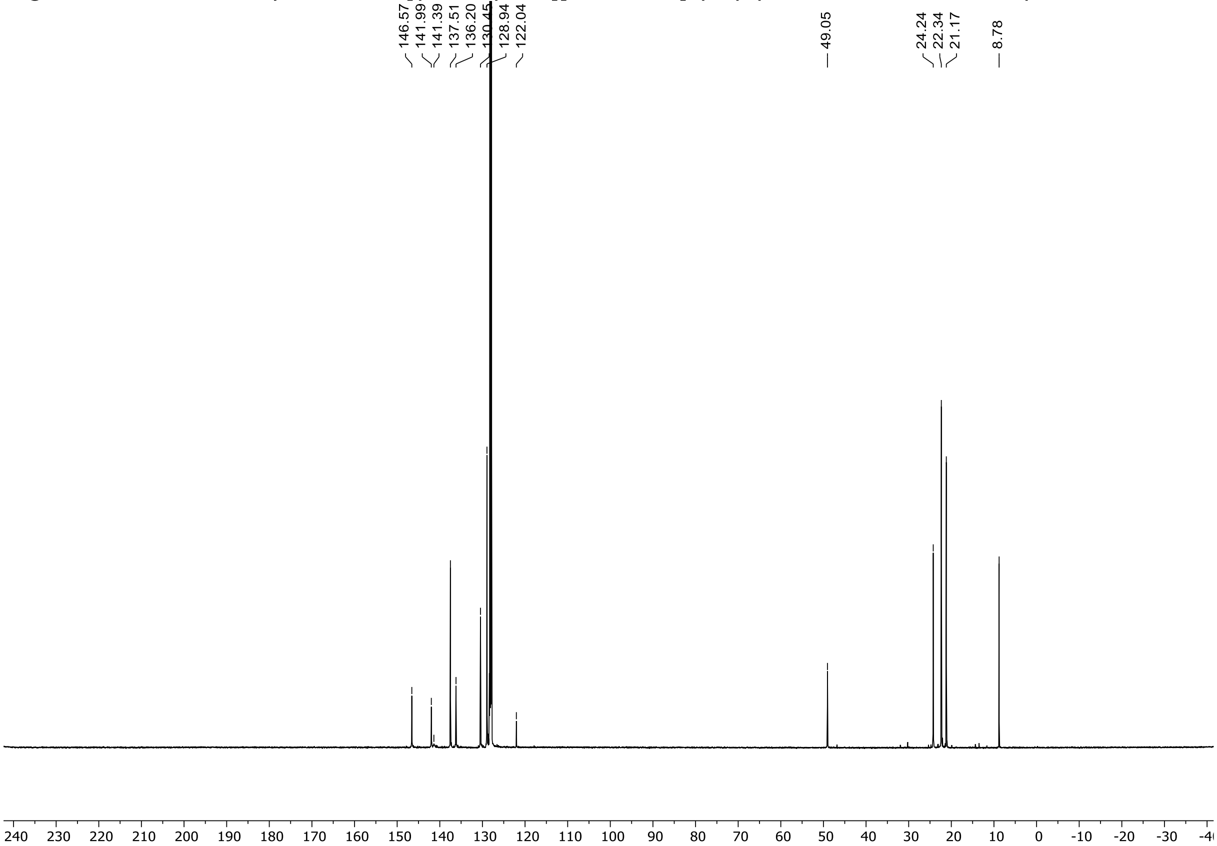

Figure S22. $^{13}C\{^{1}H\}$ NMR spectrum of [(^{Mes}Ter)$_2$BO][HI^{i}Pr$_2$Me$_2$] (**3b**) (126 MHz, C_6D_6, 298 K).

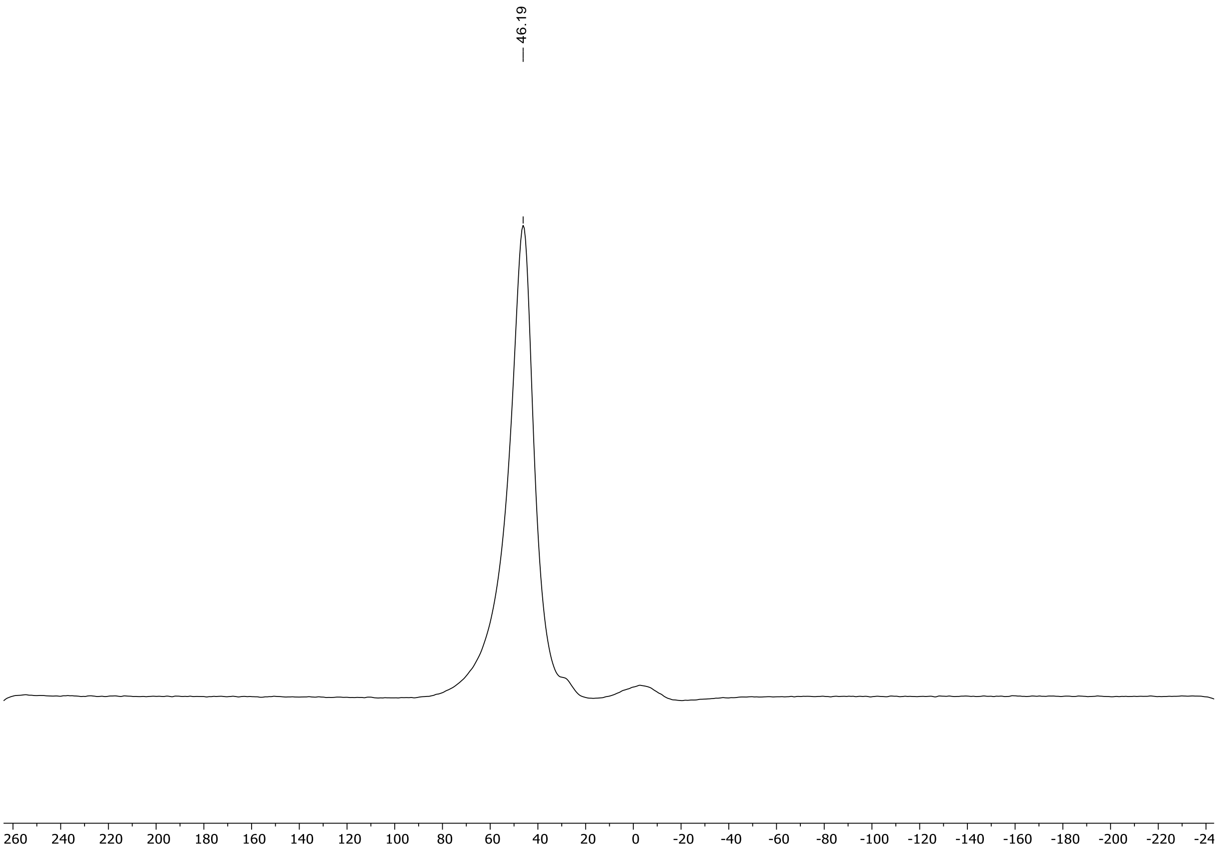

Figure S23. $^{11}B\{^{1}H\}$ NMR spectrum of $[(^{Mes}Ter)_2BO][HI^iPr_2Me_2]$ (**3b**) (96 MHz, C_6D_6, 298 K).

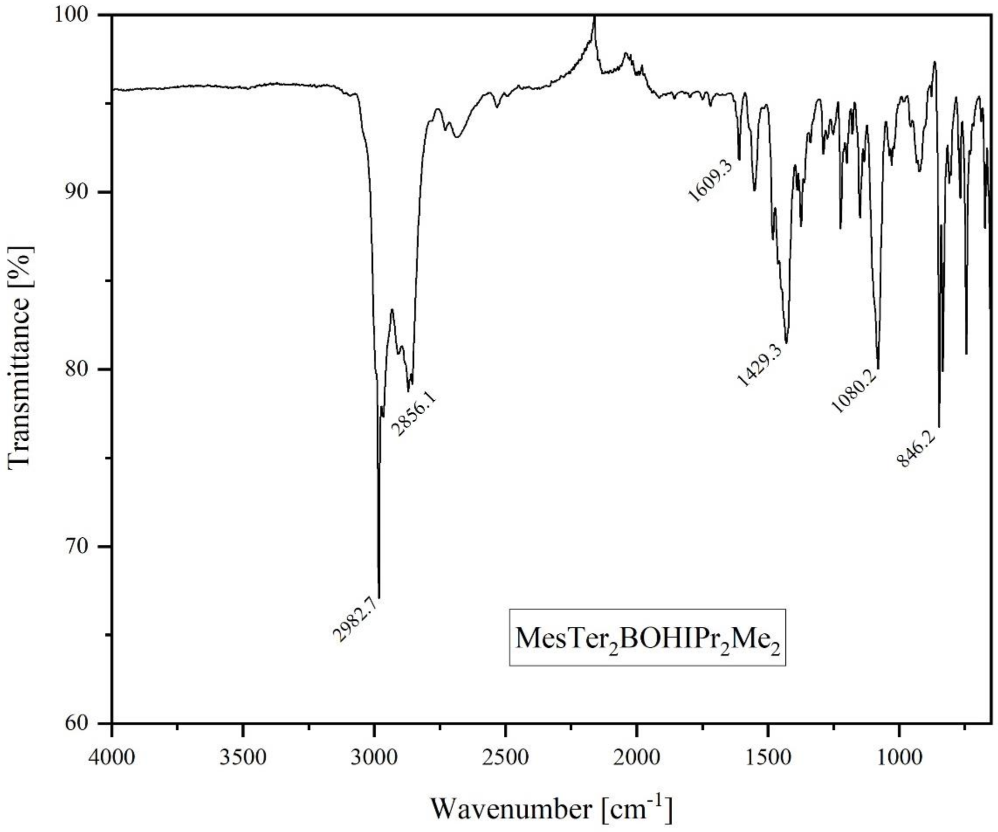

Figure S24. IR spectrum of [(^{Mes}Ter)$_2$BO][HI^{i}Pr$_2$Me$_2$] (**3b**).

IR (ATR): ṽ [cm^{-1}] = 2983 (s), 2967 (s), 2908 (m), 2871 (s), 2856 (s), 1609 (w), 1551 (m), 1481 (m), 1429 (s), 1391 (w), 1374 (m), 1338 (w), 1288 (w), 1273 (w), 1251 (w), 1222 (m), 1199 (w), 1178 (w), 1148 (m), 1080 (s), 1027 (w), 956 (w), 921 (m), 846 (s), 833 (s), 808 (w), 767 (m), 744 (s), 686 (w), 672 (w), 653 (m).

Synthesis of [^{Mes}Ter$_2$BOK]$_2$ (4)

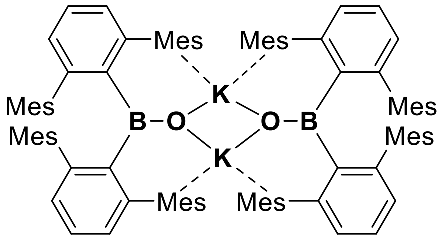

A) ^{Mes}Ter$_2$BOH (0.030 g, 0.046 mmol) was dissolved in 0.3 mL of C_6D_6. K{N(SiMe$_3$)$_2$} (0.009 g, 0.046 mmol) in 0.3 mL of C_6D_6 was added to the solution, and the reaction mixture was subsequently analysed by ^{1}H NMR spectroscopy, revealing the clean formation of [^{Mes}TerBOK]$_2$ (**4**) and HN(SiMe$_3$)$_2$ (δ^1H = 0.10 ppm). All volatile components were then removed under vacuum, yielding [^{Mes}TerBOK]$_2$ (**4**) as a colourless solid. Crystals suitable for single-crystal X-ray diffraction were obtained from a saturated DCM solution of **4** at -30 °C.
B) ^{Mes}TerBOH (0.030 g, 0.046 mmol) was dissolved in 0.3 mL of THF-d_8. K{N(SiMe$_3$)$_2$} (0.009 g, 0.046 mmol) in 0.3 mL of THF-d_8 was added to the solution, and the reaction mixture was subsequently analysed by ^{1}H NMR spectroscopy, revealing clean formation of [^{Mes}TerBOK]$_2$ (**4**). All volatile components were then removed under vacuum, yielding **4** as a colourless solid. All attempts to crystallize **4** from THF solutions (including slow evaporation and slow diffusion of aliphatic hydrocarbons into saturated solutions of **4** in THF) resulted in crystals with the same unit cell as those obtained from procedure **A)**.

Yield: 0.029 g (0.021 mmol; 91% (**A**)).
^{1}H NMR (400 MHz, C_6D_6, 298 K): δ = 1.36 (s, 12H, CH$_3$), 2.00 (s, 12H, CH$_3$), 2.02 (s, 12H, CH$_3$), 2.04 (s, 12H, CH$_3$), 2.27 (s, 12H, CH$_3$), 2.28 (s, 12H, CH$_3$), 6.53-6.56 (m, 4H, CH$_{Aryl}$), 6.65 (m, 4H, CH$_{Aryl}$), 6.70-6.72 (m, 8H, CH$_{Aryl}$), 6.80-6.82 (m, 8H, CH$_{Aryl}$), 7.09 (t, $^3J_{H,H}$ = 7.5 Hz, 4H, CH$_{Aryl}$) ppm.
^{1}H NMR (400 MHz, THF-d_8, 298 K): δ = 1.69 ppm (s, 24H, *o*-C*H*$_3$-C$_6$H$_3$), 2.23 (s, 12H, *p*-C*H*$_3$-C$_6$H$_3$), 6.31 (d, $^3J_{H,H}$ = 7.5 Hz, 4H, *m*-CH$_{Aryl}$-B), 6.62 (s, 8H, *m*-C*H*$_{Aryl}$-C$_6$H$_3$), 6.79 (t, $^3J_{H,H}$ = 7.6 Hz, 2H, *p*-CH$_{Aryl}$-B) ppm.
^{13}C{^{1}H} NMR (126 MHz, C_6D_6, 298 K): δ = 19.9 (CH$_3$), 21.0 (CH$_3$), 21.1 (CH$_3$), 23.2 (CH$_3$), 24.8 (CH$_3$), 25.4 (CH$_3$), 126.3 (CH$_{Aryl}$), 126.6 (CH$_{Aryl}$), 128.6 (CH$_{Aryl}$), 128.86 (CH$_{Aryl}$), 128.93 (CH$_{Aryl}$), 130.2 (CH$_{Aryl}$), 131.0 (CH$_{Aryl}$), 135.0 (C$_{q,Aryl}$), 136.0 (C$_{q,Aryl}$), 137.58 (C$_{q,Aryl}$), 137.62 (C$_{q,Aryl}$), 138.9 (C$_{q,Aryl}$), 140.6 (C$_{q,Aryl}$), 144.8 (C$_{q,Aryl}$), 145.2 (C$_{q,Aryl}$), 146.2 (C$_{q,Aryl}$), 147.9 (C$_{q,Aryl}$), 151.9 (C$_{q,Aryl}$B) ppm.
^{13}C{^{1}H} NMR (126 MHz, THF-d_8, 298 K): δ = 21.0 (*p*-$\underline{C}$H$_3$-C$_6$H$_3$), 22.7 (*o*-$\underline{C}$H$_3$-C$_6$H$_3$), 124.6 (*p*-CH$_{Aryl}$-B), 128.1 (*m*-C*H*$_{Aryl}$-C$_6$H$_3$), 129.3 (*m*-CH$_{Aryl}$-B), 134.1 (C$_{q,Aryl}$), 137.6 (C$_{q,Aryl}$), 145.9 (C$_{q,Aryl}$), 146.4 (C$_{q,Aryl}$), 150.9 (br, C$_{q,Aryl}$B).
^{11}B/^{11}B{^{1}H} NMR (96 MHz, C_6D_6, 298 K): δ = 40.6 ppm.
^{11}B/^{11}B{^{1}H} NMR (96 MHz, THF-d_8, 298 K): δ = 35.8 ppm.
HR/MS: m/z calcd.: 693.3673 [0.5 M + H$^+$]; measured (ESI, positive): m/z 693.3664.
EA: Anal. calcd. for $C_{96}H_{100}B_2K_2O_2$: C, 83.21; H, 7.27; Found: C, 82.40; H, 7.27.

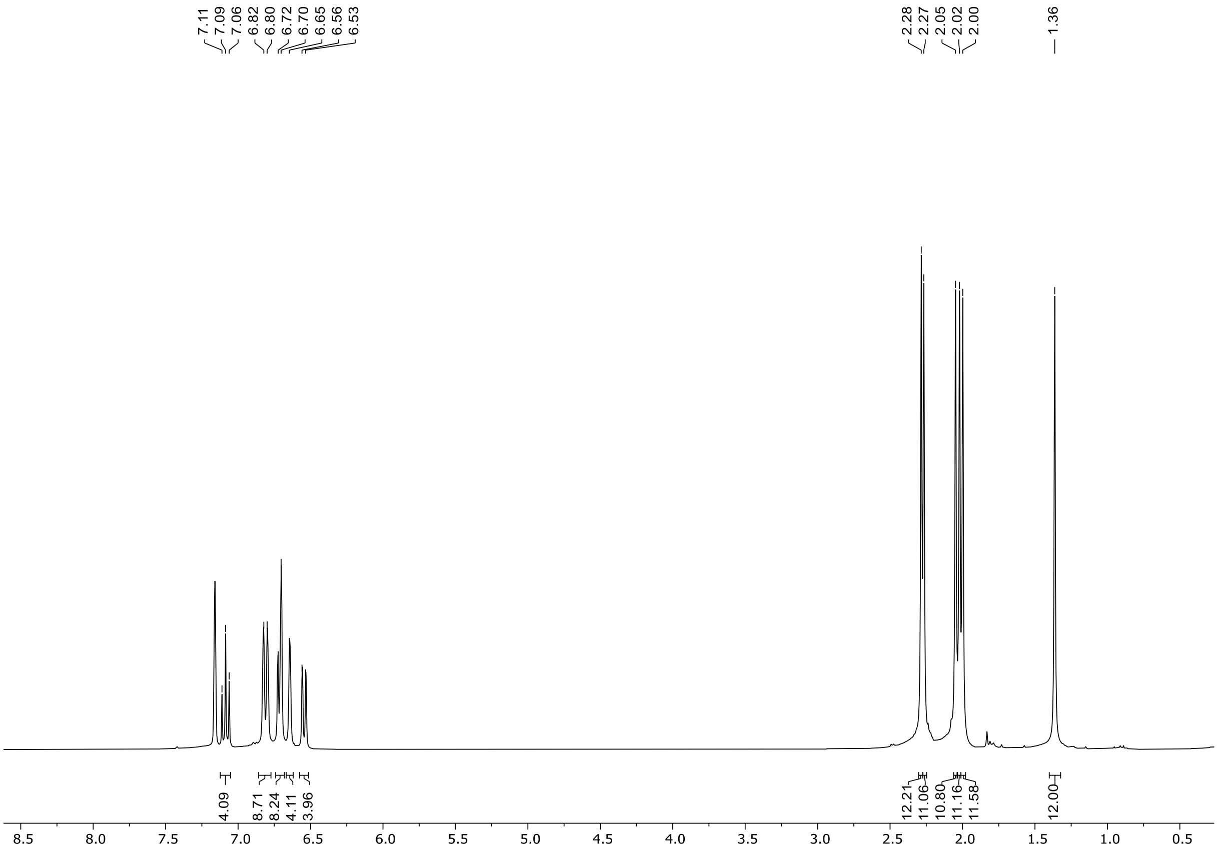

Figure S25. ^{1}H NMR spectrum of $[^{Mes}Ter_2BOK]_2$ (**4**) (400 MHz, C_6D_6, 298 K).

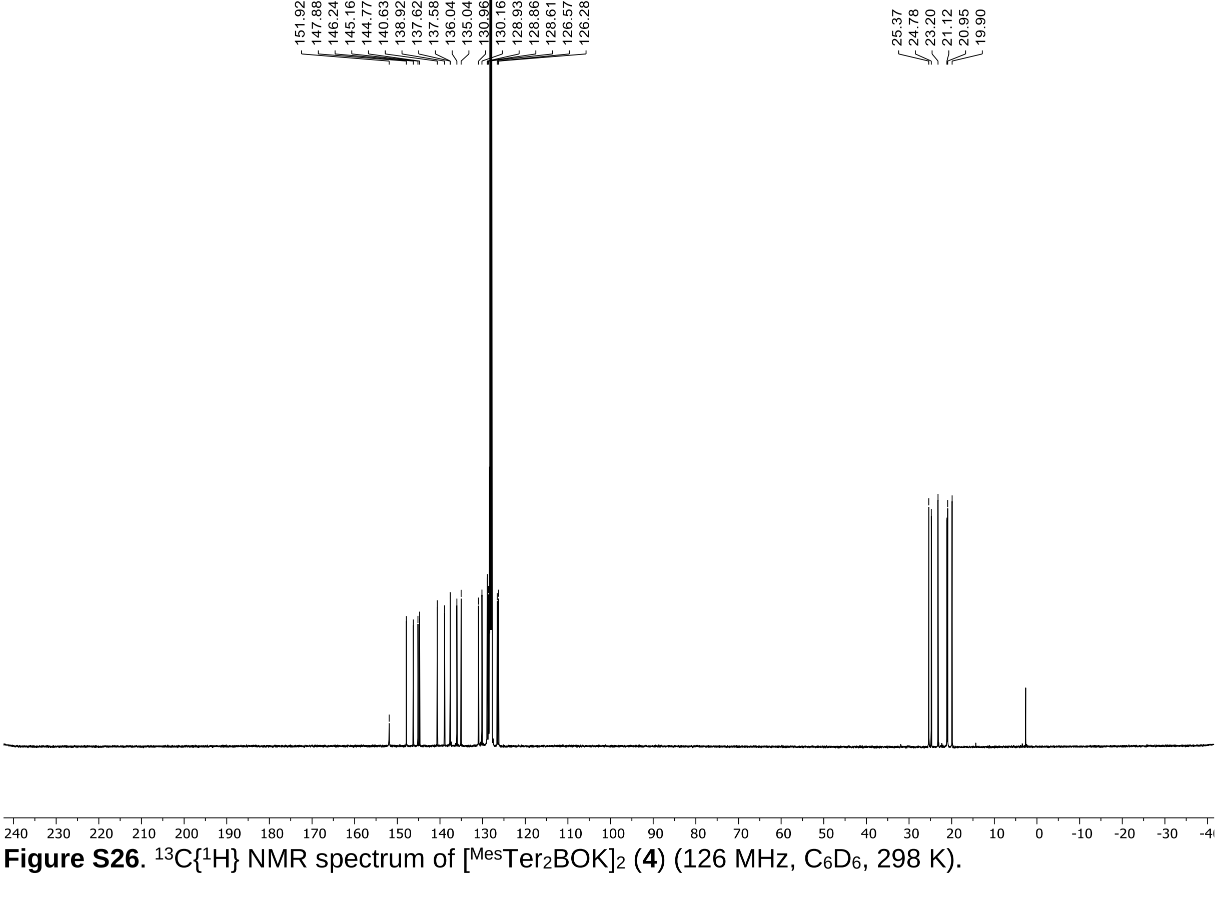

Figure S26. $^{13}C\{^{1}H\}$ NMR spectrum of $[^{Mes}Ter_2BOK]_2$ (**4**) (126 MHz, C_6D_6, 298 K).

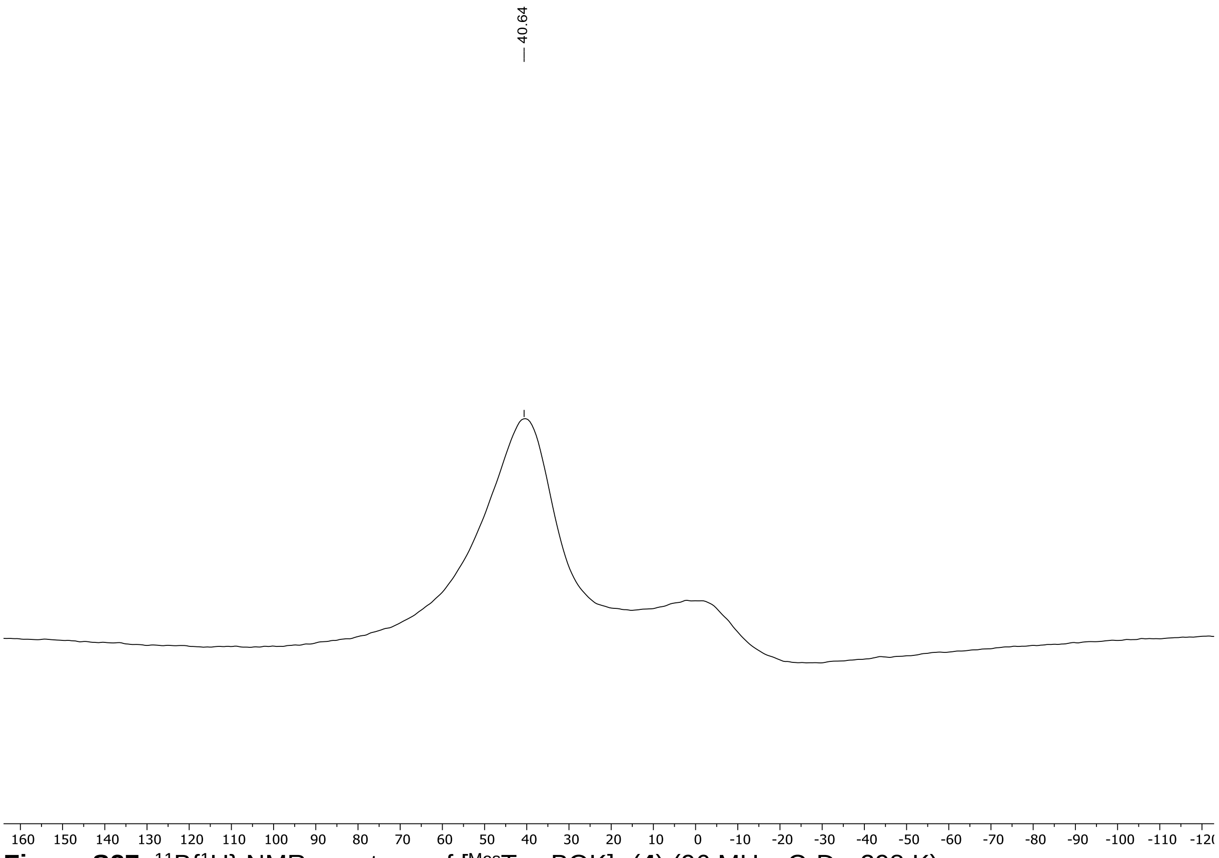

Figure S27. $^{11}B\{^{1}H\}$ NMR spectrum of $[^{Mes}Ter_2BOK]_2$ (**4**) (96 MHz, C_6D_6, 298 K).

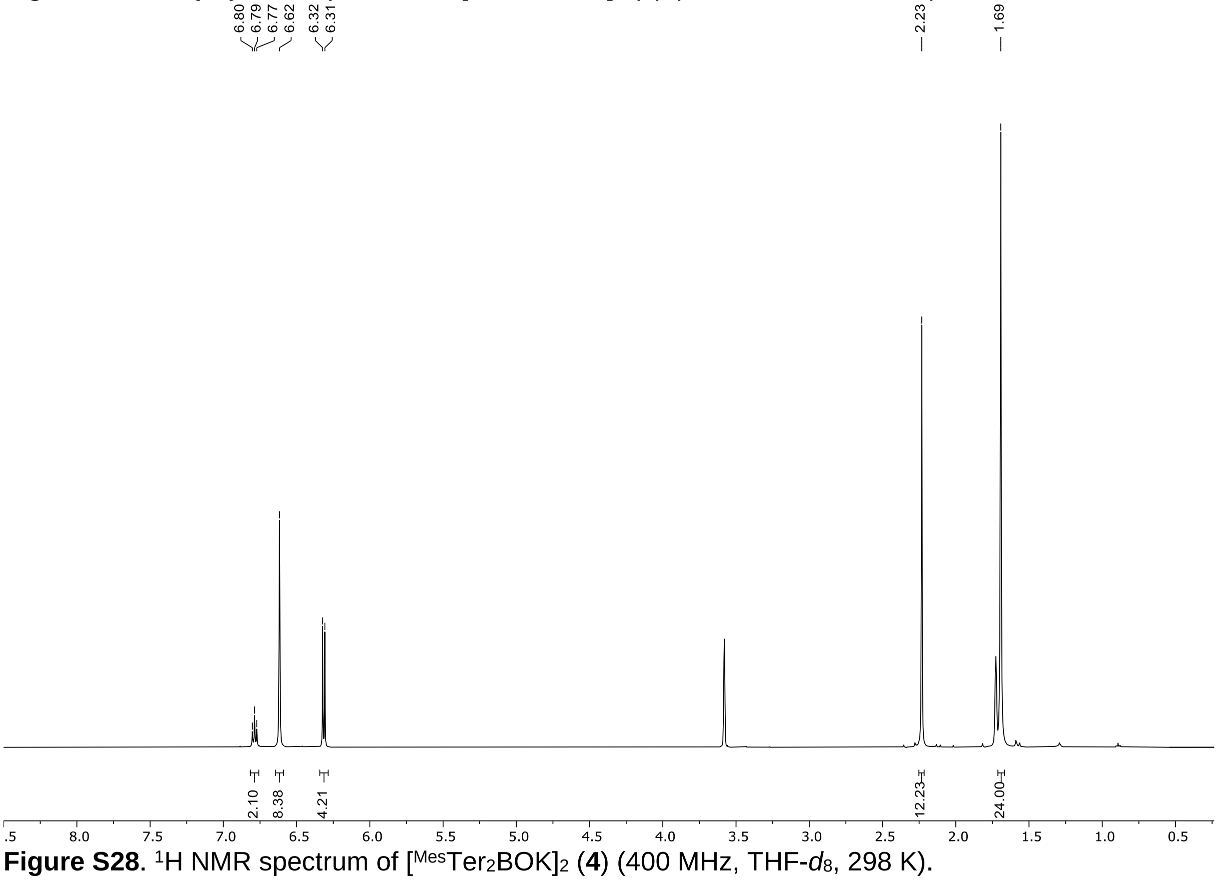

Figure S28. ^{1}H NMR spectrum of $[^{Mes}Ter_2BOK]_2$ (**4**) (400 MHz, THF-d_8, 298 K).

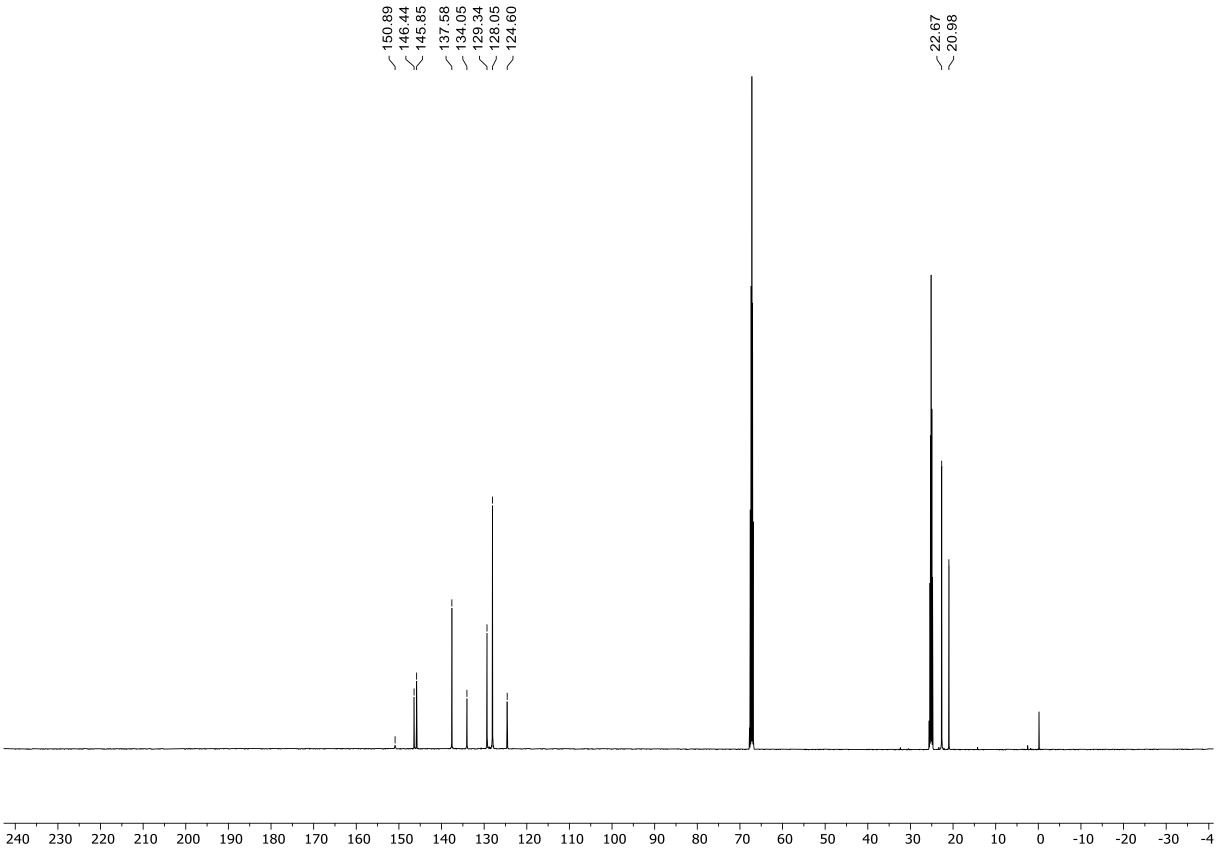

Figure S29. $^{13}C\{^{1}H\}$ NMR spectrum of $[^{Mes}Ter_2BOK]_2$ (**4**) (126 MHz, THF-d_8, 298 K).

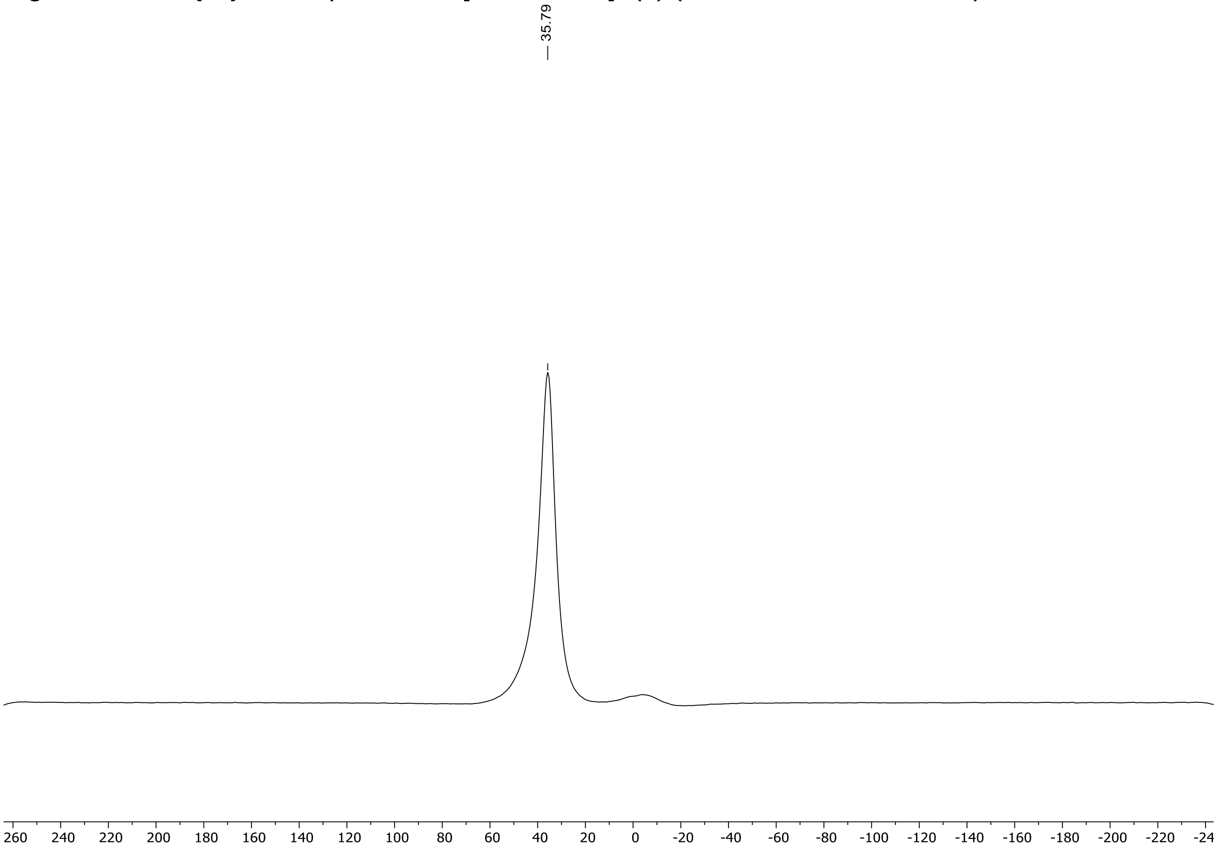

Figure S30. $^{11}B\{^{1}H\}$ NMR spectrum of $[^{Mes}Ter_2BOK]_2$ (**4**) (96 MHz, THF-d_8, 298 K).

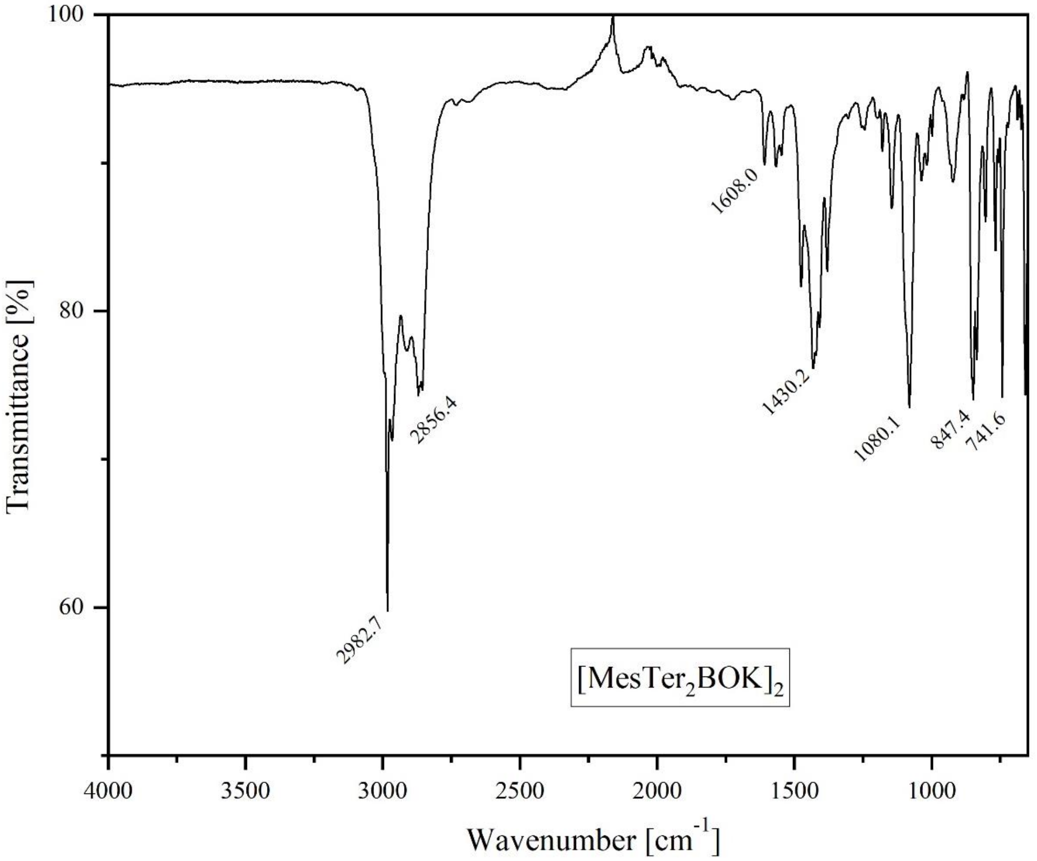

Figure S31. IR spectrum of [$^{Mes}Ter_2BOK]_2$ (**4**).
IR (ATR): ṽ [cm^{-1}] = 2983 (s), 2966 (s), 2912 (m), 2870 (s), 2856 (s), 1608 (m), 1566 (m), 1547 (w), 1475 (m), 1430 (s), 1408 (m), 1380 (m), 1178 (w), 1144 (m), 1080 (s), 1036 (m), 1017 (m), 998 (w), 921 (m), 847 (s), 835 (s), 803 (m), 766 (m), 742 (s), 657 (s).

Synthesis of [^{Mes}Ter$_2$BO][K(18c6)] (5a)

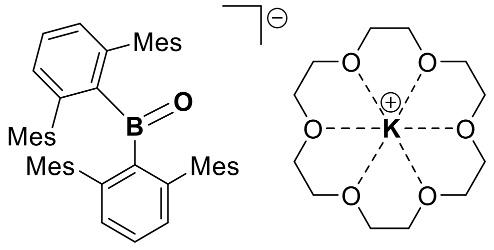

^{Mes}Ter$_2$BOH (**2**) (0.030 g, 0.046 mmol) was dissolved in 0.5 mL of benzene, followed by the addition of K{N(SiMe$_3$)$_2$} (0.009 g, 0.046 mmol) in 0.5 mL of benzene. The reaction mixture was stirred for 15 minutes at room temperature to facilitate the *in situ* generation of [^{Mes}Ter$_2$BOK]$_2$ (**4**). All volatile components were then removed under vacuum. The resulting solid was dissolved in 0.5 mL of C_6D_6 or THF-d_8, transferred to a J-Young NMR tube, and 18c6 (0.012 g, 0.046 mmol) was added. Multinuclear NMR spectroscopy revealed the clean formation of [^{Mes}Ter$_2$BO][K(18c6)] (**5a**). NMR data was collected at that point. All volatile components were subsequently removed under vacuum to give **5a** as a colourless solid.
Despite multiple attempts, crystals of **5a** suitable for single crystal X-ray diffraction could not be obtained. The attempts included slow evaporation of saturated and unsaturated solutions, vapor diffusion, slow cooling of saturated solutions, and the use of the following solvents and their combinations: benzene, toluene, mesitylene, THF, n-hexane, n-pentane, dichloromethane, and 1,2-difluorobenzene.

Yield: 0.038 g (0.040 mmol; 87%).
^{1}H NMR (400 MHz, C_6D_6, 298 K): δ = 2.18 (s, 24H, *o*-C*H*$_3$-C$_6$H$_3$), 2.41 (s, 12H, *p*-C*H*$_3$-C$_6$H$_3$), 3.08 (s, 24H, CH$_2$), 6.77 (d, $^3J_{H,H}$ = 7.5 Hz, 4H, *m*-CH$_{Aryl}$-B), 7.00 (s, 8H, *m*-C*H*$_{Aryl}$-C$_6$H$_3$), 7.04 (t, $^3J_{H,H}$ = 7.5 Hz, 2H, *p*-CH$_{Aryl}$-B) ppm.
^{1}H NMR (400 MHz, THF-d_8, 298 K): δ = 1.70 ppm (s, 24H, *o*-C*H*$_3$-C$_6$H$_3$), 2.23 (s, 12H, *p*-C*H*$_3$-C$_6$H$_3$), 3.56 (s, 24H, CH$_2$), 6.28 (d, $^3J_{H,H}$ = 7.5 Hz, 4H, *m*-CH$_{Aryl}$-B), 6.62 (s, 8H, *m*-C*H*$_{Aryl}$-C$_6$H$_3$), 6.74 (t, $^3J_{H,H}$ = 7.5 Hz, 2H, *p*-CH$_{Aryl}$-B) ppm.
^{13}C{^{1}H} NMR (126 MHz, C_6D_6, 298 K): δ = 21.4 (*p*-$\underline{C}$H$_3$-C$_6$H$_3$), 23.1 (*o*-$\underline{C}$H$_3$-C$_6$H$_3$), 69.8 (CH$_2$), 124.3 (*p*-CH$_{Aryl}$-B), 128.2 (*m*-C*H*$_{Aryl}$-C$_6$H$_3$)*, 129.6 (*m*-CH$_{Aryl}$-B), 134.4 (C$_{q,Aryl}$), 138.0 (C$_{q,Aryl}$), 145.9 (C$_{q,Aryl}$), 146.0 (C$_{q,Aryl}$), 152.4 (C$_{q,Aryl}$B) ppm.
* = overlap with C_6D_6 signal
^{13}C{^{1}H} NMR (126 MHz, THF-d_8, 298 K): δ = 21.2 (*p*-$\underline{C}$H$_3$-C$_6$H$_3$), 22.8 (*o*-$\underline{C}$H$_3$-C$_6$H$_3$), 71.1 (CH$_2$), 123.9 (*p*-CH$_{Aryl}$-B), 128.1 (*m*-C*H*$_{Aryl}$-C$_6$H$_3$), 129.3 (*m*-CH$_{Aryl}$-B), 134.0 (C$_{q,Aryl}$), 138.0 (C$_{q,Aryl}$), 145.92 (C$_{q,Aryl}$), 145.96 (C$_{q,Aryl}$), 152.6 (br, C$_{q,Aryl}$B).
^{11}B/^{11}B{^{1}H} NMR (96 MHz, C_6D_6, 298 K): δ = 38.7 ppm.
^{11}B/^{11}B{^{1}H} NMR (96 MHz, THF-d_8, 298 K): δ = 36.0 ppm.
HR/MS: m/z calcd.: 653.3968 [$C_{48}H_{50}BO$]$^-$ measured (ESI, negative): m/z 653.3956.
HR/MS: m/z calcd.: 303.1204 [$C_{12}H_{24}O_6K$]$^+$ measured (ESI, positive): m/z 303.1210.
EA: Anal. calcd. for $C_{60}H_{74}BKO_7$: C, 75.29; H, 7.79; Found: C, 74.99; H, 7.62.

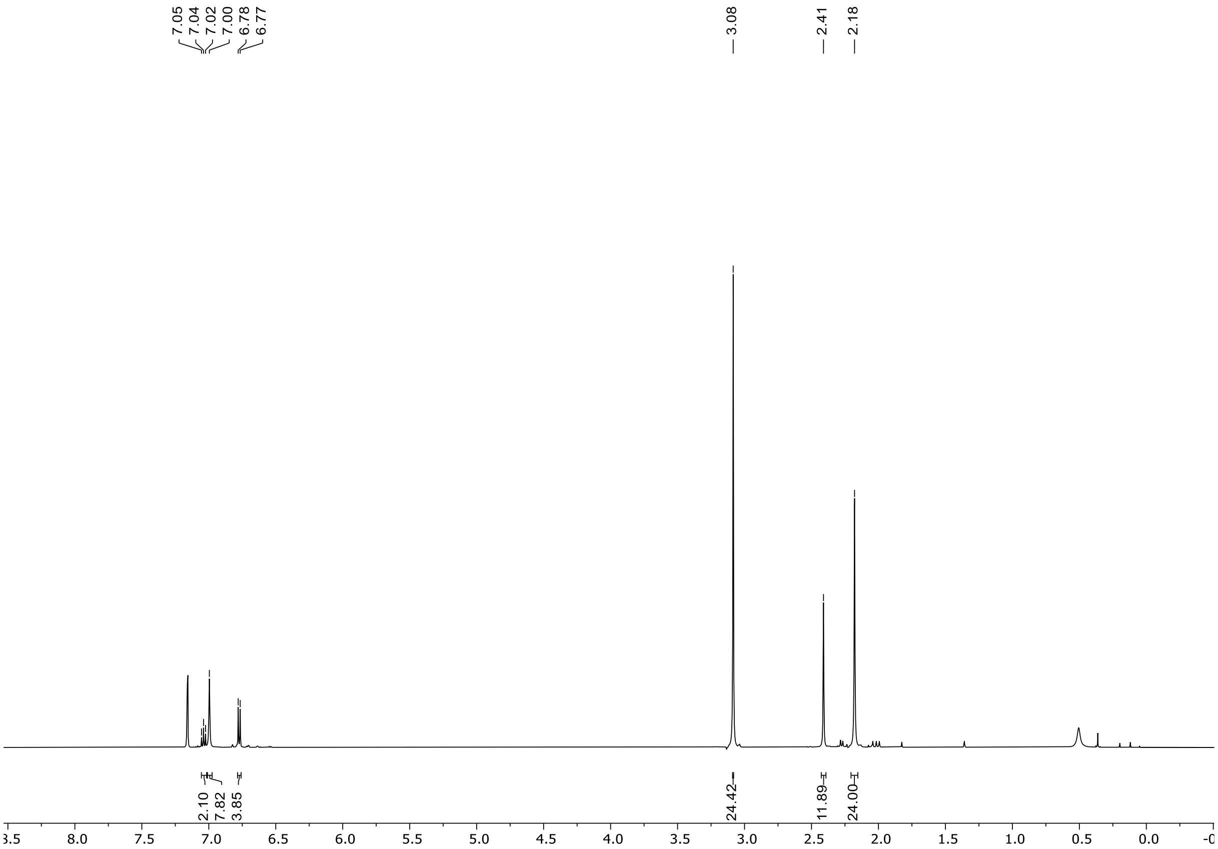

Figure S32. ^{1}H NMR spectrum of [^{Mes}Ter$_2$BO][K(18c6)] (**5a**) (400 MHz, C_6D_6, 298 K); 0.50 ppm: K{N(SiMe$_3$)$_2$}.

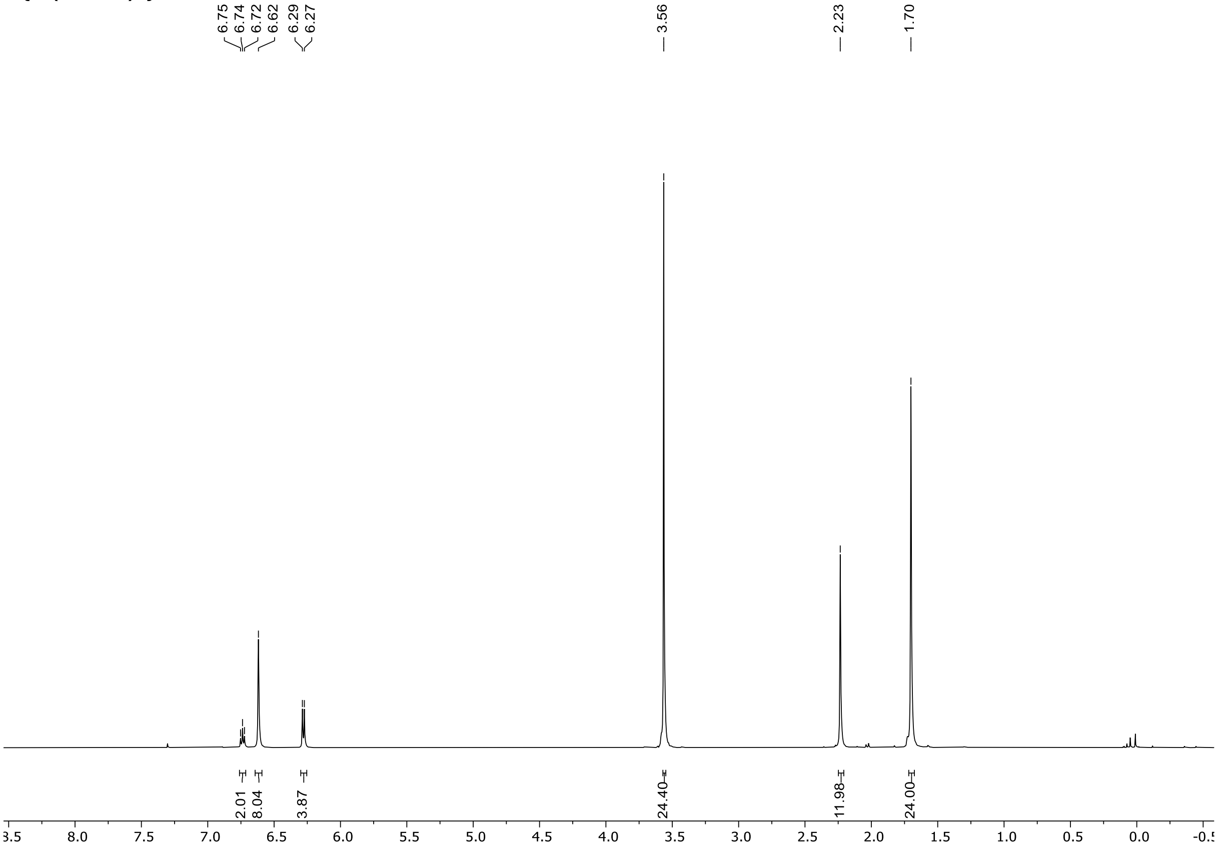

Figure S33. ^{1}H NMR spectrum of [^{Mes}Ter$_2$BO][K(18c6)] (**5a**) (400 MHz, THF-d_8, 298 K).

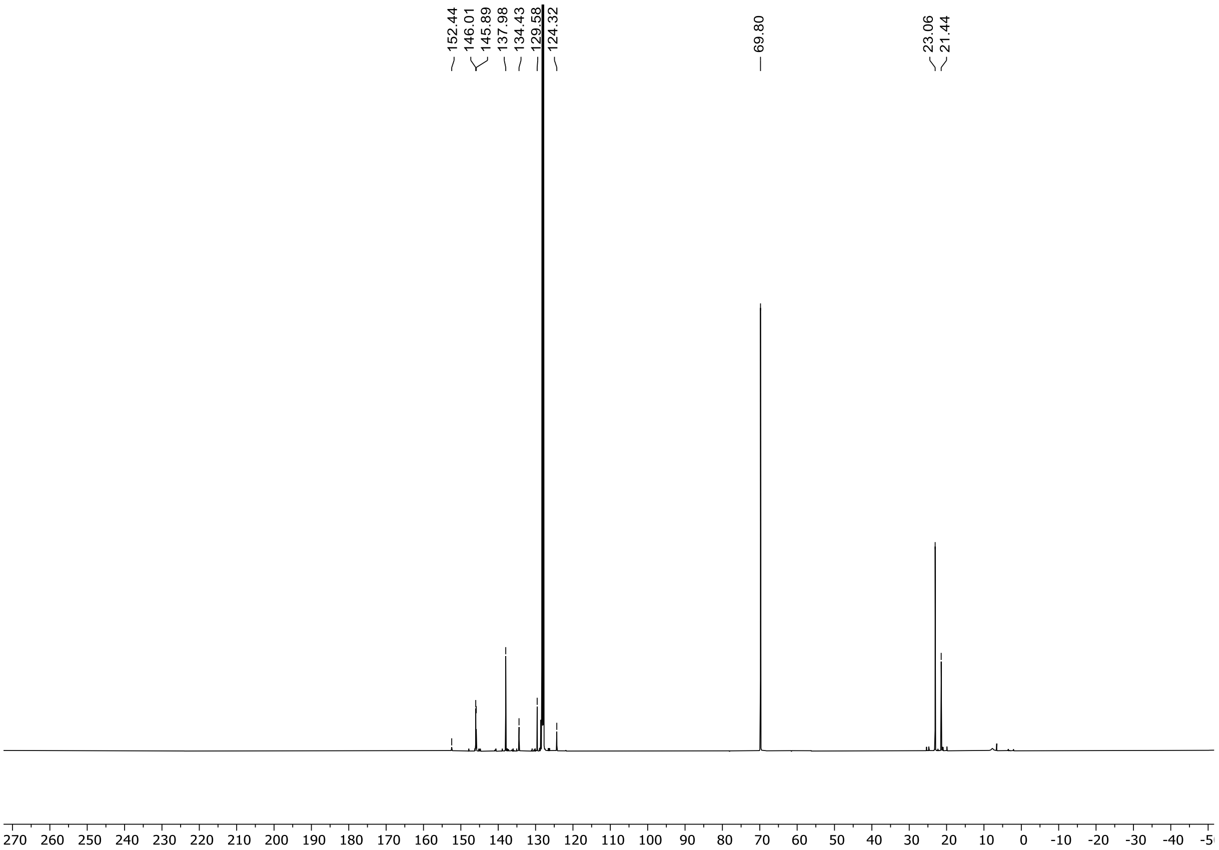

Figure S34. $^{13}C\{^1H\}$ NMR spectrum of [$^{Mes}Ter_2BO$][K(18c6)] (**5a**) (126 MHz, C_6D_6, 298 K).

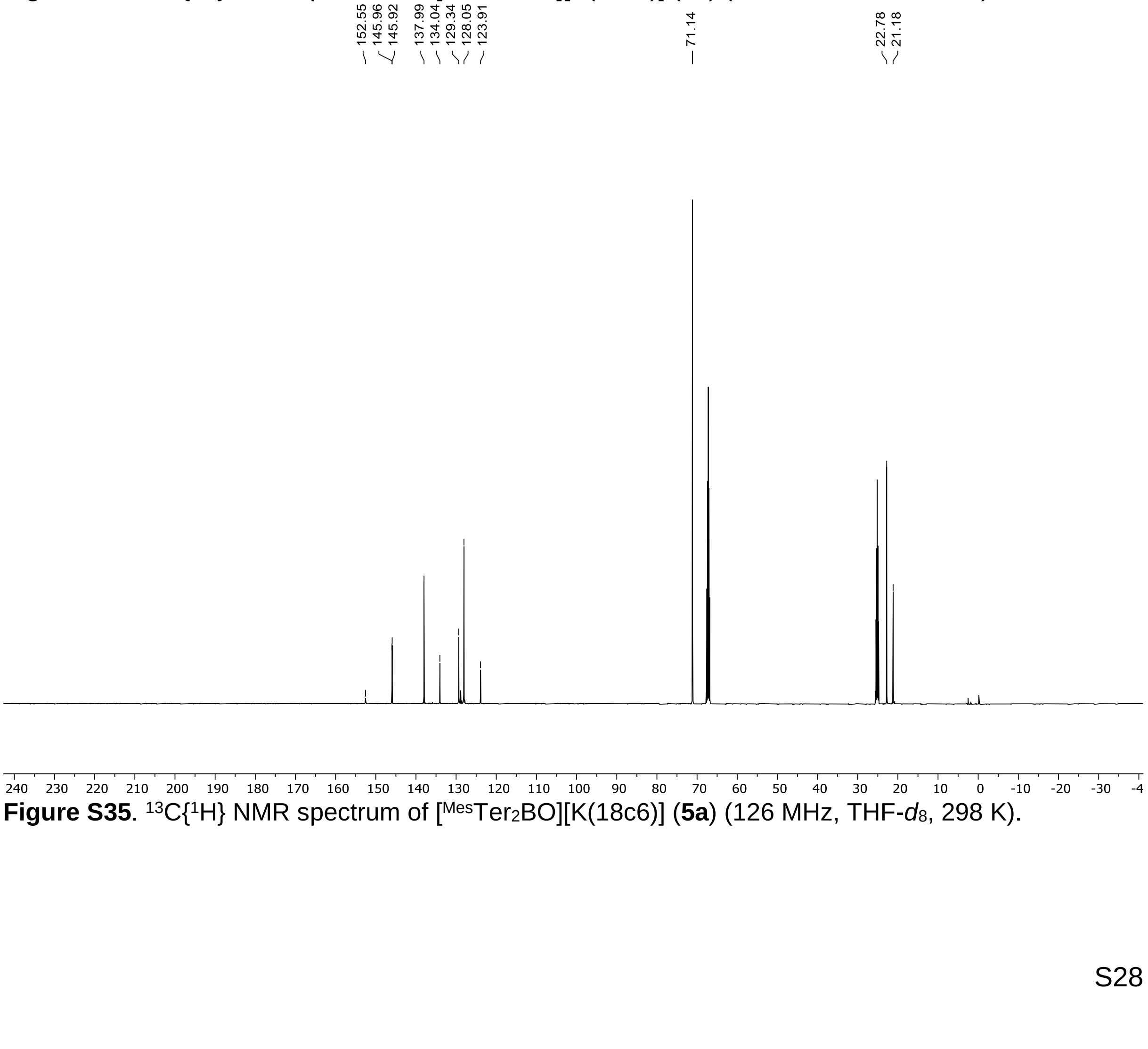

Figure S35. $^{13}C\{^1H\}$ NMR spectrum of [$^{Mes}Ter_2BO$][K(18c6)] (**5a**) (126 MHz, THF-d_8, 298 K).

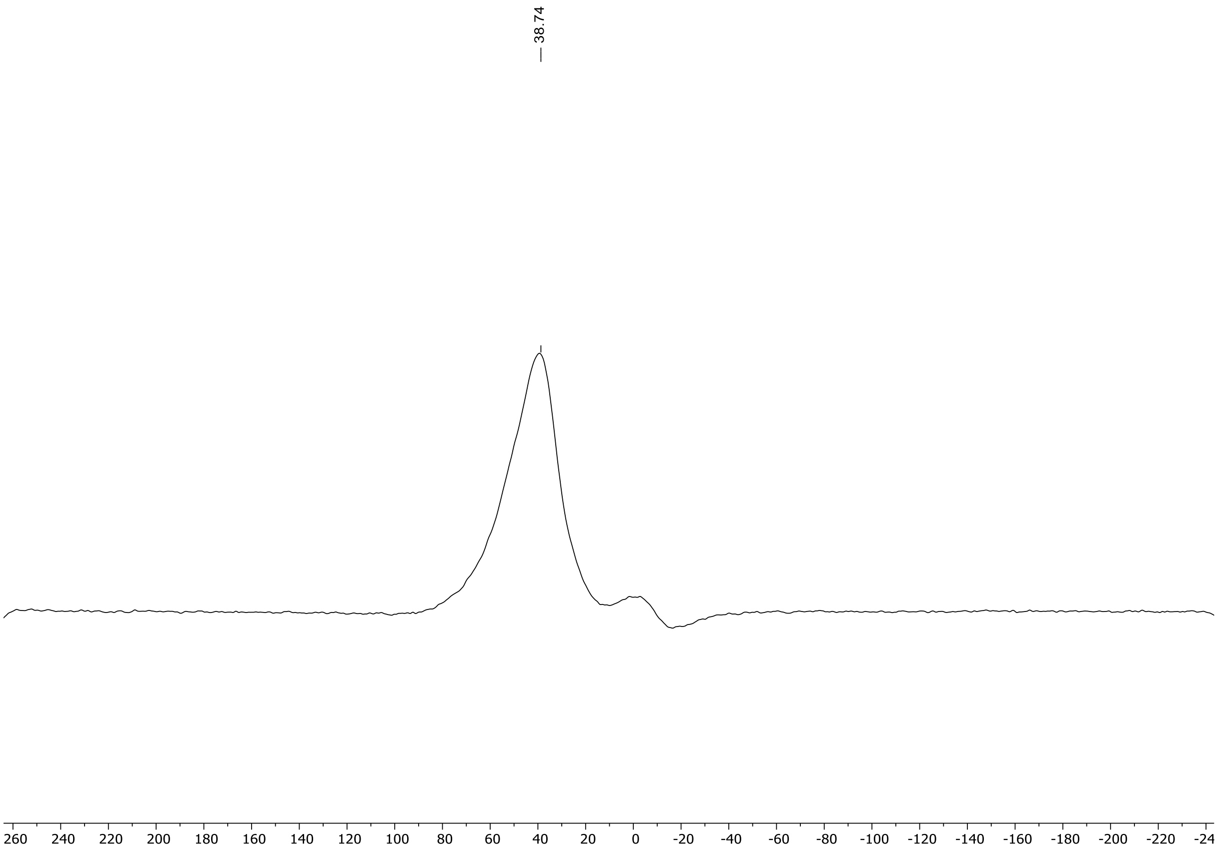

Figure S36. $^{11}B\{^{1}H\}$ NMR spectrum of $[^{Mes}Ter_2BO][K(18c6)]$ (**5a**) (96 MHz, C_6D_6, 298 K); .

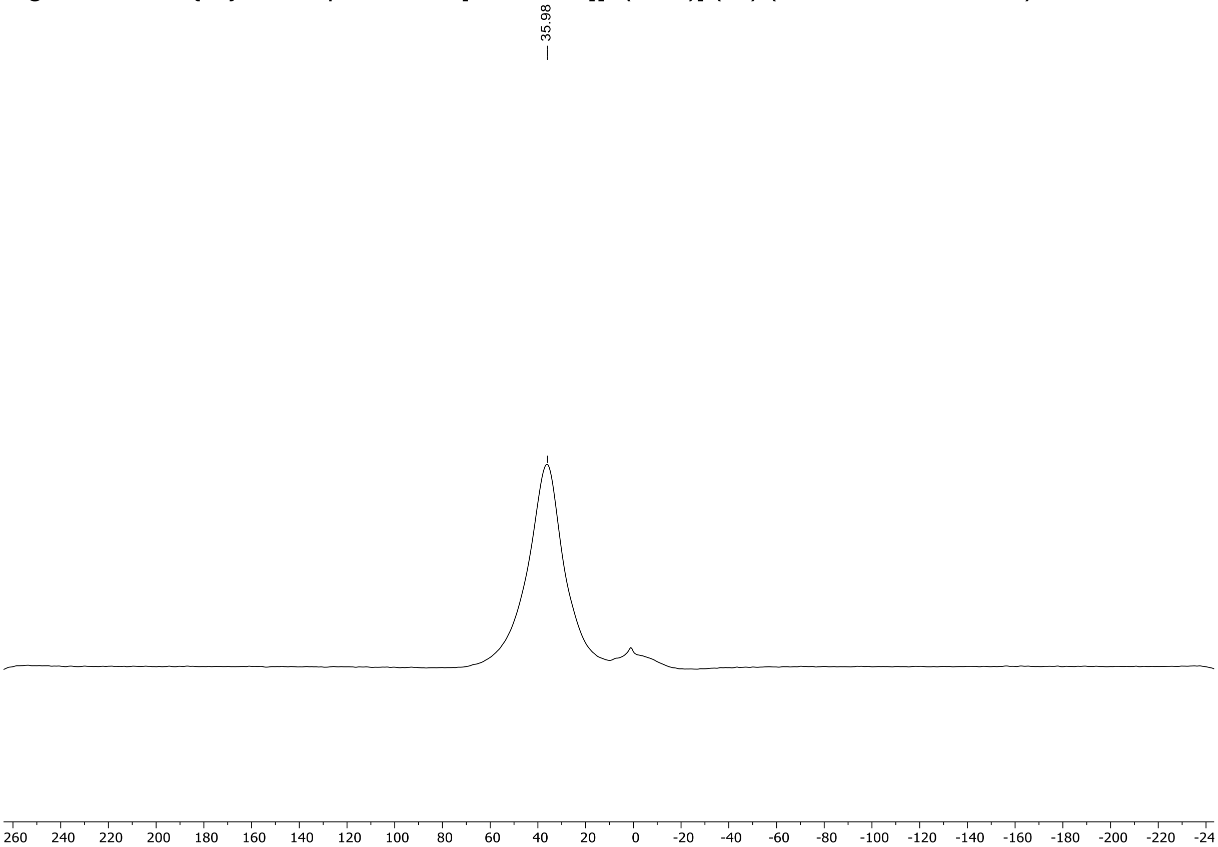

Figure S37. $^{11}B\{^{1}H\}$ NMR spectrum of $[^{Mes}Ter_2BO][K(18c6)]$ (**5a**) (96 MHz, THF-d_8, 298 K).

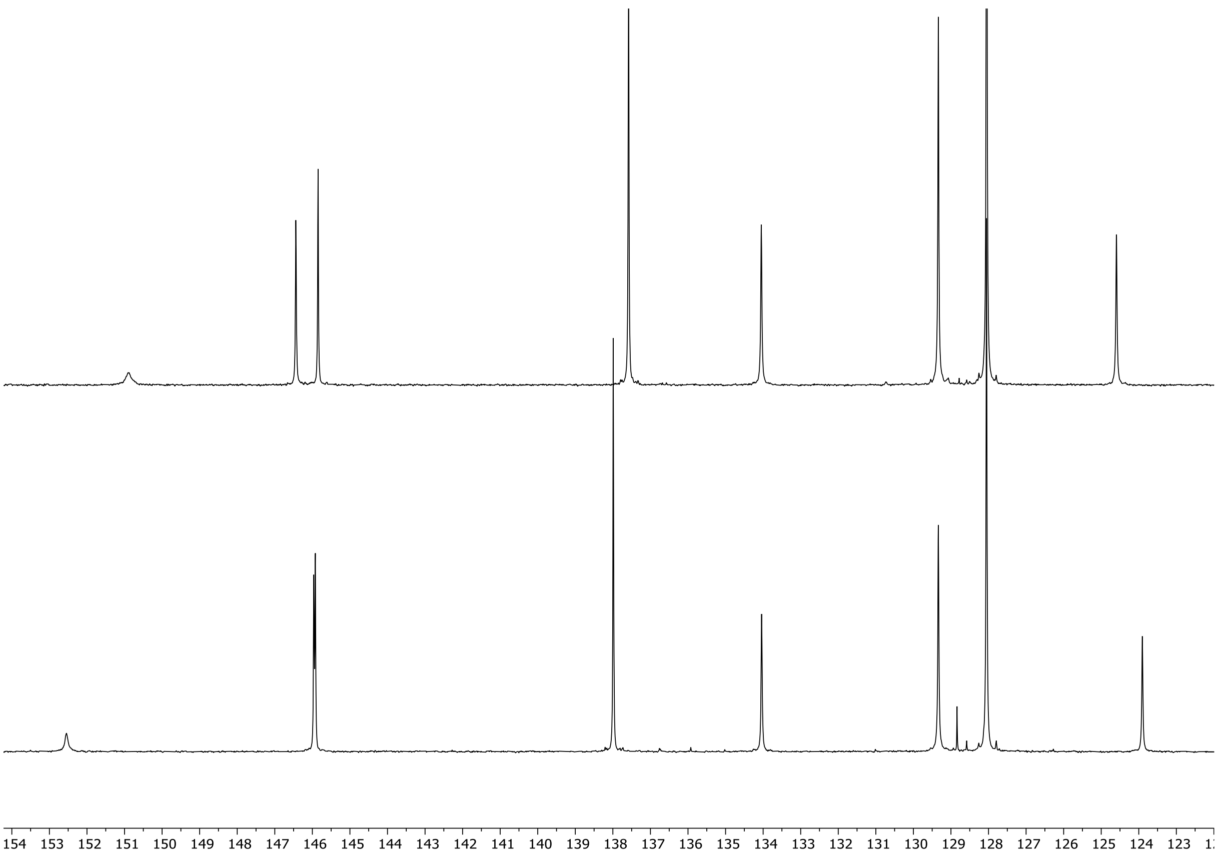

Figure S38. Excerpt of the $^{13}C\{^{1}H\}$ NMR spectra of [$^{Mes}Ter_2BO$][K(18c6)] (**5a**) (bottom) and [$^{Mes}Ter_2BOK$]$_2$ (**4**) (top) (126 MHz, THF-d_8, 298 K).

Synthesis of [^{Mes}Ter$_2$BO][K(2.2.2-crypt)] (5b)

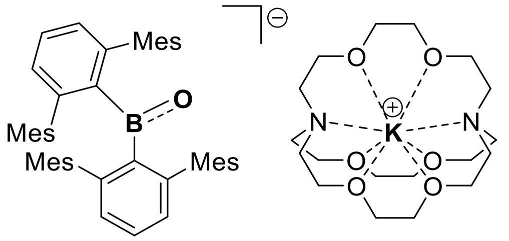

A) ^{Mes}Ter$_2$BOH (**2**) (0.030 g, 0.046 mmol) was dissolved in 0.5 mL of benzene, followed by the addition of K{N(SiMe$_3$)$_2$} (0.009 g, 0.046 mmol) in 0.5 mL of benzene. The reaction mixture was stirred for 15 minutes at room temperature to facilitate the *in situ* generation of [^{Mes}Ter$_2$BOK]$_2$ (**4**). All volatile components were then removed under vacuum. The resulting solid was dissolved in 0.5 mL of THF-d_8, transferred to a J-Young NMR tube and 2.2.2-crypt (0.017 g, 0.046 mmol) was added. Multinuclear NMR spectroscopy revealed the clean formation of [^{Mes}Ter$_2$BO][K(2.2.2-crypt)] (**5b**). NMR data was collected at that point. All volatile components were removed under vacuum to give **5b** as a colourless solid.
B) [(^{Mes}Ter)$_2$BOK]$_2$ (0.030 g, 0.22 mmol) and 2.2.2-crypt (0.016 g, 0.44 mmol) were dissolved in 0.5 mL of C_6D_6, resulting in the clean formation of [^{Mes}Ter$_2$BO][K(2.2.2-crypt)] (**5b**), as confirmed by ^{1}H NMR spectroscopy. All volatile components were then removed under vacuum to give **5b** as a colourless solid.
Despite multiple attempts, crystals of **5b** suitable for single crystal X-ray diffraction could not be obtained. The attempts included slow evaporation of saturated and unsaturated solutions, vapor diffusion, slow cooling of saturated solutions, and the use of the following solvents and their combinations: benzene, toluene, mesitylene, THF, n-hexane, n-pentane, dichloromethane, and 1,2-difluorobenzene.

Yield: 0.044 g (0.041 mmol; 93% (**B**)).
^{1}H NMR (400 MHz, THF-d_8, 298 K): δ = 1.73 (s, 24H, *o*-CH_3-C$_6$H$_3$), 2.25 (s, 12H, *p*-CH_3-C$_6$H$_3$), 2.49-2.51 (m, 12H, CH$_2$), 3.48-3.50 (m, 12H, CH$_2$), 3.53 (s, 12H, CH$_2$), 6.18 (d, $^3J_{H,H}$ = 7.5 Hz, 4H, *m*-CH$_{Aryl}$-B), 6.50 (s, 8H, *m*-CH_{Aryl}-C$_6$H$_3$), 6.63 (t, $^3J_{H,H}$ = 7.5 Hz, *p*-CH$_{Aryl}$-B) ppm.
^{13}C{^{1}H} NMR (126 MHz, THF-d_8, 298 K): δ = 21.4 (*p*-$\underline{C}$H$_3$-C$_6$H$_3$), 22.9 (*o*-$\underline{C}$H$_3$-C$_6$H$_3$), 54.6 (CH$_2$), 68.2 (CH$_2$), 71.2 (CH$_2$), 123.0 (*p*-CH$_{Aryl}$-B), 127.7 (*m*-CH_{Aryl}-C$_6$H$_3$), 128.7 (*m*-CH$_{Aryl}$-B), 132.9 (C$_{q,Aryl}$), 138.2 (C$_{q,Aryl}$), 145.8 (C$_{q,Aryl}$), 146.4 (C$_{q,Aryl}$), 153.6 (br, C$_{q,Aryl}$B) ppm.
^{11}B/^{11}B{^{1}H} NMR (96 MHz, THF-d_8, 298 K): δ = 34.9 ppm.
HR/MS: m/z calcd.: 653.3968 [$C_{48}H_{50}BO$]$^-$ measured (ESI, negative): m/z 653.3963.
HR/MS: m/z calcd.: 415.2205 [$C_{18}H_{36}N_2O_6K$]$^+$ measured (ESI, positive): m/z 415.2197.
EA: Anal. calcd. for $C_{66}H_{86}BKN_2O_7$: C, 74.13; H, 8.11; N, 2.62; Found: C, 73.40; H, 8.05; N, 2.70.

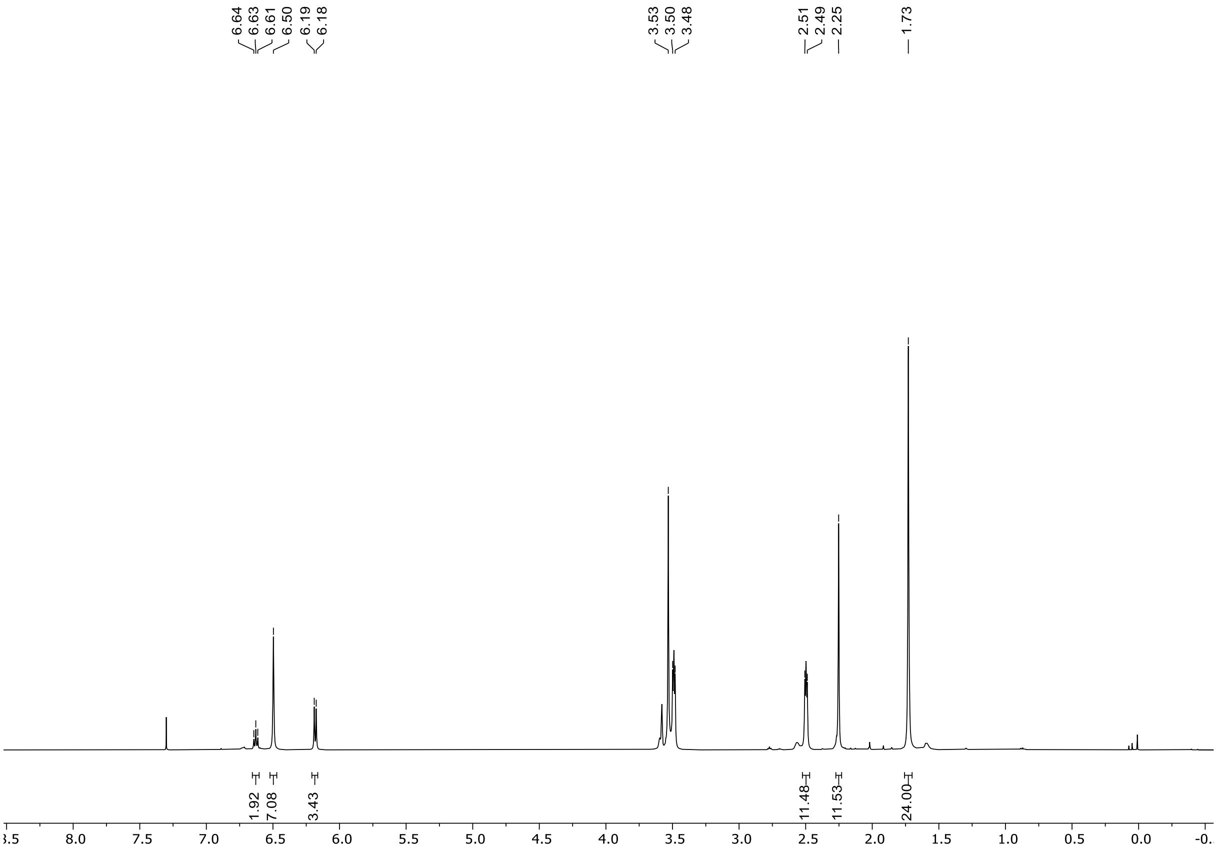

Figure S39. ^{1}H NMR spectrum of [^{Mes}Ter$_2$BO][K(2.2.2-crypt)] (**5b**) (400 MHz, THF-d_8, 298 K); 7.31 ppm: benzene.

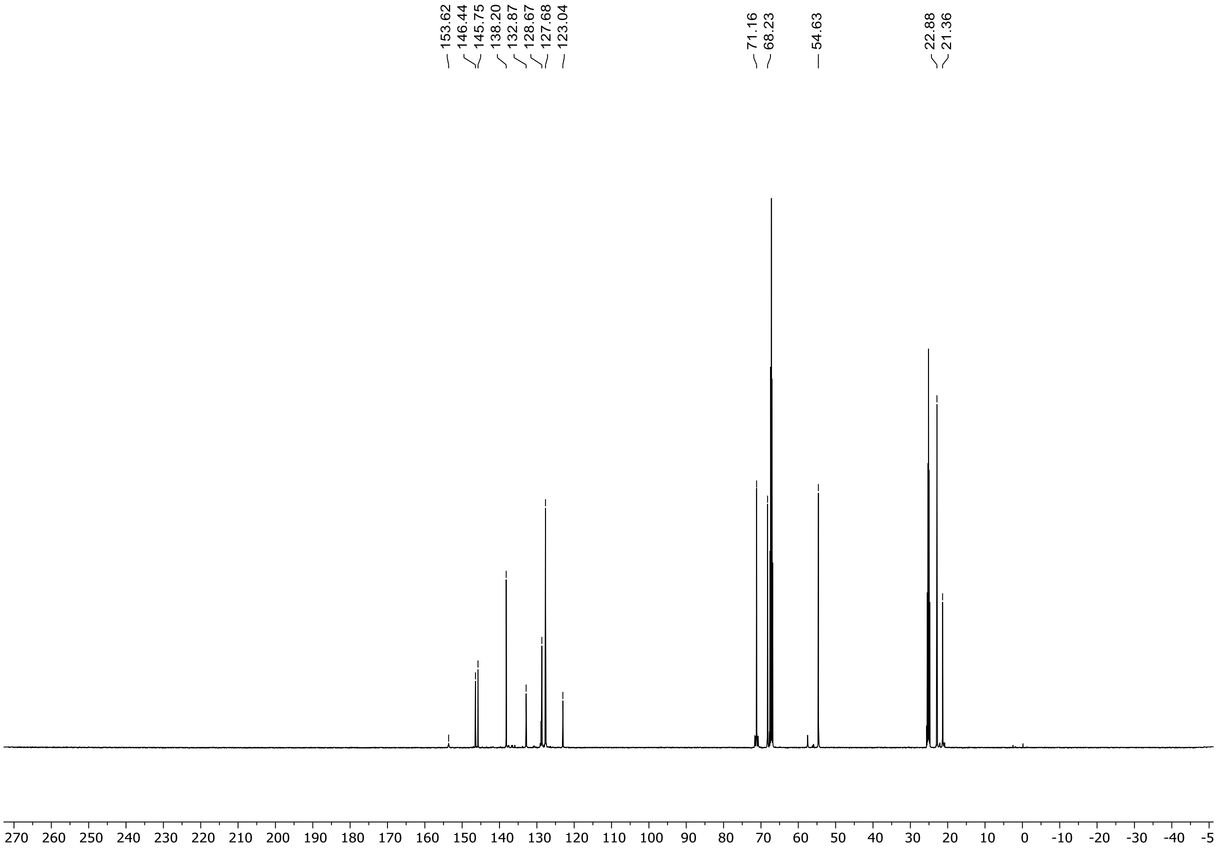

Figure S40. ^{13}C{^{1}H} NMR spectrum of [^{Mes}Ter$_2$BO][K(2.2.2-crypt)] (**5b**) (126 MHz, THF-d_8, 298 K).

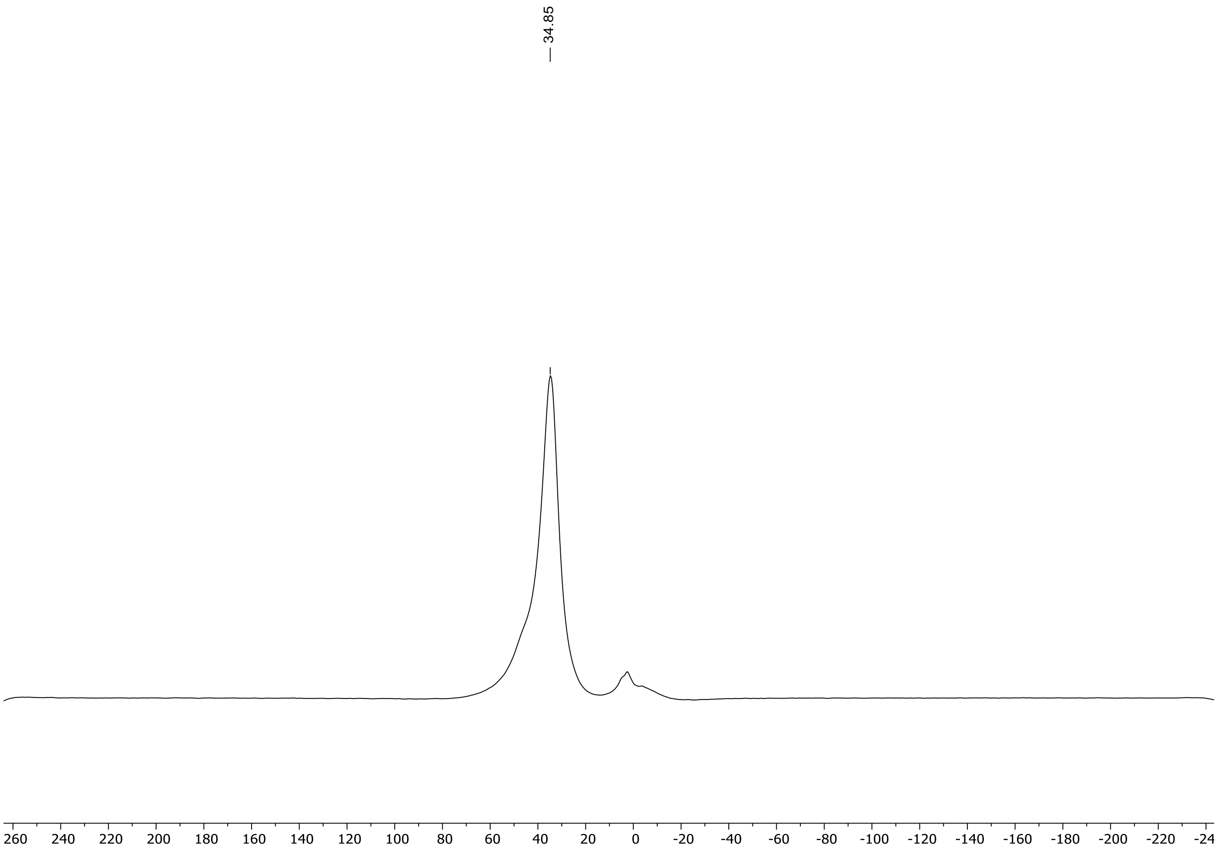

Figure S41. $^{11}B\{^{1}H\}$ NMR spectrum of [$^{Mes}Ter_2BO$][K(2.2.2-crypt)] (**5b**) (96 MHz, THF-d_8, 298 K).

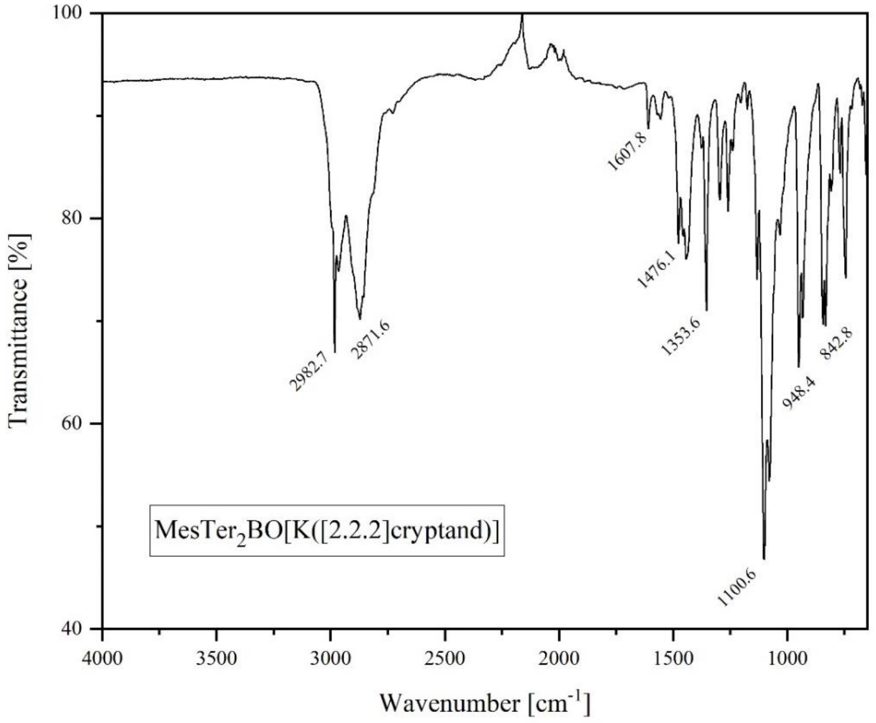

Figure S42. IR spectrum of [$^{Mes}Ter_2BO$][K(2.2.2-crypt)] (**5b**).

IR (ATR): $\tilde{\nu}$ [cm^{-1}] = 2983 (s), 2965 (m), 2872 (s), 2729 (w), 1608 (w), 1555 (w), 1476 (m), 1456 (m), 1442 (m), 1375 (w), 1354 (s), 1295 (m), 1259 (m), 1239 (w), 1204 (w), 1174 (w), 1131 (m), 1101 (s), 1077 (s), 1031 (w), 948 (s), 931 (m), 843 (m), 831 (m), 806 (w), 768 (w), 744 (m), 654 (w).

Synthesis of [^{Mes}Ter$_2$B-μ-S$_2$C=S][K(18c6)] (6a)

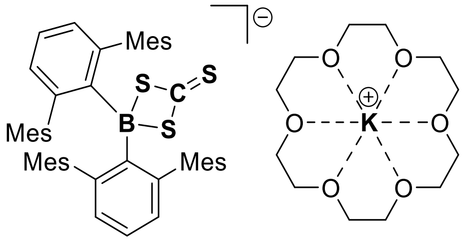

[^{Mes}Ter$_2$BOK]$_2$ (**4**) (0.015 g, 0.011 mmol) was dissolved in 0.5 mL of THF-d_8, followed by the addition of 18c6 (0.006 g, 0.022 mmol). The clean formation of [^{Mes}Ter$_2$BO][K(18c6)] (**5a**) was confirmed by ^{1}H NMR spectroscopy (Figure S43, bottom). An excess of CS_2 (2 drops with a 1 mL syringe) was then added, and the reaction progress was monitored by ^{1}H NMR spectroscopy (Figure S43). It is worth noting, that despite using freshly distilled CS_2 which was stored over molecular sieves and freeze-pump-thaw degassed three times prior to use, the formation of the borinic acid ^{Mes}Ter$_2$BOH (**2**) as a by-product is observed, likely due to hydrolysis. After the starting material **5a** has been consumed completely, the formation of [^{Mes}Ter$_2$B-μ-S$_2$C=S][K(18c6)] (**6a**) was verified by multinuclear NMR spectroscopy at this point. Upon leaving the reaction mixture undisturbed over the course of two days, colourless crystals of **6a** suitable for single crystal X-ray diffraction were obtained (Figure S47).
By reacting **5a** and CS_2 in a more concentrated C_6D_6 solution, the formation of colourless crystals suitable for single crystal X-ray diffraction was observed after a few minutes at room temperature and the reaction mixture was left undisturbed for 16 hours. The colourless crystalline material obtained was suitable for single crystal X-ray diffraction and was confirmed to be [(^{Mes}Ter)$_2$B(-μ-S$_{2-x}$O$_x$)C=(S$_{1-x}$O$_x$)] due to oxygen / sulfur scrambling across all three positions (Figure S48).

^{1}H NMR (400 MHz, THF-d_8, 298 K): δ = 1.03 (s, 6H, CH$_3$), 1.66 (s, 6H, CH$_3$), 1.67 (s, 6H, CH$_3$), 1.97 (s, 6H, CH$_3$), 2.09 (s, 6H, CH$_3$), 2.17 (s, 6H, CH$_3$), 3.60 (s, 24H, CH$_2$), 6.25 (dd, J = 7.4 Hz, J = 1.6 Hz, 2H, CH$_{Aryl}$), 6.37 (s, 2H, CH$_{Aryl}$), 6.46 (s, 2H, CH$_{Aryl}$), 6.50-6.52 (m, 6H, CH$_{Aryl}$), 6.85 (t, $^3J_{H,H}$ = 7.4 Hz, 2H, CH$_{Aryl}$) ppm.
^{13}C{^{1}H} NMR (126 MHz, THF-d_8, 298 K): δ = 21.0 (CH$_3$), 21.2 (CH$_3$), 21.4 (CH$_3$), 23.3 (CH$_3$), 23.4 (CH$_3$), 23.7 (CH$_3$), 71.2 (CH$_2$), 124.7 (CH$_{Aryl}$), 127.3 (CH$_{Aryl}$), 128.7 (CH$_{Aryl}$), 128.9 (CH$_{Aryl}$), 130.0 (CH$_{Aryl}$), 130.9 (CH$_{Aryl}$), 131.3 (CH$_{Aryl}$), 132.8 (C$_{q,Aryl}$), 138.4 (C$_{q,Aryl}$), 139.8 (C$_{q,Aryl}$), 140.4 (C$_{q,Aryl}$), 146.3 (C$_{q,Aryl}$), 146.8 (C$_{q,Aryl}$), 148.6 (C$_{q,Aryl}$), 150.4 (C$_{q,Aryl}$), 152.1 (br, C$_{q,Aryl}$B), 236.2 (S$_2$C=S) ppm.
^{11}B/^{11}B{^{1}H} NMR (96 MHz, THF-d_8, 298 K): δ = -8.6 ppm.

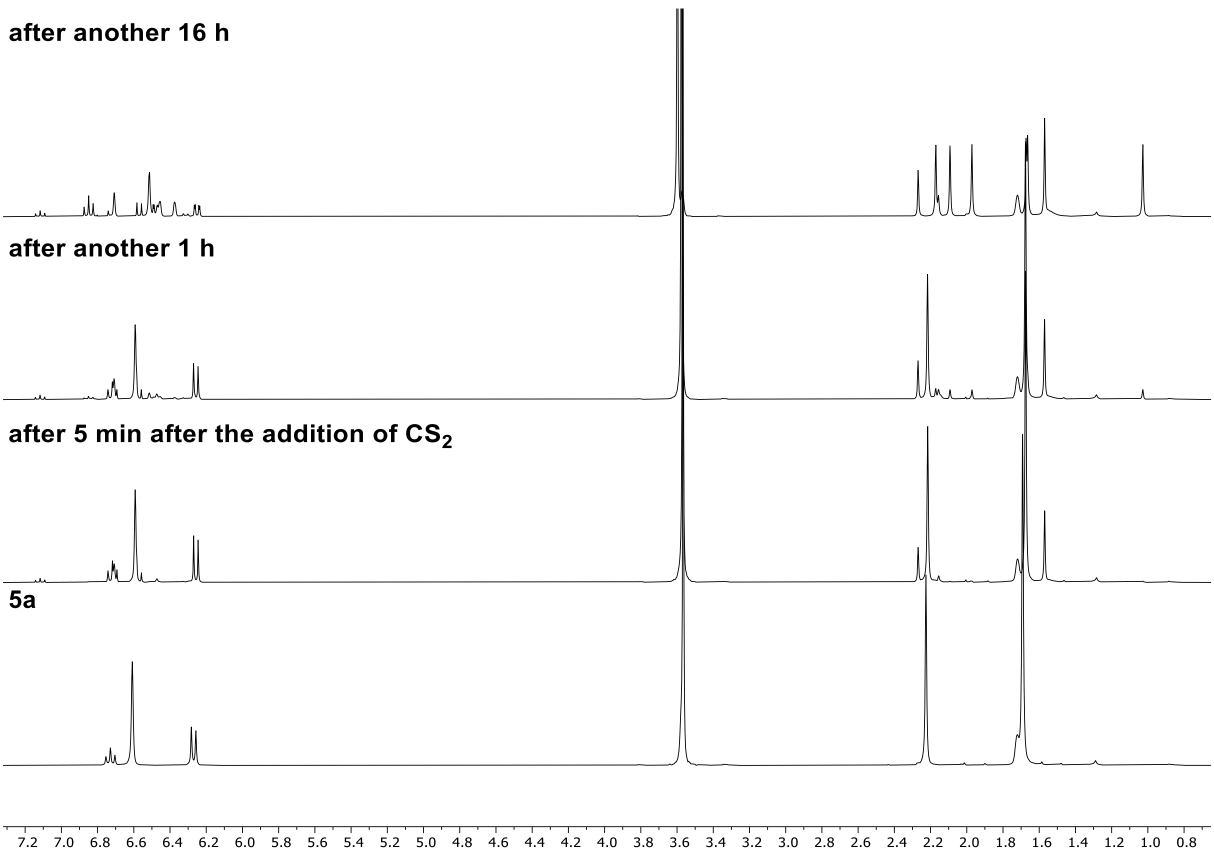

Figure S43. Monitoring of the reaction of [$^{Mes}Ter_2BO$][K(18c6)] (**5a**) with CS_2 by ^1H NMR spectroscopy (300 MHz, C_6D_6, 298 K).

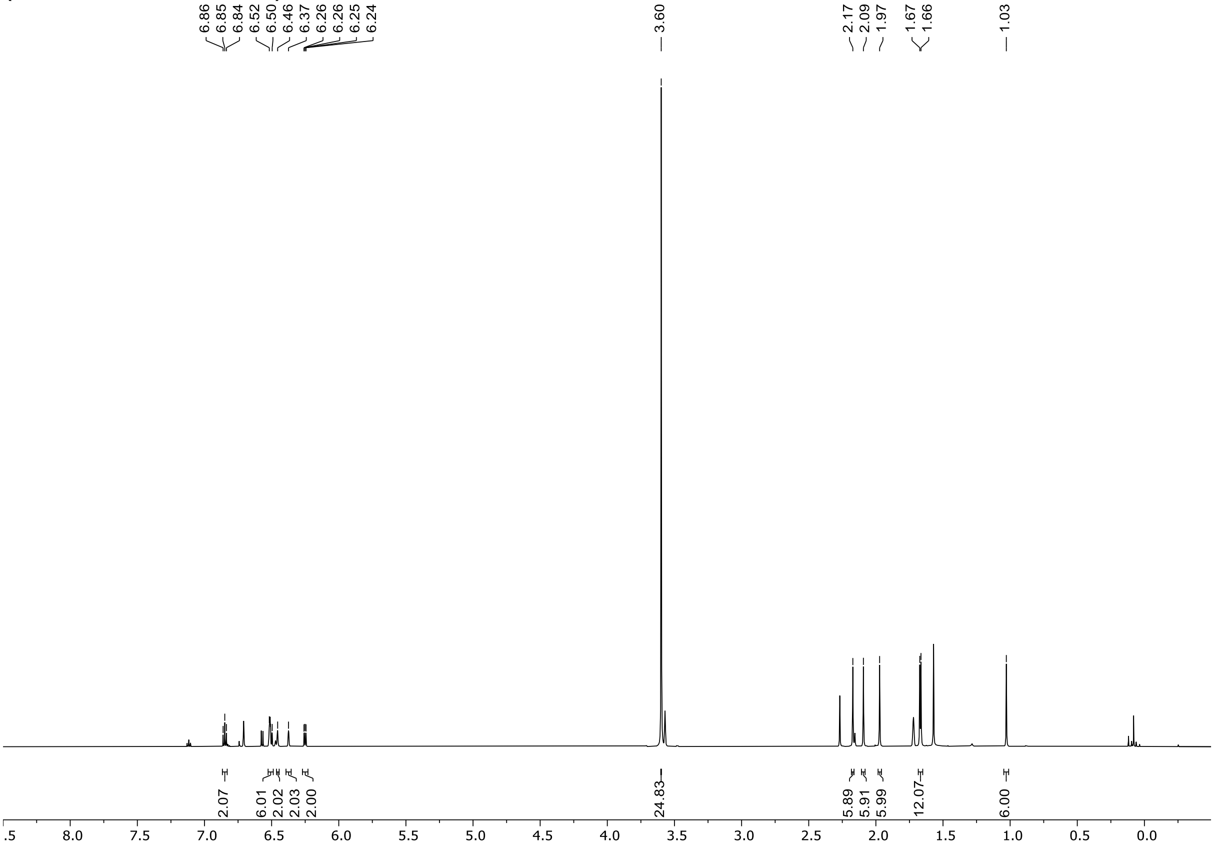

Figure S44. ^1H NMR spectrum after the reaction of [$^{Mes}Ter_2BO$][K(18c6)] (**5a**) with CS_2 (400 MHz, THF-d_8, 298 K); selected signals correspond to [$^{Mes}Ter_2B$-μ-S_2C=S][K(18c6)] (**6a**); the unselected signals are attributed to $^{Mes}Ter_2BOH$ (**2**).

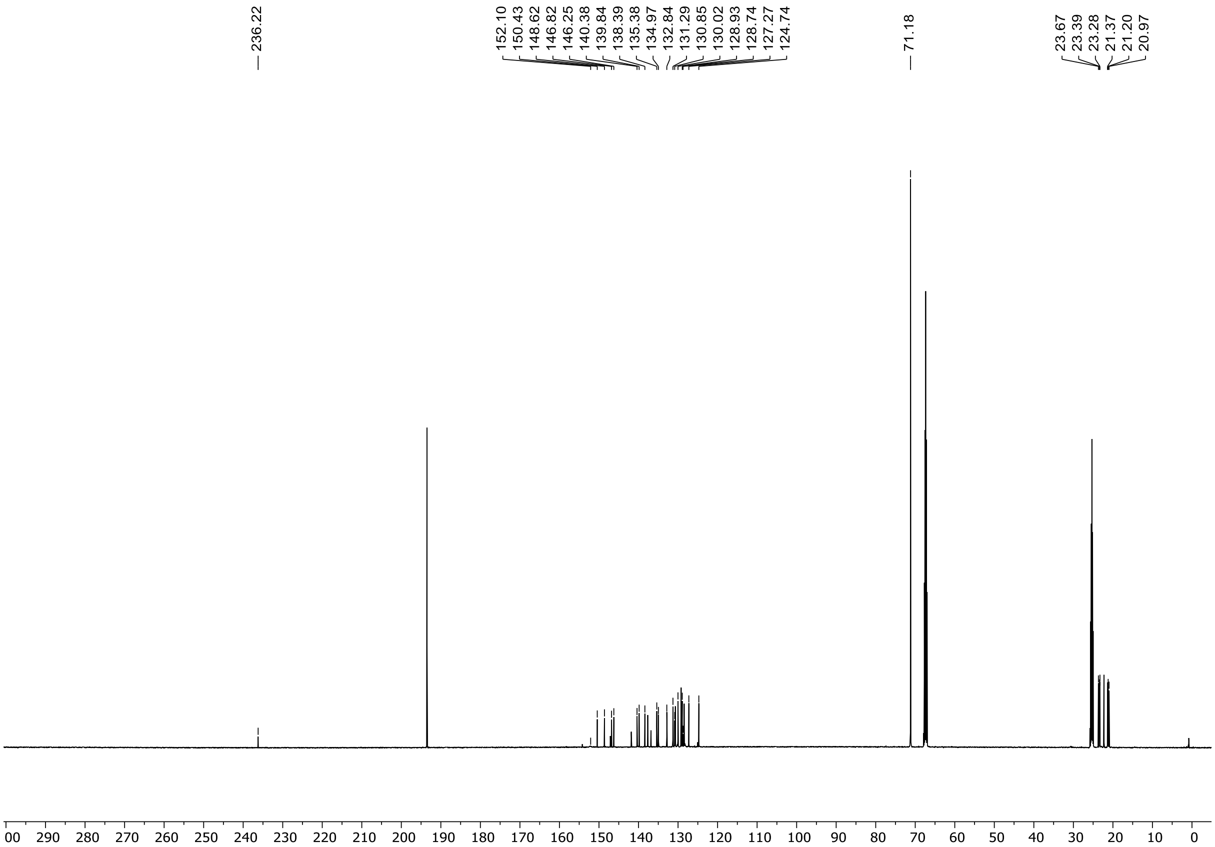

Figure S45. $^{13}C\{^1H\}$ NMR spectrum after the reaction of [$^{Mes}Ter_2BO$][K(18c6)] (**5a**) with CS_2 (126 MHz, THF-d_8, 298 K); selected signals correspond to [$^{Mes}Ter_2B$-μ-S_2C=S][K(18c6)] (**6a**); the unselected signals are attributed to $^{Mes}Ter_2BOH$ (**2**) and CS_2 (193.5 ppm).

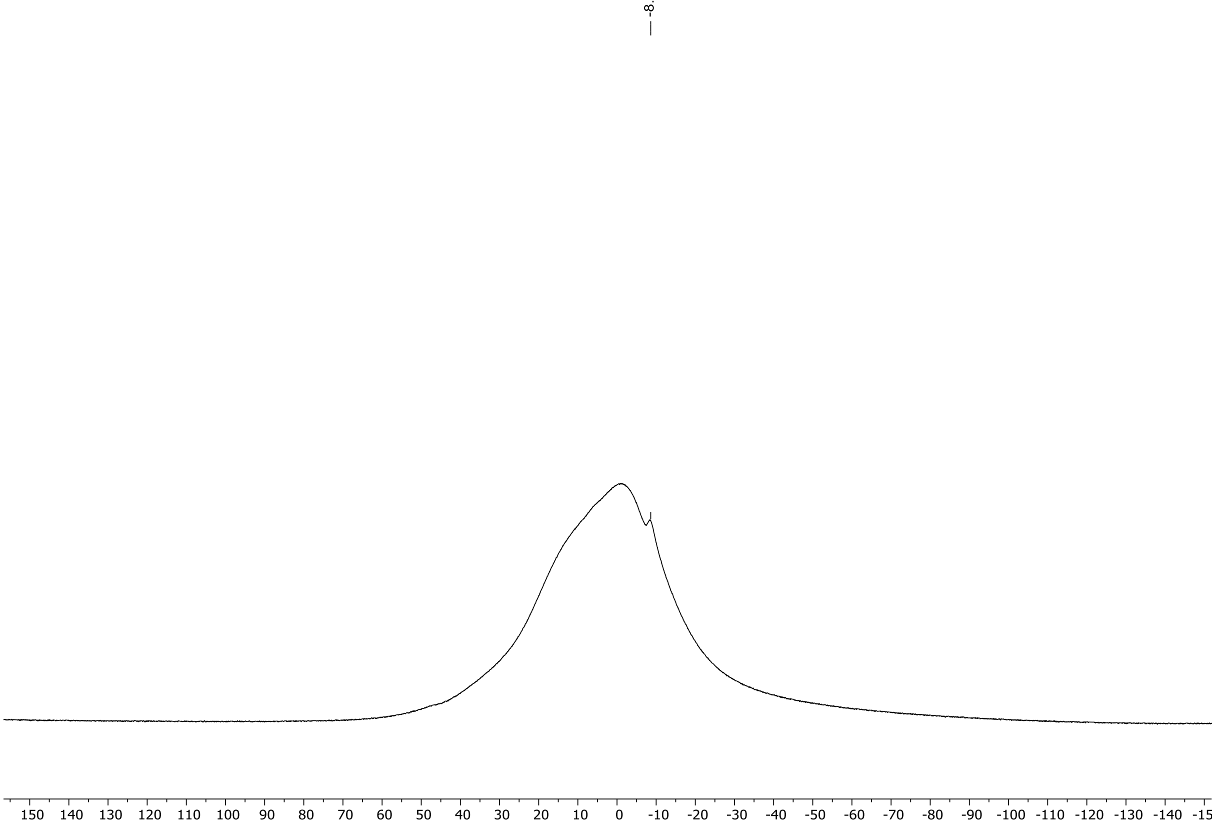

Figure S46. $^{11}B\{^1H\}$ NMR spectrum of [$^{Mes}Ter_2B$-μ-S_2C=S][K(18c6)] (**6a**) (96 MHz, THF-d_8, 298 K).

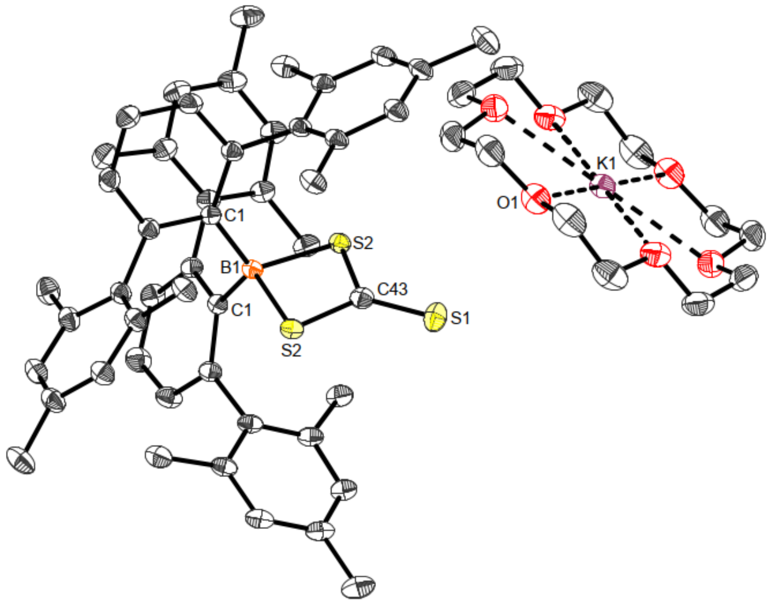

Figure S47. Molecular structure of [$^{Mes}Ter_2B$-μ-S_2C=S][K(18c6)] (**6a**) in the crystal. Anisotropic displacement parameters are drawn at the 50% probability level (hydrogen atoms and lattice solvent have been omitted for clarity). Selected bond lengths (Å) and angles (deg): B1–S2 1.9824(16), B1–C1 1.6517(17), S1–C43 1.662(2), S2–C43 1.7222(13), S2–B1–S2' 87.16(9), C1–B1–C1' 117.09(15).

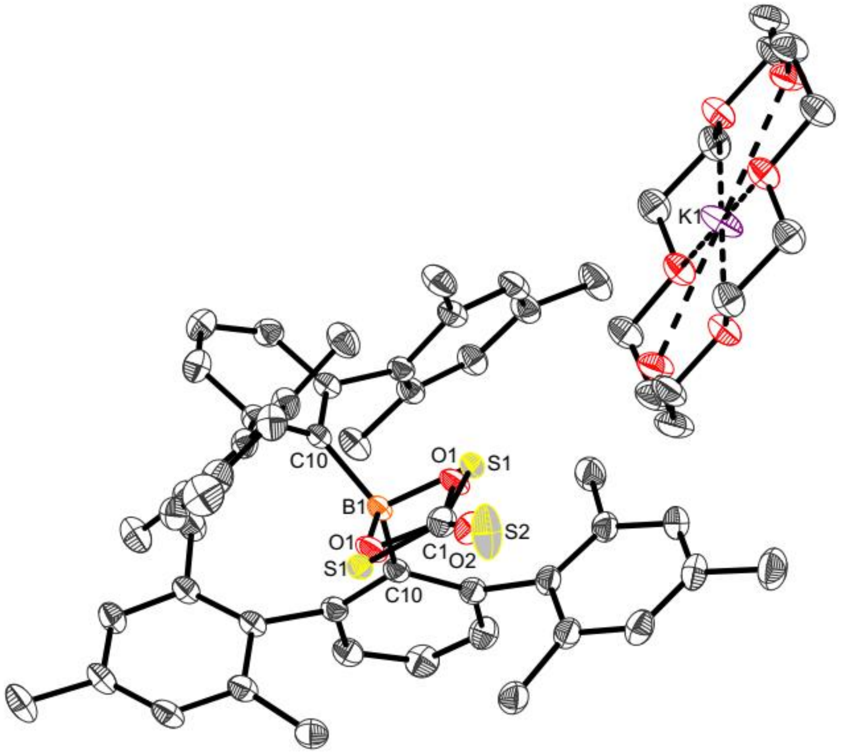

Figure S48. Molecular structure of [($^{Mes}Ter)_2B(-\mu$-$S_{2\text{-}x}O_x)C=(S_{1\text{-}x}O_x)$] in the crystal. Anisotropic displacement parameters are drawn at the 50% probability level (hydrogen atoms and lattice solvent have been omitted for clarity). Selected bond lengths (Å) and angles (deg): B1–S1 1.939(2), B1–O1 1.59(2), B1–C10 1.6431(14), C1–S1 1.676(2), C1–S2 1.708(3), C1–O1 1.462(11), C1–O2 1.058(13), S1–B1–S1 88.99(12), O1–B1–O1' 74.7(10), C10–B1–C10' 118.48(13).

Synthesis of [^{Mes}Ter$_2$B-μ-S$_2$C=S][K(2.2.2-crypt)] (6b)

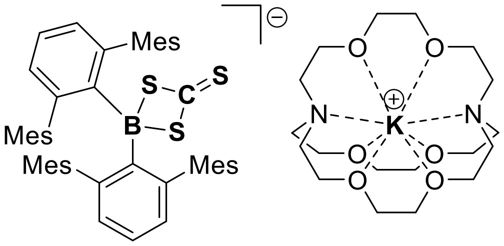

[^{Mes}Ter$_2$BOK]$_2$ (**4**) (0.030 g, 0.022 mmol) was dissolved in 0.5 mL of THF-d_8, followed by the addition of [2.2.2-crypt] (0.016 g, 0.043 mmol). The clean formation of [^{Mes}Ter$_2$BO][K(2.2.2-crypt)] (**5b**) was confirmed by ^{1}H NMR spectroscopy. An excess of CS$_2$ (2 drops with a 1 mL syringe) was then added, and subsequent analysis by multinuclear NMR spectroscopy revealed the formation of [^{Mes}Ter$_2$B-μ-S$_2$C=S][K(2.2.2-crypt)] (**6b**). All volatile components were then removed under vacuum, yielding **6b** as a slightly yellow solid. Crystals of **6b** suitable for single crystal X-ray diffraction were obtained by slow diffusion of *n*-hexane into a solution of **6b** in THF at -30 °C.

Yield: 0.041 g (0.035 mmol; 81%).
^{1}H NMR (400 MHz, THF-d_8, 298 K): δ = 1.05 (s, 6H, CH$_3$), 1.69 (s, 12H, CH$_3$), 2.00 (s, 6H, CH$_3$), 2.11 (s, 6H, CH$_3$), 2.18 (s, 6H, CH$_3$), 2.56-2.58 (m, 12H, CH$_2$), 3.54-3.56 (m, 12H, CH$_2$), 3.59 (s, 12H, CH$_2$), 6.27 (dd, J = 7.4 Hz, J = 1.6 Hz, 2H, CH$_{Aryl}$), 6.39 (s, 2H, CH$_{Aryl}$), 6.48 (m, 2H, CH$_{Aryl}$), 6.51-6.54 (m, 6H, CH$_{Aryl}$), 6.87 (t, $^3J_{H,H}$ = 7.4 Hz, 2H, CH$_{Aryl}$) ppm.
^{13}C{^{1}H} NMR (126 MHz, THF-d_8, 298 K): δ = 20.8 (CH$_3$), 21.1 (CH$_3$), 21.3 (CH$_3$), 23.1 (CH$_3$), 23.3 (CH$_3$), 23.6 (CH$_3$), 54.7 (CH$_2$), 68.4 (CH$_2$), 71.2 (CH$_2$), 124.6 (CH$_{Aryl}$), 127.1 (CH$_{Aryl}$), 128.4 (CH$_{Aryl}$), 128.8 (CH$_{Aryl}$), 129.9 (CH$_{Aryl}$), 130.6 (CH$_{Aryl}$), 131.2 (CH$_{Aryl}$), 132.7 (C$_{q,Aryl}$), 134.8 (C$_{q,Aryl}$), 135.3 (C$_{q,Aryl}$), 138.3 (C$_{q,Aryl}$), 139.7 (C$_{q,Aryl}$), 140.3 (C$_{q,Aryl}$), 146.2 (C$_{q,Aryl}$), 146.7 (C$_{q,Aryl}$), 148.6 (C$_{q,Aryl}$), 150.4 (C$_{q,Aryl}$), 152.2 (br, C$_{q,Aryl}$B), 236.0 (S$_2$C=S) ppm.
^{11}B/^{11}B{^{1}H} NMR (96 MHz, THF-d_8, 298 K): δ = -8.5 ppm.
HR/MS: m/z calcd.: 745.3182 [C$_{49}$H$_{50}$BS$_3$]$^-$ measured (ESI, negative): m/z 745.3160.
HR/MS: m/z calcd.: 415.2205 [C$_{18}$H$_{36}$N$_2$O$_6$K]$^+$ measured (ESI, positive): m/z 415.2211.
EA: Anal. calcd. for C$_{67}$H$_{86}$BKN$_2$O$_6$S$_3$: C, 69.28; H, 7.46; N, 2.41; S, 8.28; Found: C, 68.05; H, 7.64; N 2.27; S 7.81.

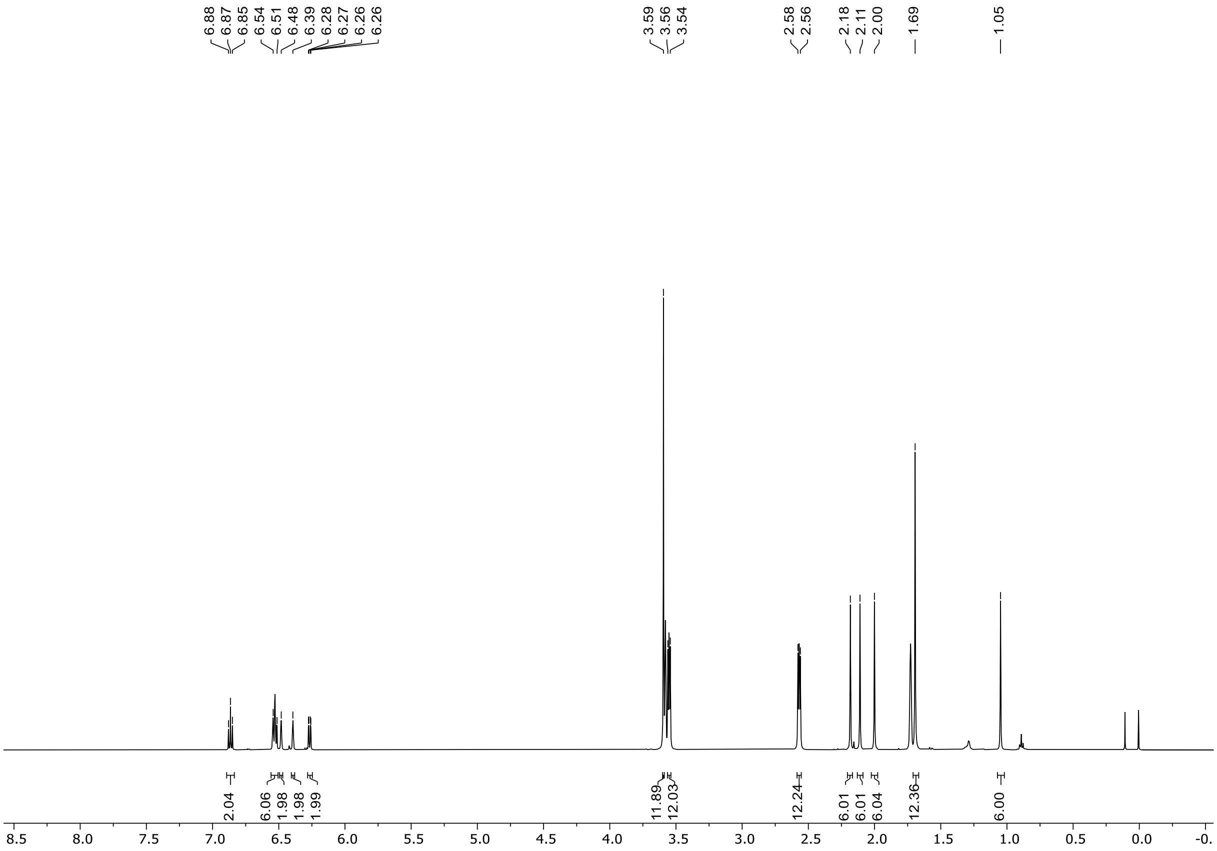

Figure S49. ^{1}H NMR spectrum of $[^{Mes}Ter_2B\text{-}\mu\text{-}S_2C{=}S][K(2.2.2\text{-crypt})]$ (**6b**) (400 MHz, THF-d_8, 298 K).

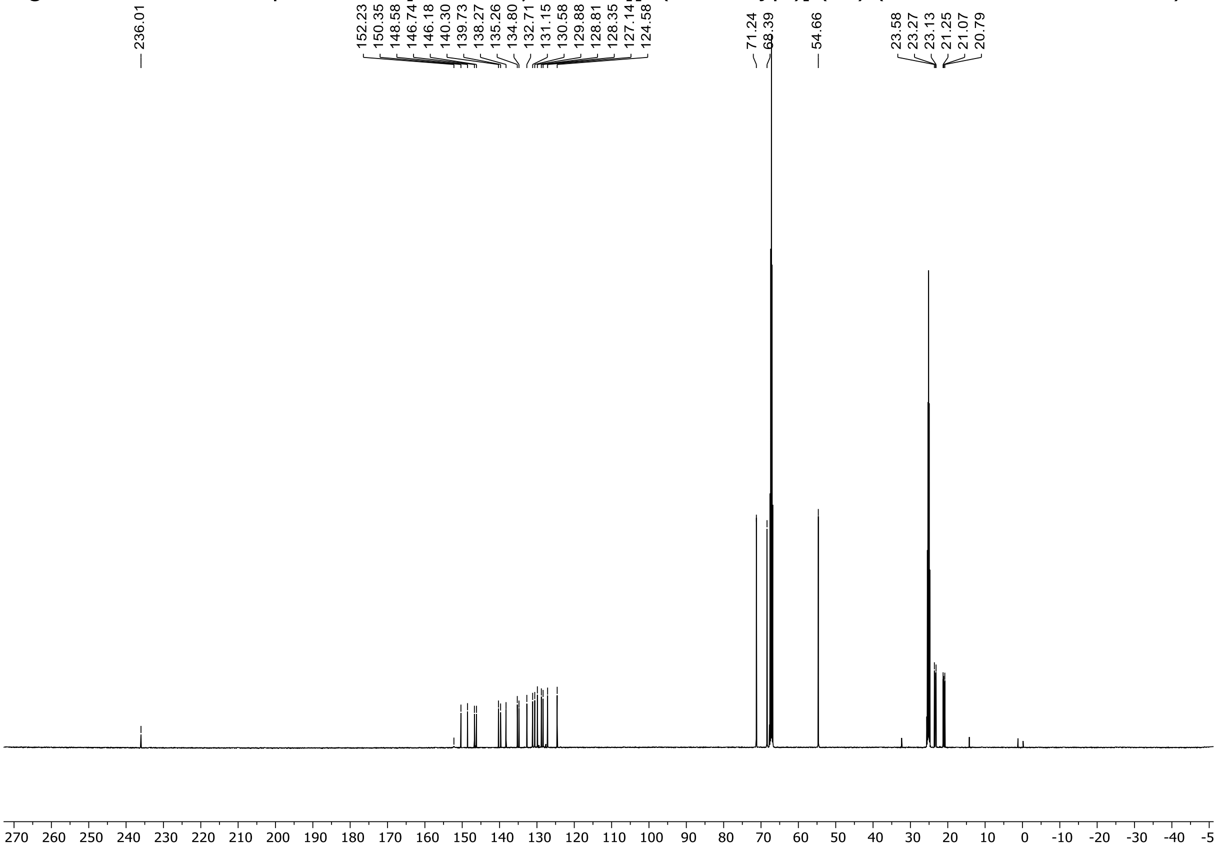

Figure S50. $^{13}C\{^{1}H\}$ NMR spectrum of $[^{Mes}Ter_2B\text{-}\mu\text{-}S_2C{=}S][K(2.2.2\text{-crypt})]$ (**6b**) (126 MHz, THF-d_8, 298 K).

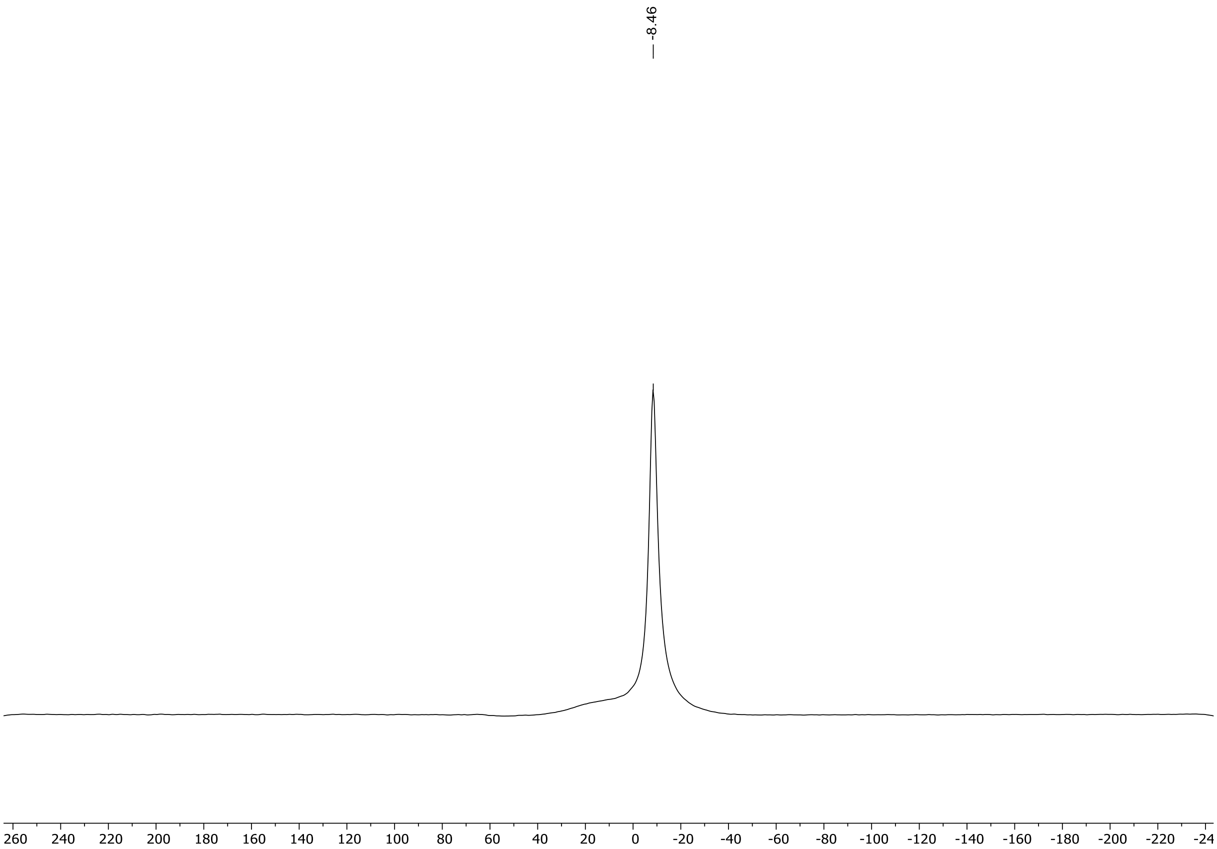

Figure S51. $^{11}B\{^1H\}$ NMR spectrum of [$^{Mes}Ter_2B$-μ-S_2C=S][K(2.2.2-crypt)] (**6b**) (96 MHz, THF-d_8, 298 K).

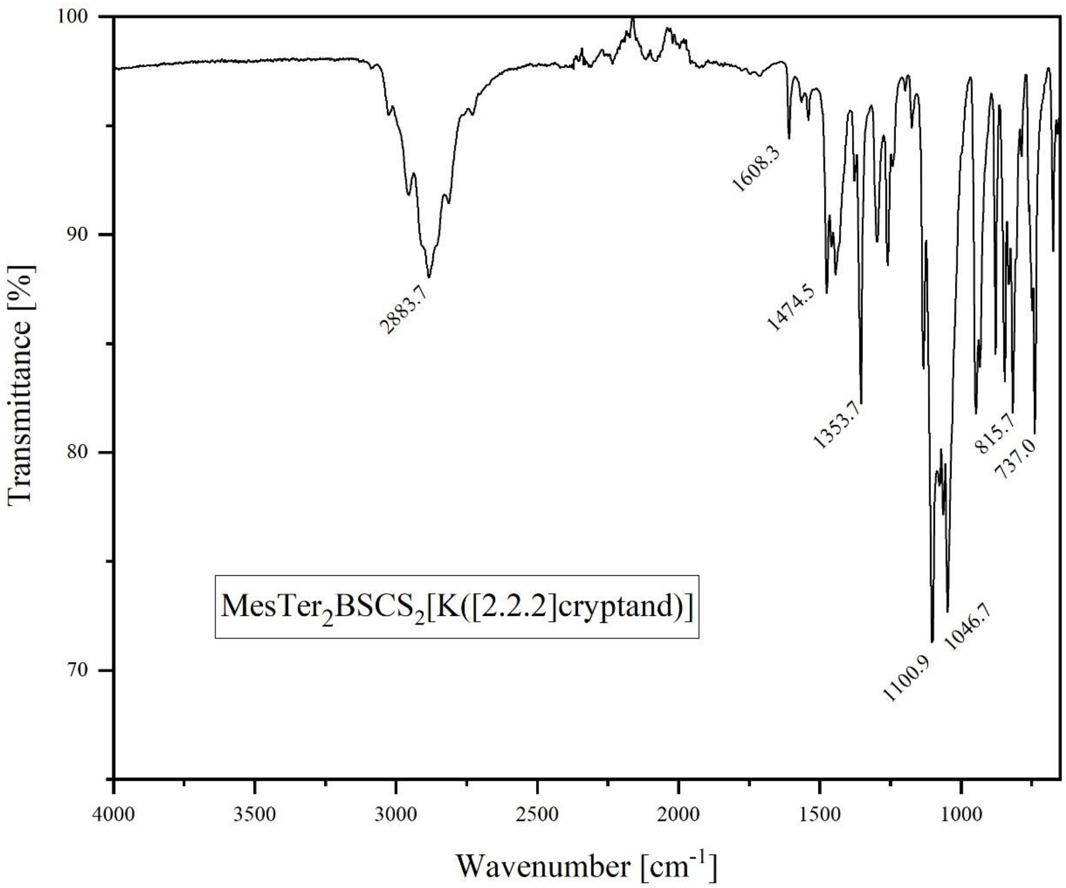

Figure S52. IR spectrum of [^{Mes}Ter$_2$B-μ-S$_2$C=S][K(2.2.2-crypt)] (**6b**).
IR (ATR): ṽ [cm^{-1}] = 3026 (w), 2956 (m), 2884 (m), 2814 (m), 2730 (w), 1608 (w), 1540 (w), 1475 (m), 1458 (m), 1443 (m), 1376 (w), 1354 (s), 1297 (m), 1259 (m), 1241 (w), 1173 (w), 1132 (m), 1101 (s), 1076 (s), 1062 (s), 1047 (s), 946 (s), 933 (m), 877 (m), 845 (m), 830 (m), 816 (s), 785 (w), 737 (s), 673 (m).

Synthesis of [^{Mes}Ter$_2$B-μ-O$_2$C=O][K(18c6)] (7a)

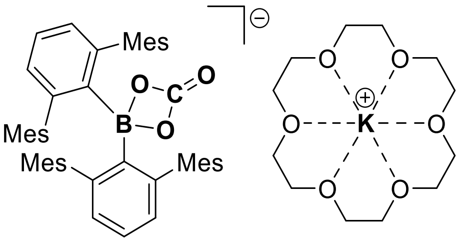

[^{Mes}Ter$_2$BOK]$_2$ (**4**) (0.022 g, 0.016 mmol) and 18c6 (0.009 g, 0.032 mmol) were dissolved in 0.5 mL of C_6D_6, and the resulting solution was transferred to a J. Young NMR tube. The solution underwent three cycles of freeze-pump-thaw degassing, after which 1 bar of CO_2 was introduced. Multinuclear NMR spectroscopy analysis of the reaction mixture revealed the predominant formation of [^{Mes}Ter$_2$B-μ-O$_2$C=O][K(18c6)] (**7a**), with minor amounts of MesTer$_2$BOH (**2**), likely due to slight hydrolysis. All volatile components were then removed under vacuum, yielding crude **7a** as a colourless solid.

Crude yield: 0.031 g (0.032 mmol; 99%).
^{1}H NMR (400 MHz, C_6D_6, 298 K): δ = 2.01 (s(br), 24H, *o*-C*H*$_3$-C$_6$H$_3$), 2.29 (s, 12H, *p*-C*H*$_3$-C$_6$H$_3$), 3.19 (s, 24H, CH$_2$), 6.82 (s(br), 8H, *m*-C*H*$_{Aryl}$-C$_6$H$_3$), 6.83 (d, $^3J_{H,H}$ = 7.5 Hz, 4H, *m*-CH$_{Aryl}$-B), 7.15 (t, $^3J_{H,H}$ = 7.5 Hz, 2H, *p*-CH$_{Aryl}$-B) ppm.
^{13}C{^{1}H} NMR (126 MHz, C_6D_6, 298 K): δ = 21.3 (*p*-<u>C</u>H$_3$-C$_6$H$_3$), 22.9 (*o*-<u>C</u>H$_3$-C$_6$H$_3$), 70.0 (CH$_2$), 125.8 (*p*-CH$_{Aryl}$B), 128.3 (*m*-<u>C</u>H$_{Aryl}$-C$_6$H$_3$)*, 130.5 (*m*-CH$_{Aryl}$B), 133.8 (C$_{q,Aryl}$), 138.0 (C$_{q,Aryl}$), 146.4 (C$_{q,Aryl}$), 148.2 (br, C$_{q,Aryl}$), 149.4 (br, C$_{q,Aryl}$B), 160.0 (O$_2$C=O) ppm.
* = overlap with C_6D_6 signal and assigned by ^{1}H/^{13}C-HMBC
^{11}B/^{11}B{^{1}H} NMR (96 MHz, C_6D_6, 298 K): δ = 7.5 ppm.
HR/MS: m/z calcd.: 698.3945 [$C_{49}H_{50}BO_3$+H]$^-$ measured (ESI, negative): m/z 698.33973.
HR/MS: m/z calcd.: 303.1204 [$C_{12}H_{24}O_6K]^+$ measured (ESI, positive): m/z 303.1195.

Note: The additional signals in the NMR spectra correspond to (^{Mes}Ter)$_2$BOH due to slight hydrolysis (approximately 10%) of the starting material after addition of CO_2.

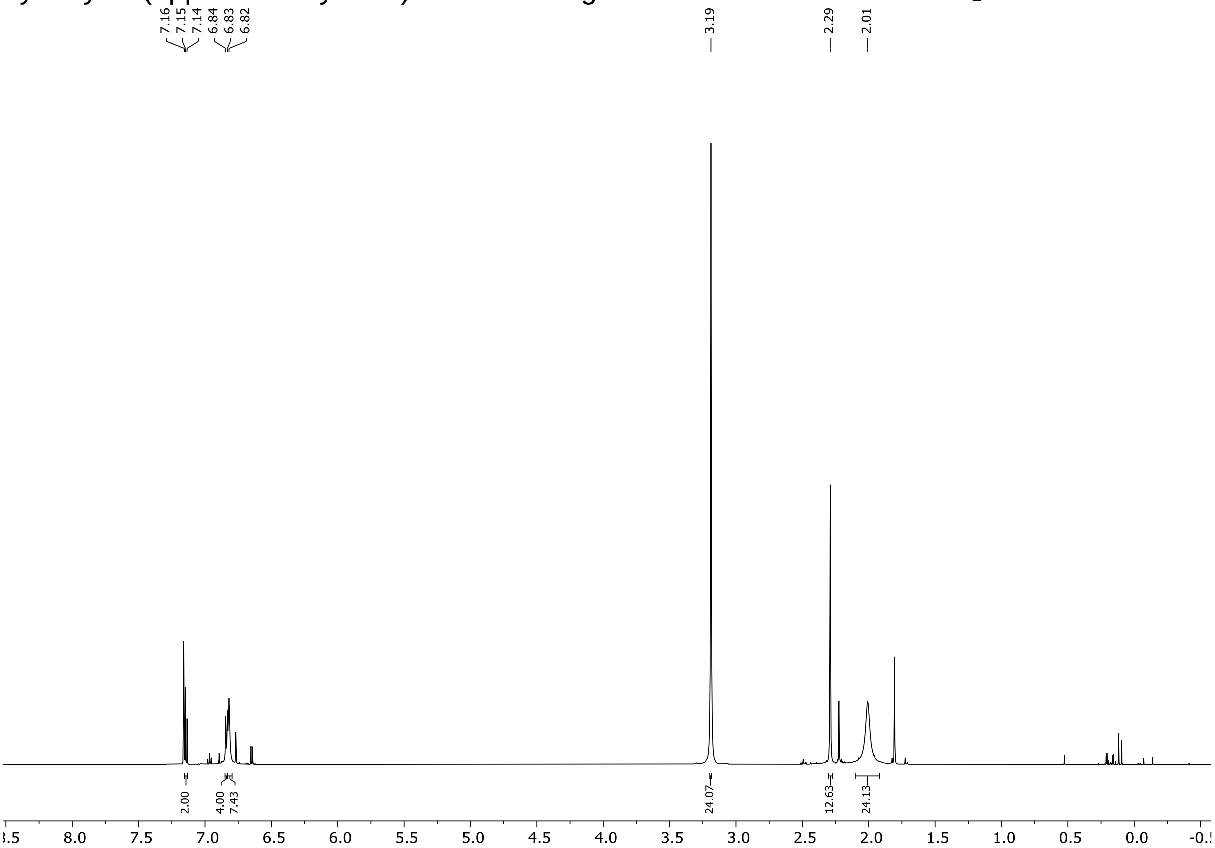

Figure S53. ^{1}H NMR spectrum of [^{Mes}Ter$_2$B-μ-O_2C=O][K(18c6)] (**7a**) (400 MHz, C_6D_6, 298 K).

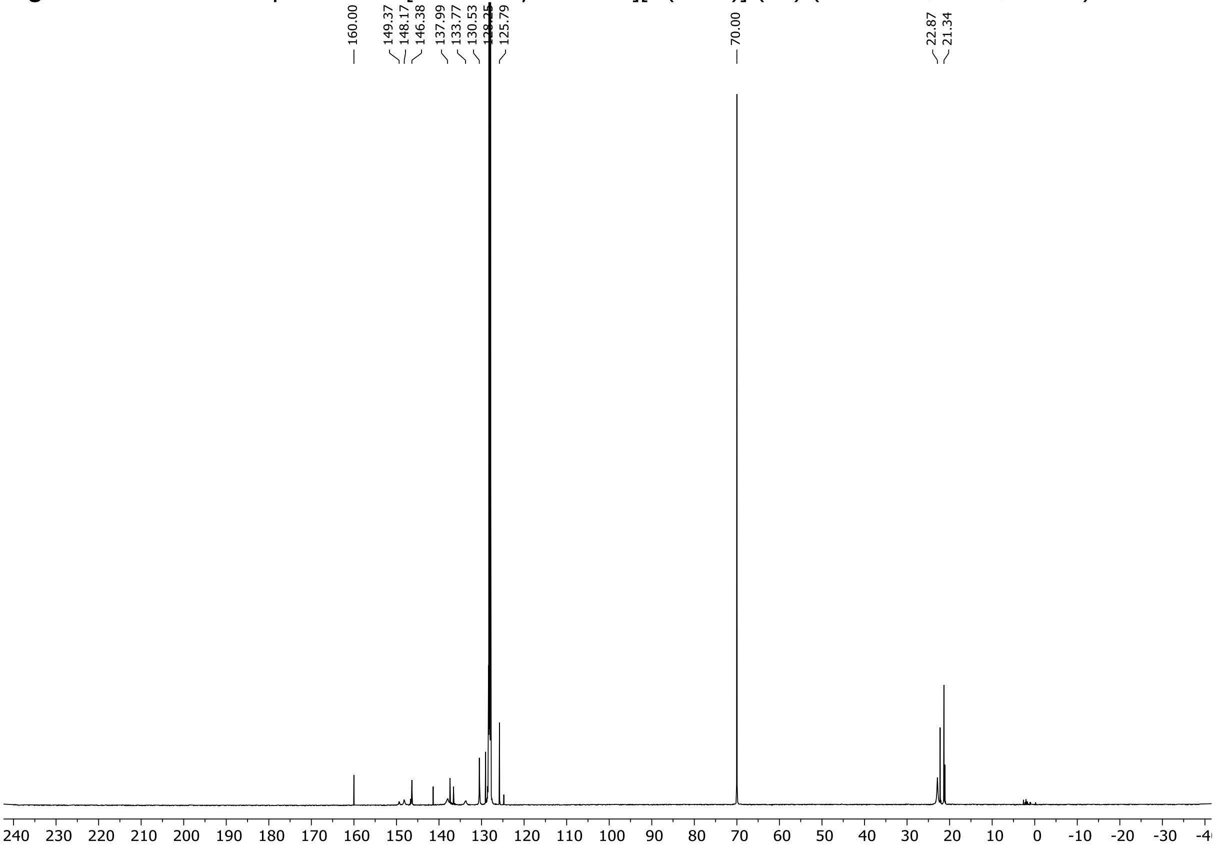

Figure S54. ^{13}C{^{1}H} NMR spectrum of [^{Mes}Ter$_2$B-μ-O_2C=O][K(18c6)] (**7a**) (126 MHz, C_6D_6, 298 K).

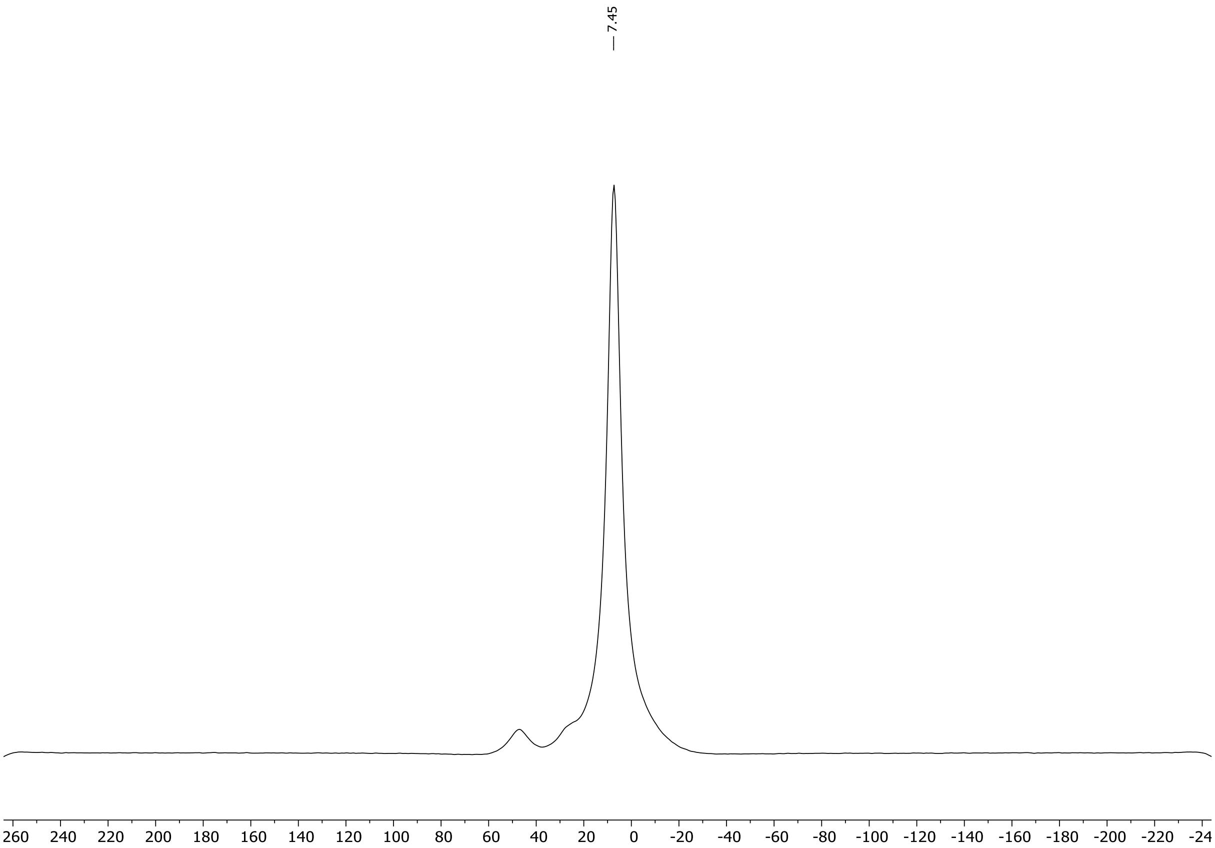

Figure S55. $^{11}B\{^{1}H\}$ NMR spectrum of [$^{Mes}Ter_2B$-μ-O_2C=O][K(18c6)] (**7a**) (96 MHz, C_6D_6, 298 K).

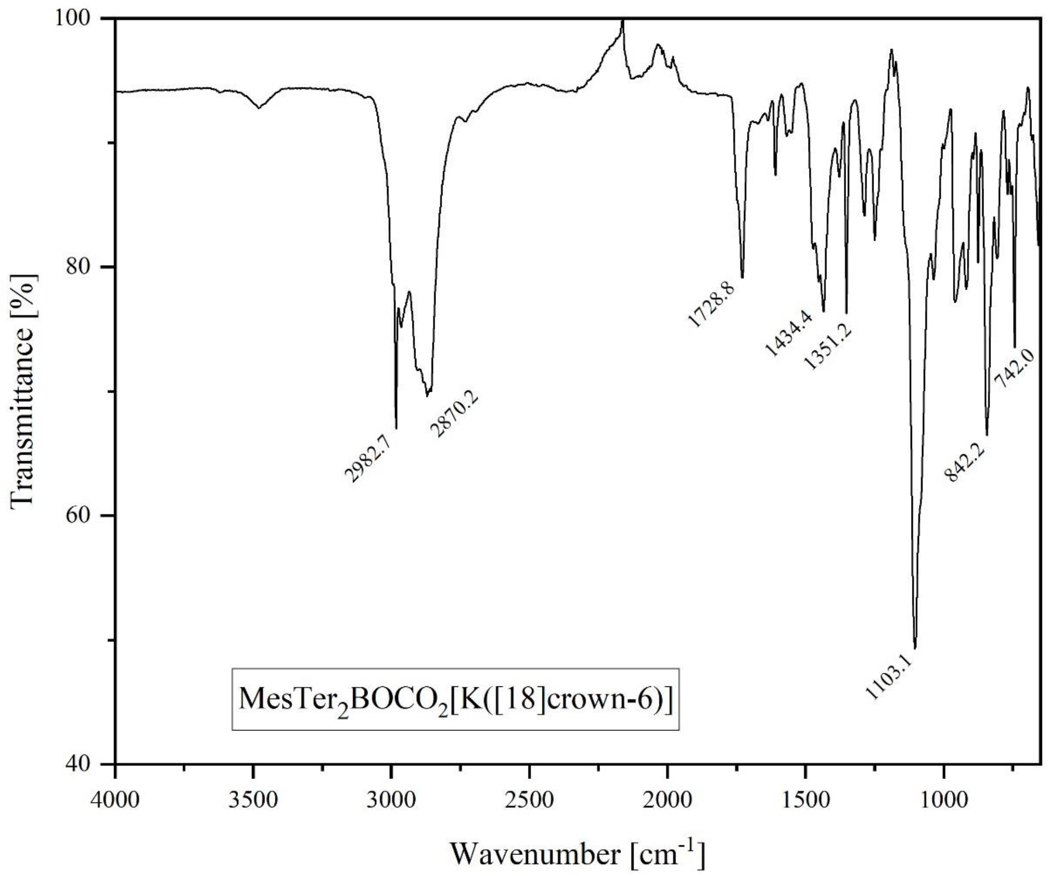

Figure S56. IR spectrum of [^{Mes}Ter$_2$B-μ-O$_2$C=O][K(18c6)] (**7a**).
IR (ATR): ṽ [cm^{-1}] = 2983 (s), 2964 (m), 2903 (s), 2870 (s), 1729 (m), 1609 (w), 1567 (w), 1551 (w), 1471 (m), 1452 (m), 1434 (m), 1378 (w), 1351 (m), 1287 (m), 1248 (m), 1103 (s), 1036 (m), 998 (w), 957 (m), 917 (m), 874 (m), 842 (s), 806 (m), 768 (w), 742 (m), 657 (m).

Synthesis of [^{Mes}Ter$_2$B-μ-O$_2$C=O][K(18c6)] (7b)

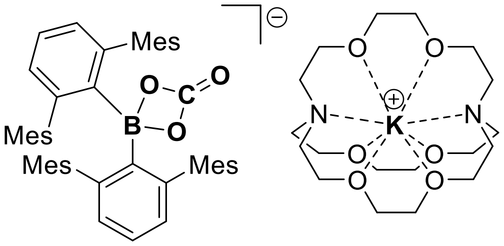

[(^{Mes}Ter)$_2$BO][K(2.2.2-crypt)] (0.030 g, 0.028 mmol) was dissolved in 0.5 mL of THF-d_8 and transferred to a J. Young NMR tube. The solution underwent three cycles of freeze-pump-thaw degassing, after which 1 bar of CO_2 was introduced. The reaction mixture was analyzed by multinuclear NMR spectroscopy, revealing the predominant formation of [^{Mes}Ter$_2$B-μ-O$_2$C=O][K(2.2.2-crypt)] (**7b**), with minor amounts of ^{Mes}Ter$_2$BOH (**2**), likely due to slight hydrolysis. All volatile components were then removed under vacuum to give crude **7b** as a colourless solid.
Crystals of **7b** suitable for single crystal X-ray diffraction were obtained by slow diffusion of *n*-hexane into a solution of **7b** in THF at -30 °C.

Yield: 0.031 g (0.028 mmol; 98%).
^{1}H NMR (400 MHz, THF-d_8, 298 K): δ = 1.59 (s(br), 24H, *o*-C*H*$_3$-C$_6$H$_3$), 2.16 (s, 12H, *p*-C*H*$_3$-C$_6$H$_3$), 2.54-2.55 (m, 12H, CH$_2$), 3.51-3.53 (m, 12H, CH$_2$), 3.56 (s, 12H, CH$_2$), 6.32-6.33 (m(br), 4H, *m*-CH$_{Aryl}$B), 6.48 (s, 8H, *m*-C*H*$_{Aryl}$-C$_6$H$_3$), 6.82 (t, $^3J_{H,H}$ = 7.4 Hz, 2H, *p*-CH$_{Aryl}$B) ppm.
^{13}C{^{1}H} NMR (126 MHz, THF-d_8, 298 K): δ = 21.1 (*p*-$\underline{C}$H$_3$-C$_6$H$_3$), 22.9 (br, *o*-$\underline{C}$H$_3$-C$_6$H$_3$), 54.7 (CH$_2$), 68.4 (CH$_2$), 71.2 (CH$_2$), 124.7 (CH$_{Aryl}$), 128.1 (br, CH$_{Aryl}$), 129.9 (CH$_{Aryl}$), 133.8 (C$_{q,Aryl}$), 137.2 (C$_{q,Aryl}$), 146.1 (C$_{q,Aryl}$), 148.3 (br, C$_{q,Aryl}$) 151.5 (br, C$_{q,Aryl}$), 157.4 (O$_2$C=O) ppm.
^{11}B/^{11}B{^{1}H} NMR (96 MHz, THF-d_8, 298 K): δ = 5.7 ppm.
HR/MS: m/z calcd.: 697.3858 [$C_{49}H_{50}BO_3$]$^-$ measured (ESI, negative): not detected.
HR/MS: m/z calcd.: 415.2205 [$C_{18}H_{36}N_2O_6K$]$^+$ measured (ESI, positive): m/z 415.2191.

Note: The additional signals in the NMR spectra mainly correspond to (^{Mes}Ter)$_2$BOH due to slight hydrolysis (approximately 10%) of the starting material after addition of CO_2.

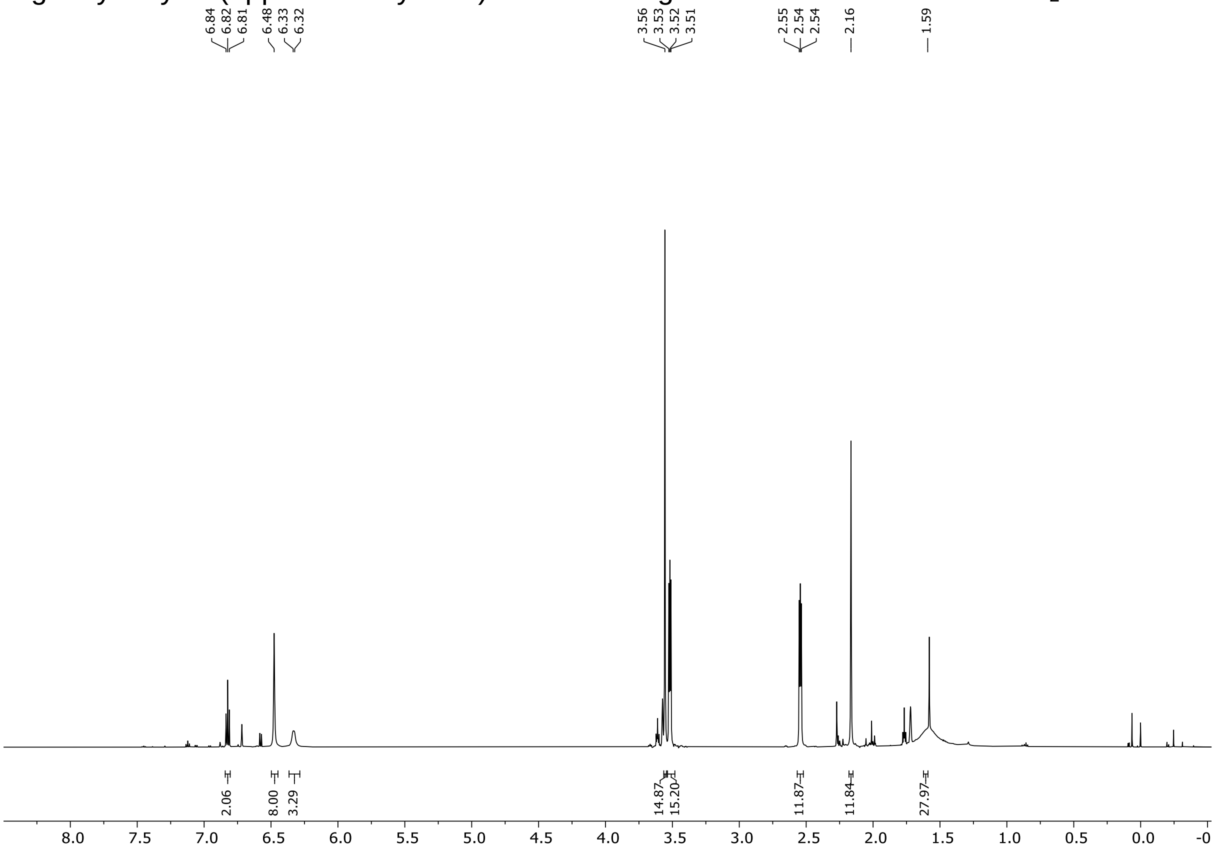

Figure S57. ^{1}H NMR spectrum of [^{Mes}Ter$_2$B-μ-O_2C=O][K(2.2.2-crypt)] (**7b**) (400 MHz, THF-d_8, 298 K).

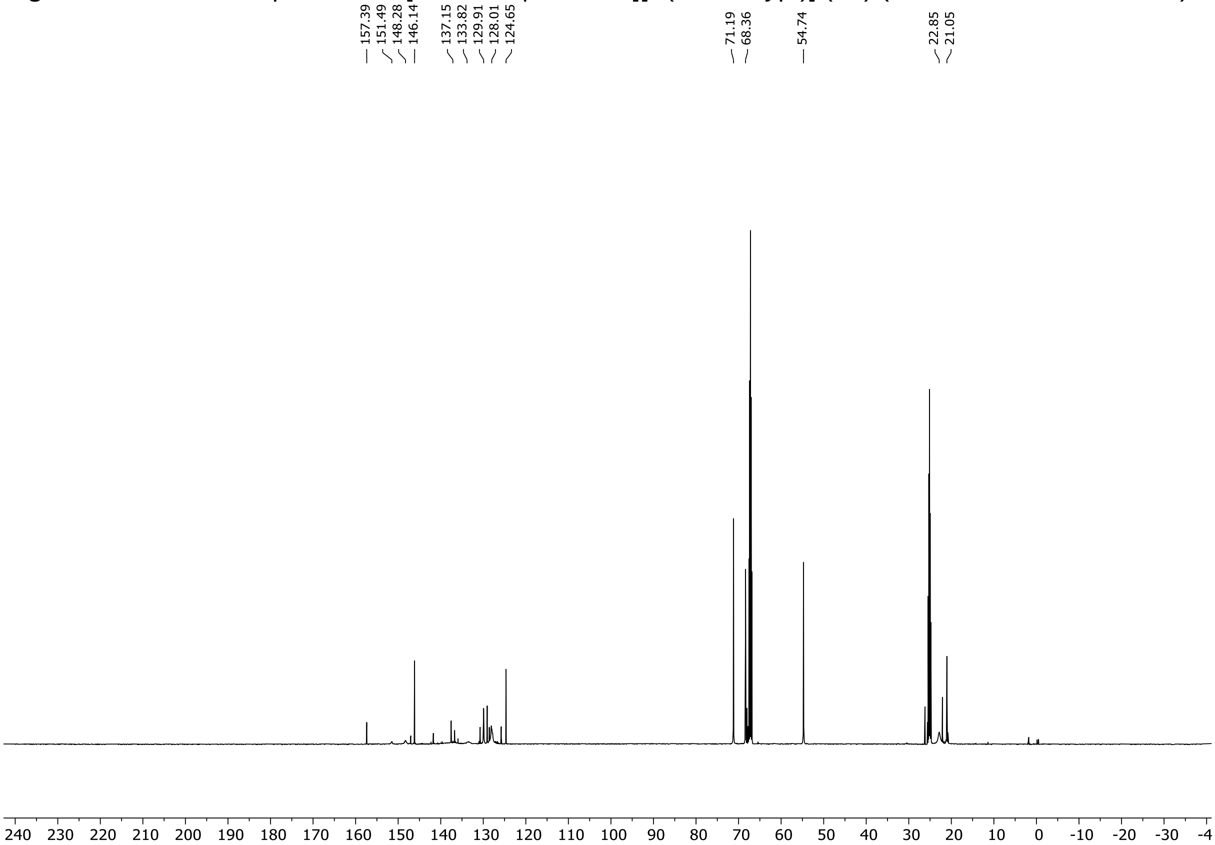

Figure S58. ^{13}C{^{1}H} NMR spectrum of [^{Mes}Ter$_2$B-μ-O_2C=O][K(2.2.2-crypt)] (**7b**) (126 MHz, THF-d_8, 298 K).

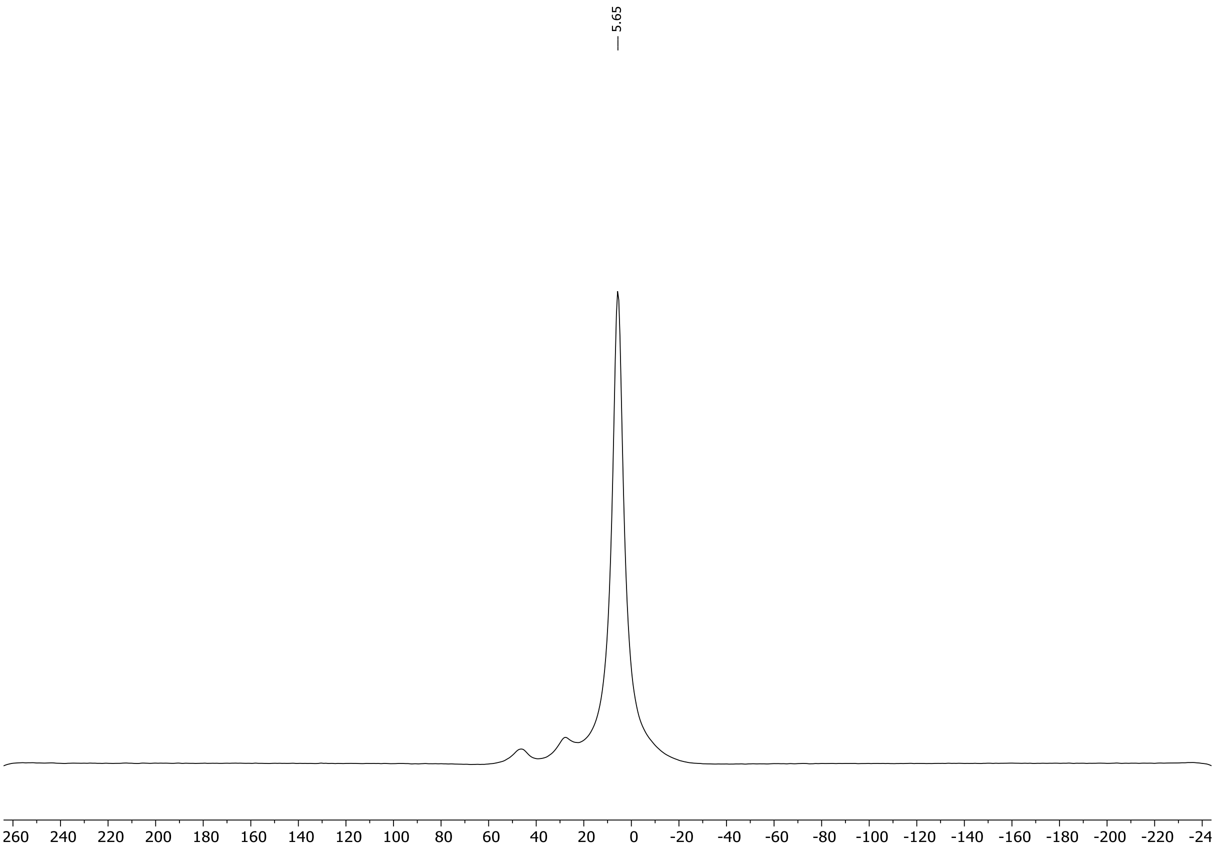

Figure S59. $^{11}B\{^{1}H\}$ NMR spectrum of [$^{Mes}Ter_2B$-μ-O_2C=O][K(2.2.2-crypt)] (**7b**) (96 MHz, THF-d_8, 298 K).

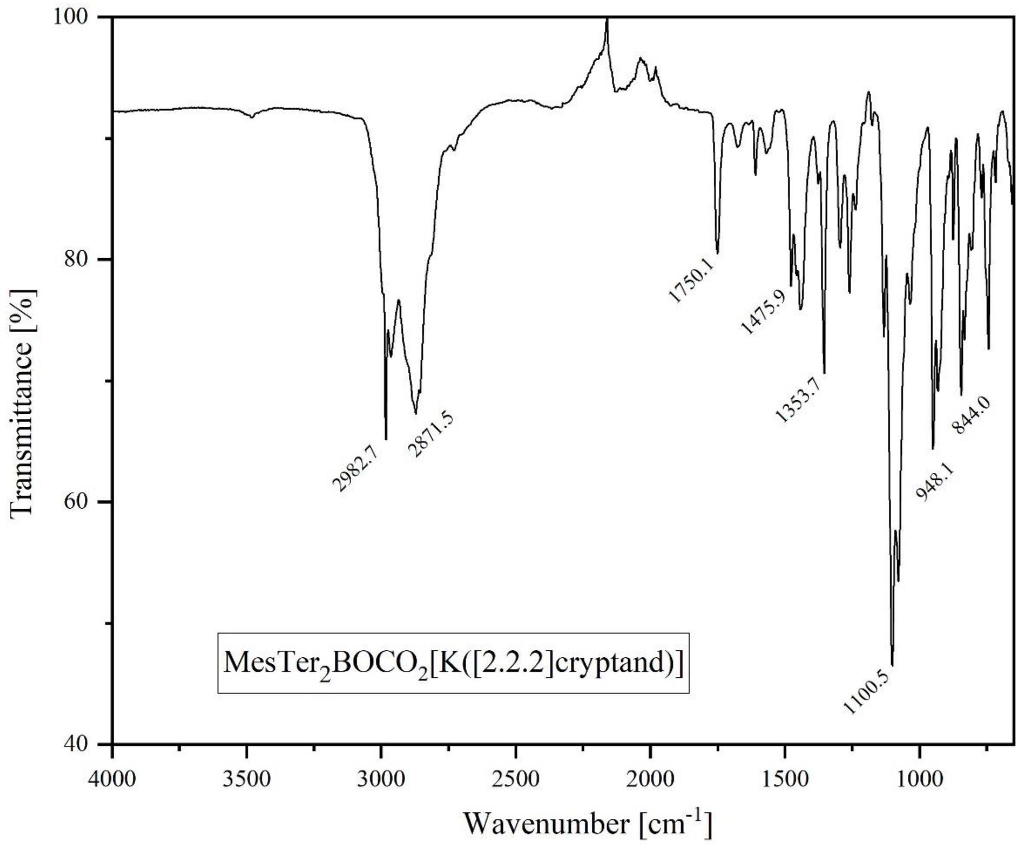

Figure S60. IR spectrum of [^{Mes}Ter$_2$B-μ-O$_2$C=O][K(2.2.2-crypt)] (**7b**).
IR (ATR): ṽ [cm^{-1}] = 2983 (s), 2964 (m), 2872 (s), 1750 (m), 1674 (w), 1609 (w), 1568 (w), 1476 (m), 1441 (m), 1375 (w), 1354 (s), 1294 (m), 1259 (m), 1237 (w), 1131 (m), 1101 (s), 1078 (s), 1034 (m), 948 (s), 929 (s), 875 (m), 844 (s), 832 (m), 805 (m), 768 (w), 743 (m), 717 (w), 655 (w).

Reactions of [(^{Mes}Ter)$_2$BO][HIMe$_4$] (3a) and [(^{Mes}Ter)$_2$BO][HI^{i}Pr$_2$Me$_2$] (3b) with CS$_2$ to give (^{Mes}Ter)$_2$BOH (2) and S$_2$CIMe$_4$ or S$_2$CI^{i}Pr$_2$Me$_2$

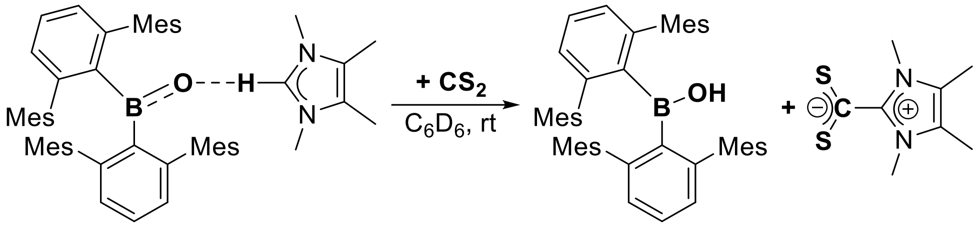

[(^{Mes}Ter)$_2$BO][HIMe$_4$] (**3a**) (0.025 g, 0.032 mmol) was dissolved in 0.5 mL of C_6D_6, followed by the addition of CS_2 (one drop with 1 mL syringe). Subsequent analysis by multinuclear NMR spectroscopy revealed the formation of (^{Mes}Ter)$_2$BOH (**2**) and S$_2$CIMe$_4$ (Figure S61).

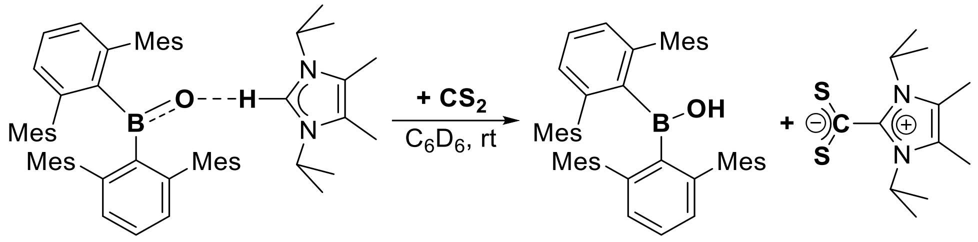

[(^{Mes}Ter)$_2$BO][HI^{i}Pr$_2$Me$_2$] (**3b**) (0.025 g, 0.030 mmol) was dissolved in 0.5 mL of C_6D_6, followed by the addition of CS_2 (one drop with 1 mL syringe). Subsequent analysis by multinuclear NMR spectroscopy revealed the formation of (^{Mes}Ter)$_2$BOH (**2**) and S$_2$CI^{i}Pr$_2$Me$_2$ (Figure S62). The latter precipitates from the solution as a red solid. Heating the solution to 90 °C for one hour, followed by slow cooling to room temperature, leads to the formation of red crystals of S$_2$CI^{i}Pr$_2$Me$_2$ suitable for single crystal X-ray diffraction. The obtained unit cell was consistent with the literature.[S4]

Characteristic ^{1}H NMR data of S$_2$CIMe$_4$:
^{1}H NMR (400 MHz, C_6D_6, 298 K): δ = 0.99 (s, 6H, C$_q$<u>C</u>H$_3$), 2.90 (s, 6H, NCH$_3$) ppm.

Characteristic ^{1}H NMR data of S$_2$CI^{i}Pr$_2$Me$_2$:
^{1}H NMR (400 MHz, C_6D_6, 298 K): δ = 1.00 (d, $^3J_{H,H}$ = 7.1Hz, 12H, CH(C*H*$_3$)$_2$), 1.38 (s, 6H, C$_q$CH$_3$), 4.96 (hept, $^3J_{H,H}$ = 6.9 Hz, 2H, C*H*(CH$_3$)$_2$) ppm.

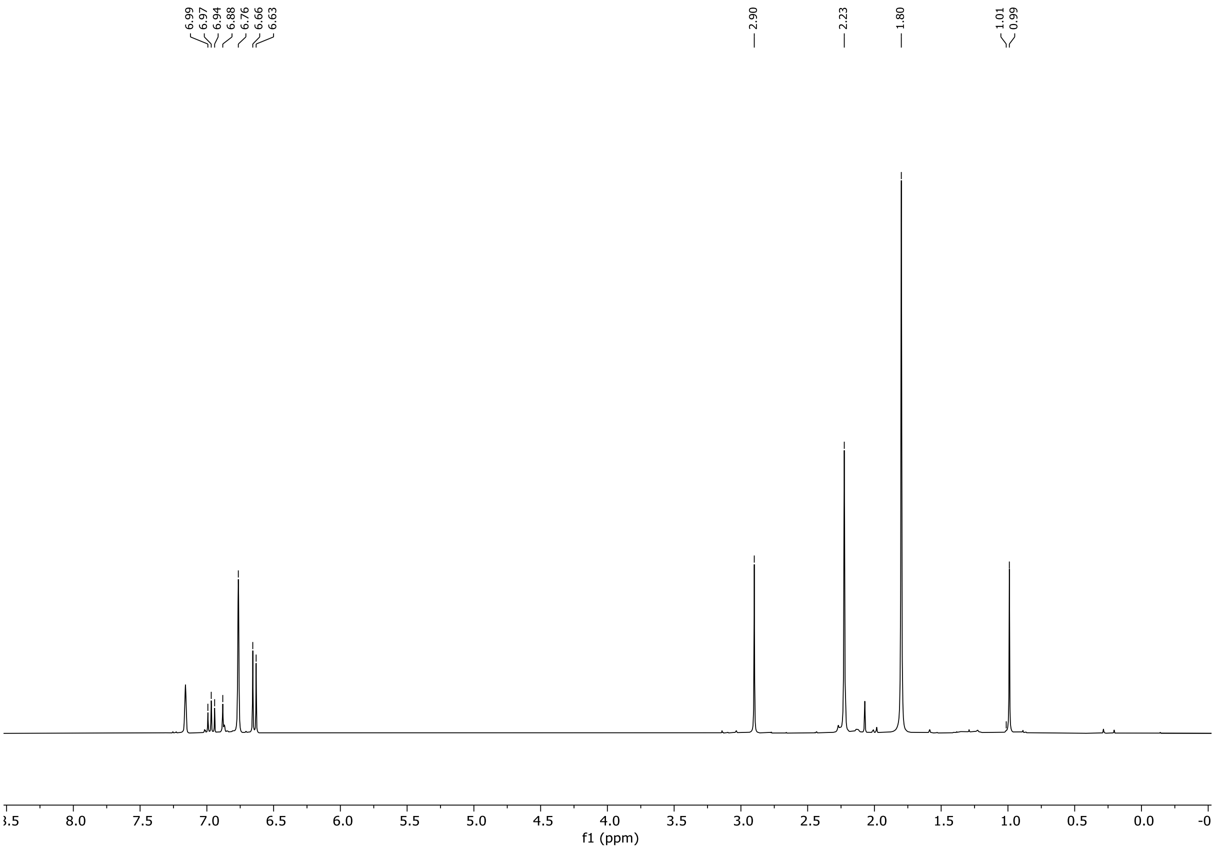

Figure S61. ^{1}H NMR spectrum obtained after the reaction of [(^{Mes}Ter)$_2$BO][HIMe$_4$] (**3a**) with CS_2 to give ^{Mes}Ter$_2$BOH (**2**) and S_2CIMe$_4$ (400 MHz, C_6D_6, 298 K).

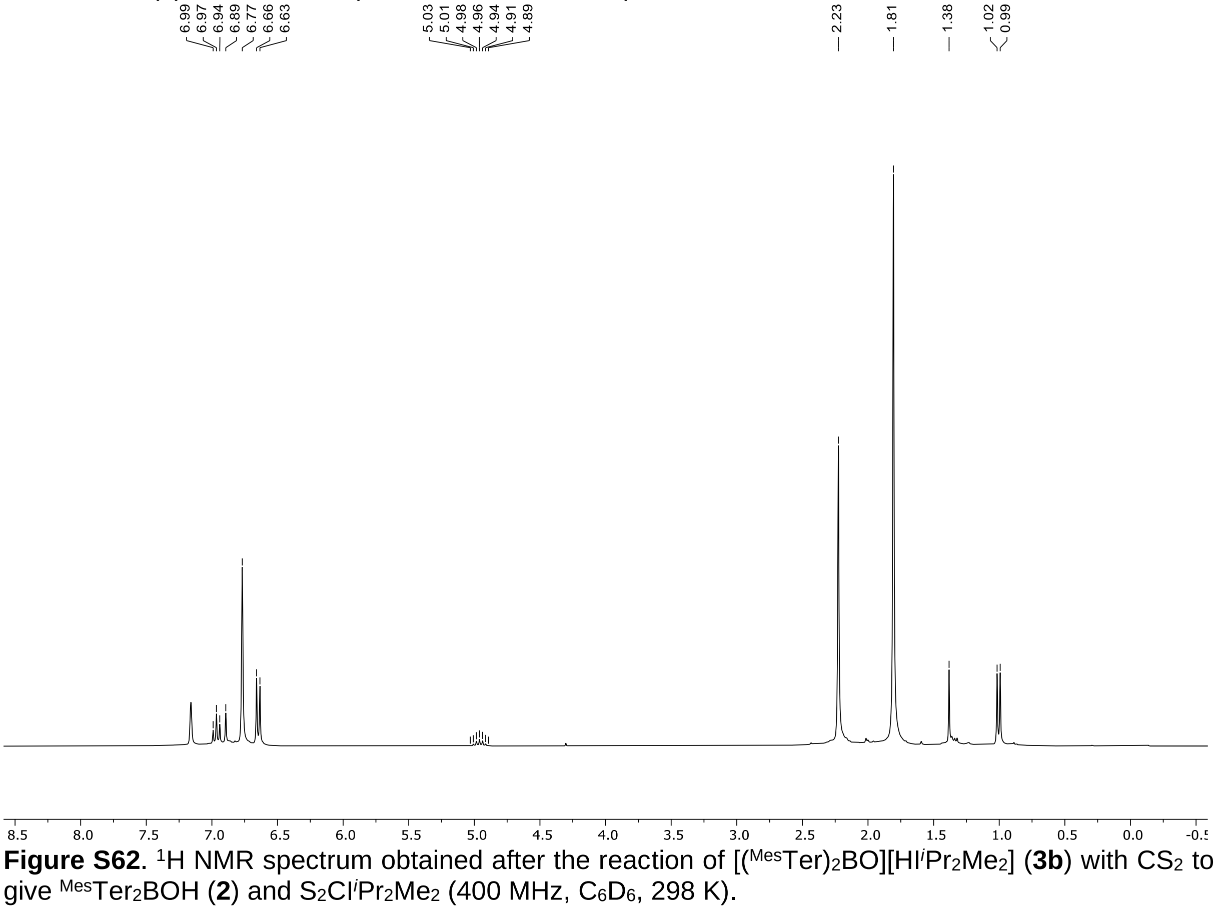

Figure S62. ^{1}H NMR spectrum obtained after the reaction of [(^{Mes}Ter)$_2$BO][HI^{i}Pr$_2$Me$_2$] (**3b**) with CS_2 to give ^{Mes}Ter$_2$BOH (**2**) and S_2CI^{i}Pr$_2$Me$_2$ (400 MHz, C_6D_6, 298 K).

Synthesis of [(^{Mes}Ter)$_2$BO][^{n}Bu$_4$N] (5c) and its degradation to yield (^{Mes}Ter)$_2$BOH (2), tri-*n*-butylamine and but-1-ene

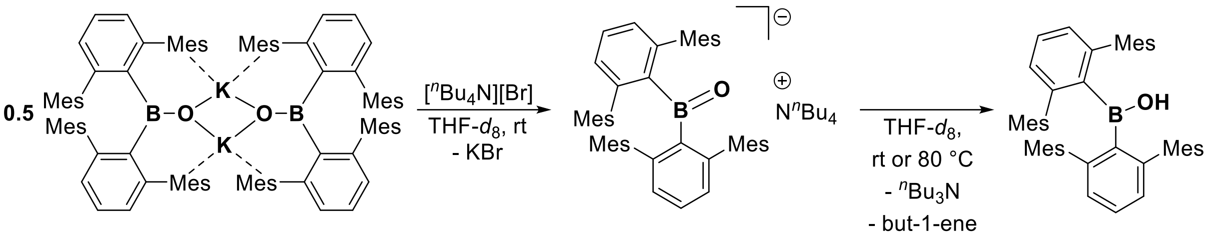

[(^{Mes}Ter)$_2$BOK]$_2$ (**4**) was prepared *in situ* by reacting (^{Mes}Ter)$_2$BOH (**2**) (0.020 g, 0.031 mmol) with K{N(SiMe$_3$)$_2$} (0.006 g, 0.031 mmol) in 0.5 mL of benzene at room temperature, stirring for 10 minutes. All volatile components were then removed under vacuum, and the remaining solid was redissolved in 0.5 mL of THF-d_8, followed by the addition of [^{n}Bu$_4$N][Br] (0.010 g, 0.031 mmol). The reaction mixture was stirred for another 10 minutes at room temperature and then filtered to remove the precipitate. Subsequent analysis by ^{1}H NMR spectroscopy revealed the formation of [(^{Mes}Ter)$_2$BO][^{n}Bu$_4$N] (**5c**) (Figure S63). **5c** was found to be unstable in solution, decomposing into (^{Mes}Ter)$_2$BOH (**2**), tri-*n*-butylamine, and but-1-ene. Heating the reaction mixture to 80 °C for five hours led to complete conversion. Subsequent ^{13}C{^{1}H} NMR analysis, along with comparison to literature data, clearly supported the formation of these by-products (*vide supra*) (Figures S64–S66, Table S1).[S5-S7]

^{1}H NMR data of [(^{Mes}Ter)$_2$BO][^{n}Bu$_4$N] (**5c**):
^{1}H NMR (400 MHz, THF-d_8, 298 K): δ = 1.00 (t, $^3J_{H,H}$ = 7.4 Hz, 12H, N(CH$_2$CH$_2$CH$_2$C*H*$_3$)$_4$), 1.42 (m, 8H, N(CH$_2$CH$_2$C*H*$_2$CH$_3$)$_4$), 1.69 (s, 24H, *o*-C*H*$_3$-C$_6$H$_3$), 1.73 (m, 8H, N(CH$_2$C*H*$_2$CH$_2$CH$_3$)$_4$), 2.23 (*p*-C*H*$_3$-C$_6$H$_3$), 3.44 (m, 8H, N(C*H*$_2$CH$_2$CH$_2$CH$_3$)$_4$), 6.29 (d, $^3J_{H,H}$ = 7.5 Hz, 4H, *m*-CH$_{Aryl}$-B), 6.63 (s, 8H, *m*-C*H*$_{Aryl}$-C$_6$H$_3$), 6.75 (t, $^3J_{H,H}$ = 7.5 Hz, *p*-CH$_{Aryl}$-B) ppm.

NMR data obtained after full degradation of [(^{Mes}Ter)$_2$BO][^{n}Bu$_4$N] (**5c**):
^{1}H NMR (400 MHz, THF-d_8, 298 K):
2: δ = 1.59 (s, 24H, *o*-C*H*$_3$-C$_6$H$_3$), 2.28 (s, 12H, *p*-C*H*$_3$-C$_6$H$_3$), 6.58 (d, $^3J_{H,H}$ = 7.6 Hz, 4H, *m*-CH$_{Aryl}$-B), 6.73 (s, 8H, *m*-C*H*$_{Aryl}$-C$_6$H$_3$), 6.78 (s, 1H, BOH), 7.13 (t, $^3J_{H,H}$ = 7.6 Hz, 2H, *p*-CH$_{Aryl}$-B) ppm.
Tri-*n*-butylamine: δ = 0.90 (t, $^3J_{H,H}$ = 7.2 Hz, 9H, N(CH$_2$CH$_2$CH$_2$C*H*$_3$)$_3$), 1.32 (m, 6H, N(CH$_2$CH$_2$ C*H*$_2$CH$_3$)$_3$), 1.40 (m, 6H, N(CH$_2$C*H*$_2$CH$_2$CH$_3$)$_3$), 2.35 (t, $^3J_{H,H}$ = 7.1 Hz, 6H, N(C*H*$_2$CH$_2$CH$_2$CH$_3$)$_3$) ppm.
But-1-ene: δ = 0.98 (t, $^3J_{H,H}$ = 7.5 Hz, 3H, CH$_3$), 4.88 (ddt, $^3J_{H,H}$ = 10.2 Hz, $^2J_{H,H}$ = 2.1 Hz, $^4J_{H,H}$ = 1.4 Hz, 1H, H$_2$C–C(H)=C*H*$_2$), 4.97 (ddt, $^3J_{H,H}$ = 17.1 Hz, $^2J_{H,H}$ = 2.1 Hz, $^4J_{H,H}$ = 1.7 Hz, 1H, H$_2$C–C(H)=C*H*$_2$), 5.84 (ddt, $^3J_{H,H}$ = 16.7 Hz, $^3J_{H,H}$ = 10.2 Hz, $^3J_{H,H}$ = 6.3 Hz, 1H, H$_2$C–C(*H*)=CH$_2$) ppm.
Note: One ^{1}H NMR signal of but-1-ene could not be clearly assigned. Based on 2D NMR spectra, it might be overalapped by other signals in the range from δ^{1}H = 1.9 to 2.0 ppm.
^{13}C{^{1}H} NMR (126 MHz, THF-d_8, 298 K):
2: 21.0 (*p*-<u>C</u>H$_3$-C$_6$H$_3$), 22.1 (*o*-<u>C</u>H$_3$-C$_6$H$_3$), 128.6 (*p*-CH$_{Aryl}$-B), 129.1 (*m*-<u>C</u>H$_{Aryl}$-C$_6$H$_3$), 130.7 (*m*-CH$_{Aryl}$-B), 136.7 (C$_{q,Aryl}$), 137.6 (C$_{q,Aryl}$), 140.0 (br, C$_{q,Aryl}$B), 141.8 (C$_{q,Aryl}$), 147.1 (C$_{q,Aryl}$) ppm.
Tri-*n*-butylamine: δ = 14.0 (N(CH$_2$CH$_2$CH$_2$<u>C</u>H$_3$)$_3$), 21.2 (N(CH$_2$CH$_2$<u>C</u>H$_2$CH$_3$)$_3$), 30.6 (N(CH$_2$<u>C</u>H$_2$CH$_2$CH$_3$)$_3$), 54.7 (N(<u>C</u>H$_2$CH$_2$CH$_2$CH$_3$)$_3$) ppm.
But-1-ene: δ = 13.3 (CH$_3$), 27.3 (H$_2$<u>C</u>–C(H)=CH$_2$), 113.4 (H$_2$C–C(H)=<u>C</u>H$_2$), 140.9 (H$_2$C–<u>C</u>(H)=CH$_2$) ppm.

Table S1. Comparison of experimental obtained $^{13}C\{^{1}H\}$ NMR chemical shifts to literature values of tetra-*n*-butylammonium bromide (TBAB), tri-*n*-butylamine (TBA) and but-1-ene.

Compound		Solvent	$\delta^{13}C\{^{1}H\}$ [ppm]				Reference
			1	2	3	4	
TBAB	(exp.)	THF-d_8	59.5	25.0	20.5	14.3	
TBAB	(lit.)	$CDCl_3$	58.9	24.5	20.1	14.0	[S1]
TBA	(exp.)	THF-d_8	54.7	30.6	21.2	14.0	
TBA	(lit.)	$CDCl_3$	54.0	29.5	20.8	13.3	[S2]
But-1-ene	(exp.)	THF-d_8	113.4	140.9	27.3	13.3	
But-1-ene	(lit.)	$CDCl_3$	113.1	140.7	26.6	12.9	[S3]

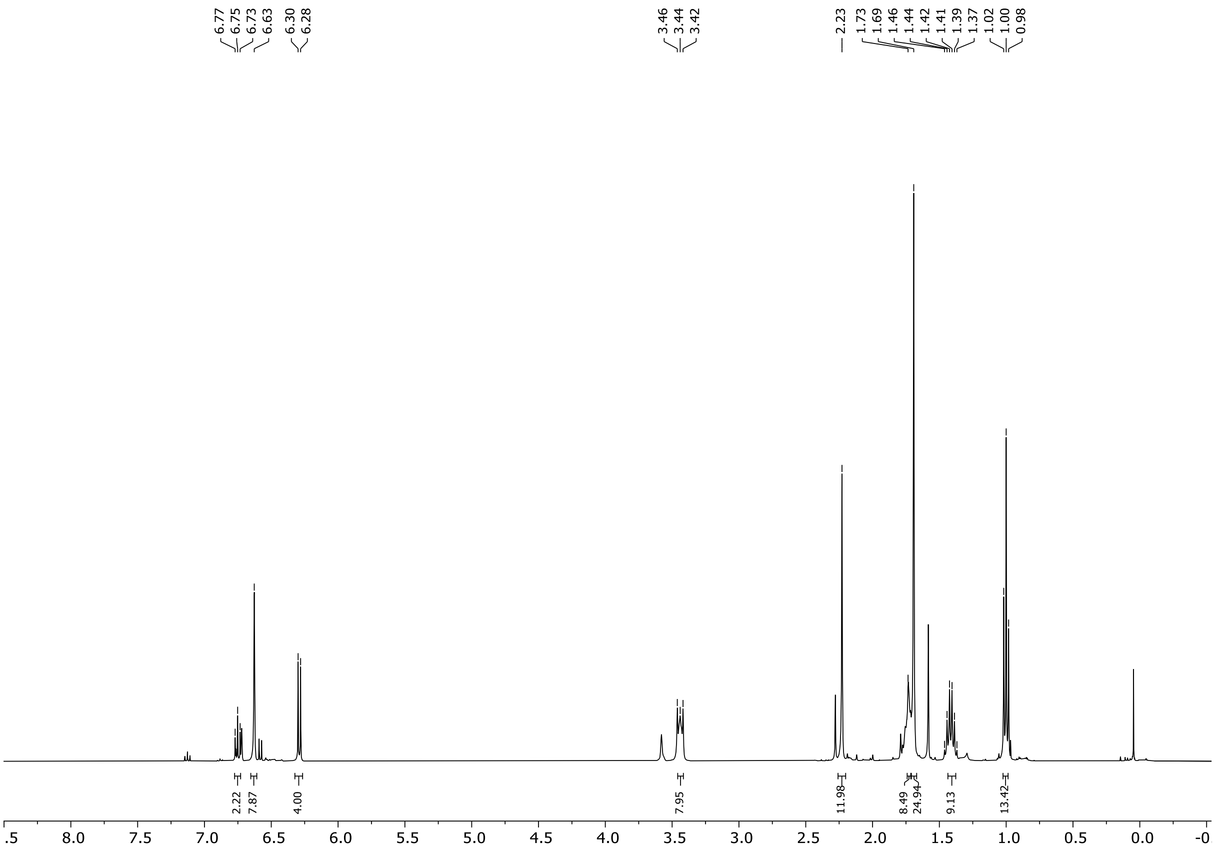

Figure S63. ^{1}H NMR spectrum of [(^{Mes}Ter)$_2$BO][^{n}Bu$_4$N] (**5c**) (400 MHz, THF-d_8, 298 K).

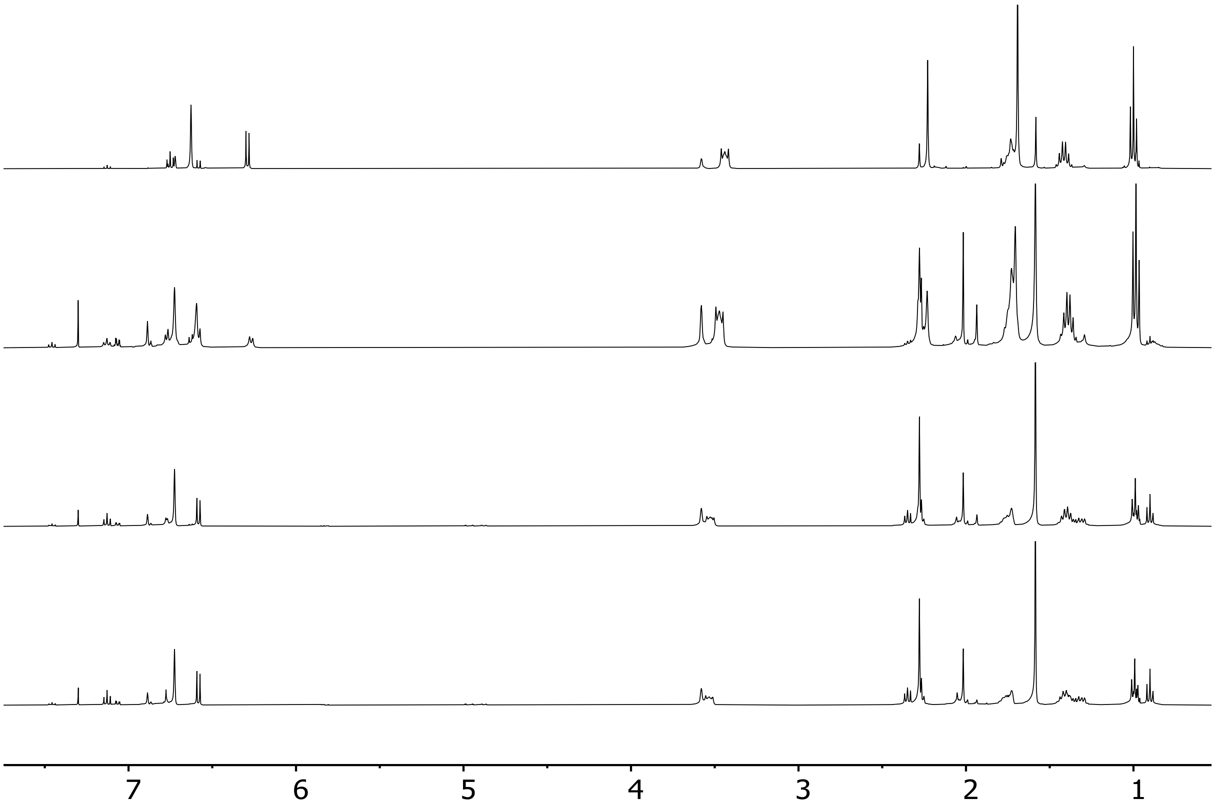

Figure S64. Degradation of [(^{Mes}Ter)$_2$BO][^{n}Bu$_4$N] (**5c**) to give (^{Mes}Ter)$_2$BOH (**2**), tri-*n*-butylamine, and but-1-ene (400 MHz, THF-*d*$_8$, 298 K).

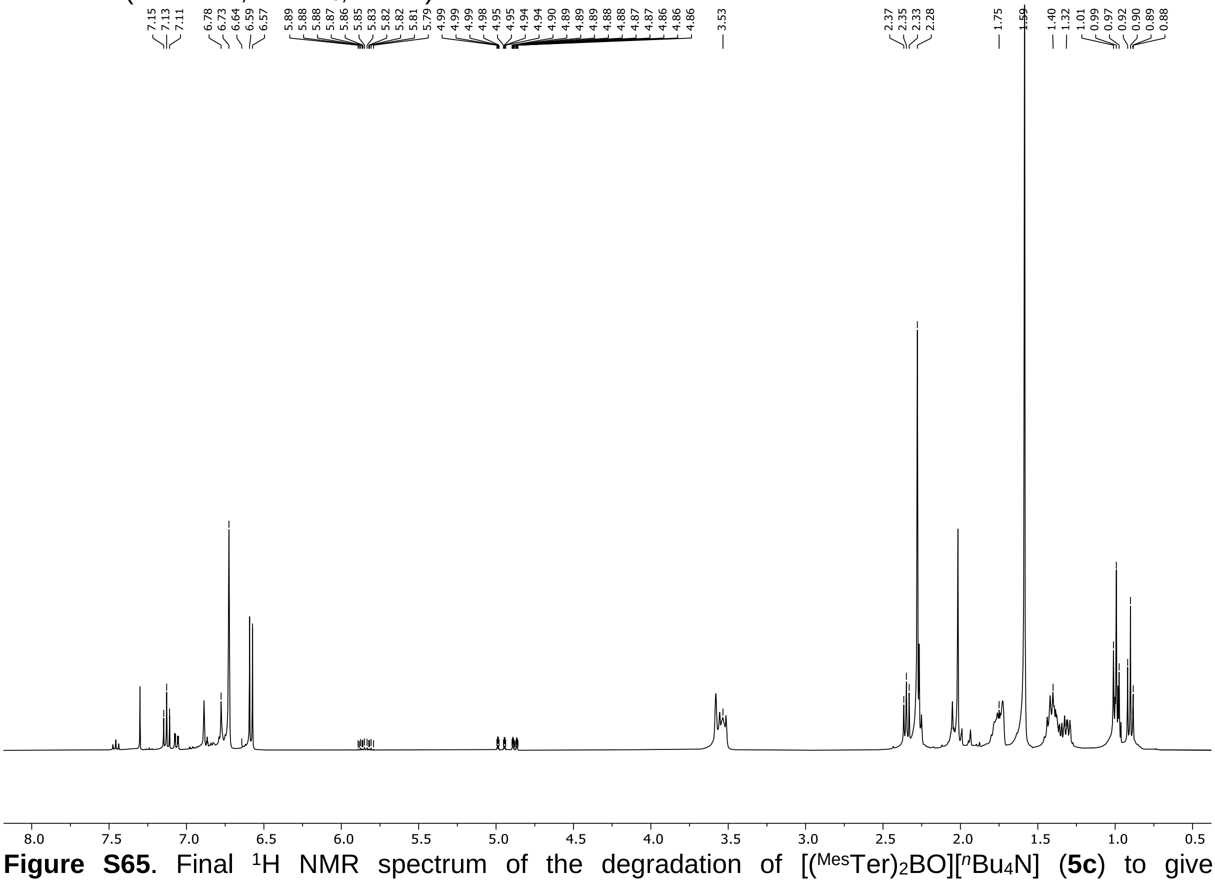

Figure S65. Final ^{1}H NMR spectrum of the degradation of [(^{Mes}Ter)$_2$BO][^{n}Bu$_4$N] (**5c**) to give (^{Mes}Ter)$_2$BOH (**2**), tri-*n*-butylamine, and but-1-ene (400 MHz, THF-*d*$_8$, 298 K).

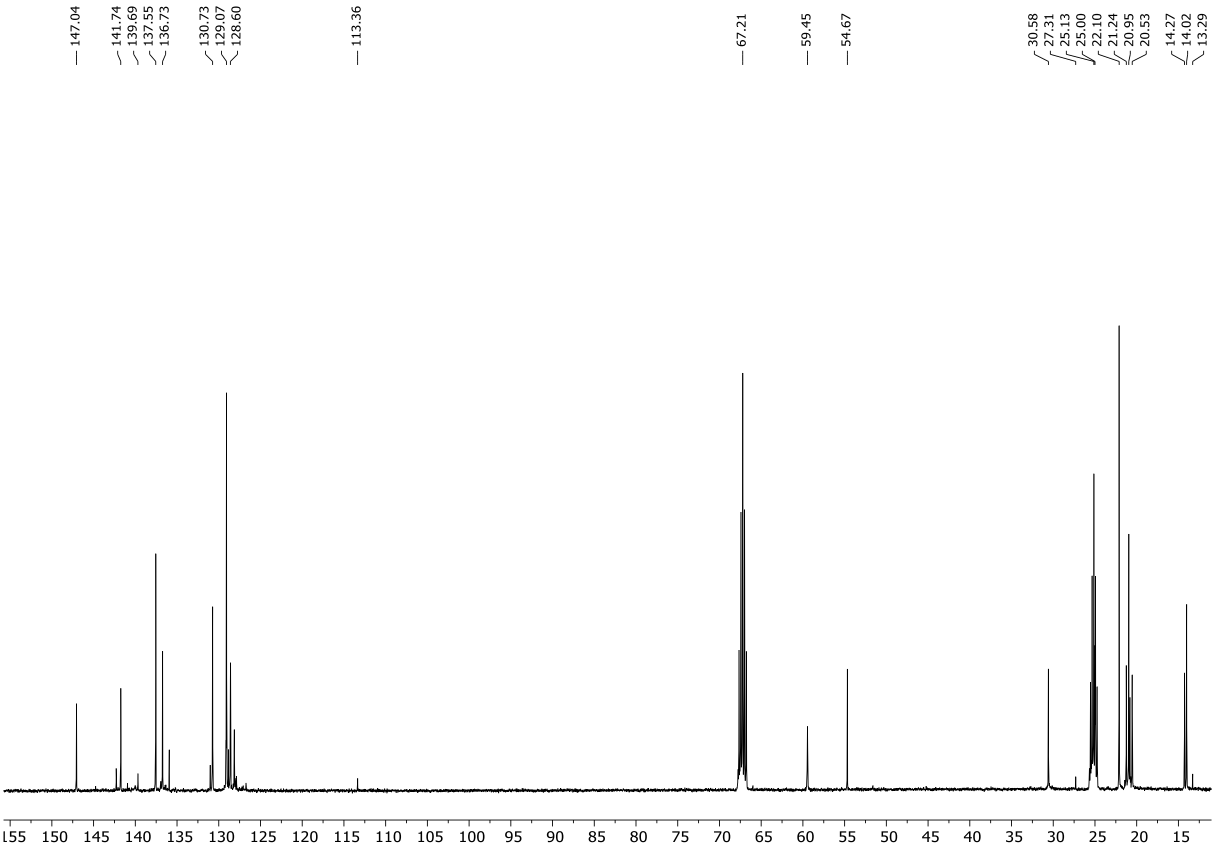

Figure S66. Final $^{13}C\{^{1}H\}$ NMR spectrum of the degradation of $[(^{Mes}Ter)_2BO][^{n}Bu_4N]$ (**5c**) to give $(^{Mes}Ter)_2BOH$ (**2**), tri-*n*-butylamine, and but-1-ene (126 MHz, THF-d_8, 298 K).

Crystallographic Details

Single crystal X-ray data were collected at 100 K using an open flow nitrogen stream on a Bruker Smart Apex diffractometer equipped with a Bruker rotating anode (Mo) Incoatec, Incoatec mirror optics and a Photon III detector. The data were integrated with SAINT[S8]. A multi-scan absorption correction was applied using SADABS[S9]. All structures were solved using the dual-space algorithm in ShelXT[S10] and refined against F^2 with the use of SHELXL[S11] within the OLEX2[S12] program package or in the graphical user interface SelXle[S13]. All non-hydrogen atoms were refined using anisotropic displacement parameters. Unless noted otherwise, hydrogen atoms were refined using a riding model with their U_{iso} values constrained to 1.5 U_{eq} of their pivot atoms for terminal sp^3 carbon atoms and 1.2 times for all other carbon atoms.

In an effort to locate the precise and accurate positions of the hydrogen atoms in 2, 3a, and 3b, Hirshfeld atom refinements (HARs) were carried out. The geometries obtained from the independent atom model (IAM) provided the initial model used as input for the subsequent HAR performed using NoSpherA2[S14] as implemented in OLEX2[S12] and Orca5.0[S15] at the r^{2}SCAN/cc-pVTZ level of theory. All H atoms were refined freely and anisotropically within HAR.

Crystallographic data for the structural analyses have been deposited with the Cambridge Crystallographic Data Centre. Copies of this information may be obtained free of charge from The Director, CCDC, 12 Union Road, Cambridge CB2 1EZ, UK (Fax: +44-1223-336033; email: deposit@ccdc.cam.ac.uk or http://www.ccdc.cam.ac.uk).

Table S2. Crystal structure data for compounds **2** (polymorph a), **2** (polymorph b) and **3a**.

	**2** (Polymorph a)	**2** (Polymorph b)	**3a**
CCDC	2393960	2393961	2393962
empirical formula	$C_{48}H_{51}BO$	$C_{48}H_{51}BO$	$C_{55}H_{63}BN_2O$ • 2 $C_4H_{10}O$
formula weight	654.76	654.76	927.20
colour	colourless	colourless	clear colourless
habit	needle	cube	block
cryst. dimens, mm	0.1 x 0.005 x 0.005	0.1 x 0.1 x 0.1	0.33 x 0.26 x 0.13
T, K	100	100	100
crystal system	monoclinic	orthorhombic	monoclinic
space group	$P2_1/n$	*Fdd*2	$P2_1/c$
a, Å	11.1173(5)	20.1745(9)	17.5522(9)
b, Å	20.7691(10)	42.6359(14)	17.1331(9)
c, Å	16.9723(8)	8.4338(3)	18.7592(9)
α, deg	90	90	90
β, deg	106.132(2)	90	101.209(3)
γ, deg	90	90	90
V, Å^3	3764.5(3)	7254.4(5)	5533.7(5)
Z	4	8	4
D_{calc}, g•cm^{-3}	1.155	1.199	1.113
μ, mm^{-1}	0.066	0.069	0.066
radiation	Mo Kα (λ = 0.71073)	Mo Kα (λ = 0.71073)	Mo Kα (λ = 0.71073)
θ range, deg	4.28 – 55.00	2.23 – 33.26	4.26 – 52.82
no. of rflns collected	81304	68953	167334
no. of indep. reflns.	8646	9555	11355
R(int)	0.1126	0.0296	0.0695
[I>2σ(I)]	R1 = 0.0420<br>wR2 = 0.0810	R1 = 0.0165<br>wR2 = 0.0366	R1 = 0.0550<br>wR2 = 0.1338
R indices (all data)	R1 = 0.0852<br>wR2 = 0.0983	R1 = 0.0186<br>wR2 = 0.0375	R1 = 0.0623<br>wR2 = 0.1378
GOF on F^2	1.060	1.055	1.077

Table S3. Crystal structure data for compounds **3b**, **4** and **6a**.

	**3b**	**4**	**6a**
CCDC	2393959	2393964	2410251
empirical formula	$C_{59}H_{71}BN_2O$	0.5 $C_{96}H_{100}B_2K_2O_2$ • CH_2Cl_2	$C_{85}H_{112}BKO_{12}S_3$
formula weight	834.98	777.71	1481.91
colour	yellow	clear colourless	colourless
habit	block	block	block
cryst. dimens, mm	0.76 x 0.41 x 0.32	0.32 x 0,30 x 0.21	0.17 x 0.27 x 0.34
T, K	100	100	100
crystal system	tetragonal	triclinic	monoclinic
space group	$P4_12_12$	$P\bar{1}$	$C2/c$
a, Å	13.0805(4)	10.4964(3)	30.157(3)
b, Å	13.0805(4)	16.6368(4)	14.0275(12)
c, Å	29.2717(17)	24.6976(5)	21.813(2)
α, deg	90	100.5000(10)	90
β, deg	90	96.2900(10)	119.591(2)
γ, deg	90	99.7570(10)	90
V, Å^3	5008.4(4)	4135.17(18)	8024.0(13)
Z	4	4	4
D_{calc}, g•cm^{-3}	1.107	1.249	1.227
μ, mm^{-1}	0.064	0.294	0.204
radiation	Mo Kα (λ = 0.71073)	Mo Kα (λ = 0.71073)	Mo Kα (λ = 0.71073)
θ range, deg	4.18 – 65.18	4.546 – 61.016	4.29 – 57.66
no. of rflns collected	130646	247933	122746
no. of indep. reflns.	9007	25201	10436
R(int)	0.0498	0.0575	0.0520
[I>2σ(I)]	R1 = 0.0437<br>wR2 = 0.1127	R1 = 0.0490<br>wR2 = 0.1233	R1 = 0.0448<br>wR2 = 0.1144
R indices (all data)	R1 = 0.0522<br>wR2 = 0.1193	R1 = 0.0597<br>wR2 = 0.1312	R1 = 0.0546<br>wR2 = 0.1224
GOF on F^2	1.055	1.027	1.026

Table S4. Crystal structure data for compounds **3b**, **4** and **6a**.

	**6a'**	**6b**	**7b**
CCDC	2393965	2393966	2393963
empirical formula	$C_{97}H_{110}BKO_{6.70}S_{2.23}$	$C_{71}H_{94}BKN_2O_7S_3$	$C_{77.22}H_{106.90}BKN_2O_{11.27}$
formula weight	1504.37	1233.57	1293.55
colour	colourless	orange	colourless
habit	block	plate	plate
cryst. dimens, mm	0.26 x 0.12 x 0.11	0.23 x 0.14 x 0.06	0.29 x 0.18 x 0.06
T, K	100	100	100
crystal system	monoclinic	monoclinic	triclinic
space group	*C*2/*c*	$P2_1/c$	$P\bar{1}$
a, Å	30.278(5)	11.886(3)	13.5076(19)
b, Å	15.1358(15)	25.172(7)	15.714(2)
c, Å	21.561(4)	22.792(5)	18.428(3)
α, deg	90	90	82.550(9)
β, deg	122.821(3)	100.263(7)	87.627(9)
γ, deg	90	90	68.693(7)
V, $Å^3$	8304(2)	6710(3)	3613.2(9)
Z	4	4	2
D_{calc}, g•cm^{-3}	1.203	1.221	1.189
μ, mm^{-1}	0.176	0.226	1.120
radiation	Mo Kα (λ = 0.71073)	Mo Kα (λ = 0.71073)	Cu Kα (λ = 1.54178)
θ range, deg	3.20 – 61.08	3.63 – 56.59	4.84 – 150.58
no. of rflns collected	140803	136803	73103
no. of indep. reflns.	12686	16659	14737
R(int)	0.0448	0.0786	0.0989
[I>2σ(I)]	R1 = 0.0430<br>wR2 = 0.0996	R1 = 0.0471<br>wR2 = 0.1166	R1 = 0.0881<br>wR2 = 0.2486
R indices (all data)	R1 = 0.0590<br>wR2 = 0.1095	R1 = 0.0765<br>wR2 = 0.1283	R1 = 0.1275<br>wR2 = 0.2912
GOF on F^2	1.037	1.110	1.018

Refinement details:
6a: The crystals seem to crack during shock freezing to 100 K but still yield a reasonable reflection pattern, showing no signs of twinning or significant multicrystallinity.

6a': The compound was synthesized from [$^{Mes}Ter_2BO$][K(18c6)] (**5a**) and CS_2. Hence, the sum formula should accommodate for a ($S_{2-x}O_x$)C=($S_{1-x}O_x$) anion coordinated to the boron atom. However, fixing the refined occupancy parameter with SUMP does lead to a significantly poorer model. This might be an issue with the special position, but the problem could not be solved with negative parts for the disordered fragment. For the final model, unrestrained occupancy parameters were used. Based on this, it can not be ruled out that there was further S/O scrambling during the reaction. Hence, this is believed to be the best solution at this time.

7b: This compound was found to crystallize rather poorly, and several attempts yielded only this as the best result. The material consists of thin plates that appear multi-crystalline under a stereomicroscope with a polarizer. The crystal used for data collection shows some reflections belonging to minor overgrowths, but twin integration gave ratios of 2% or less, so these were ignored. Using Cu radiation, the reflections are generally well separated from the other domains on the frames. The main issue here is simply the poor scattering power of the sample.

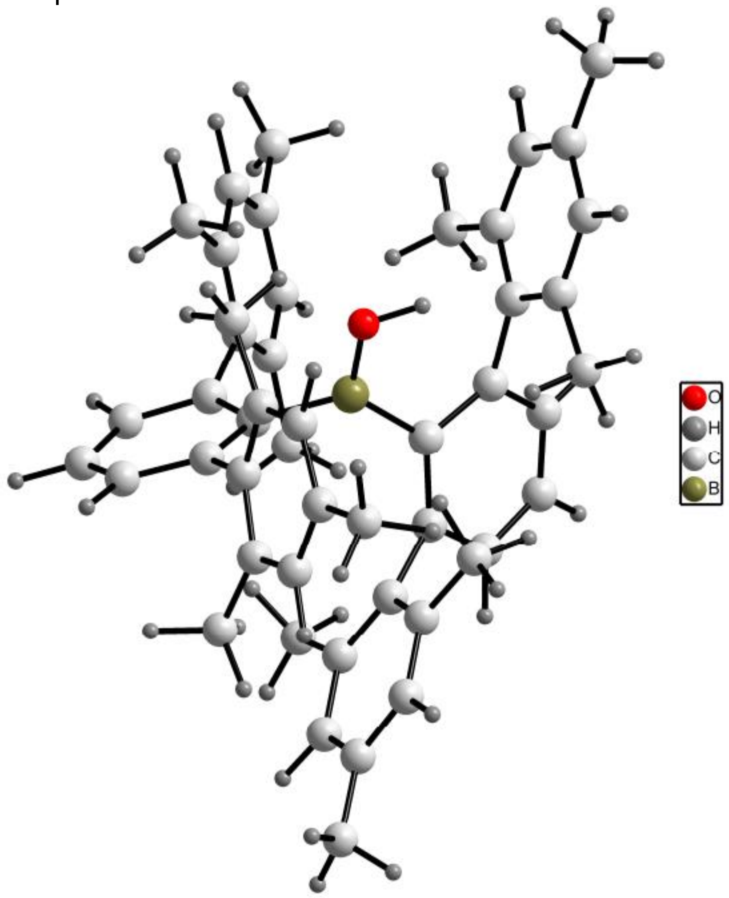

Figure S67. Asymmetric unit of **2** (polymorph a).

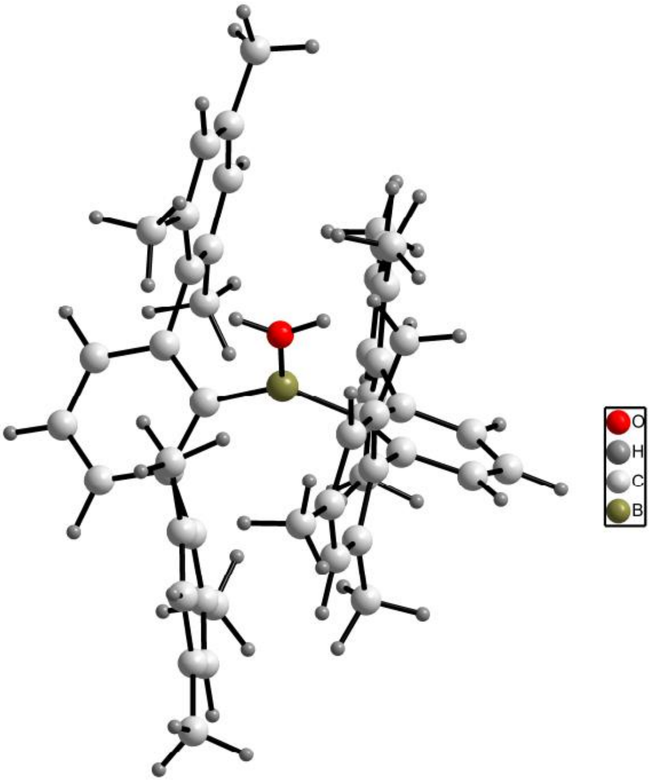

Figure S68. Asymmetric unit of **2** (polymorph b); Note: 50% occupancy each across two positions for the O–H bound hydrogen atom.

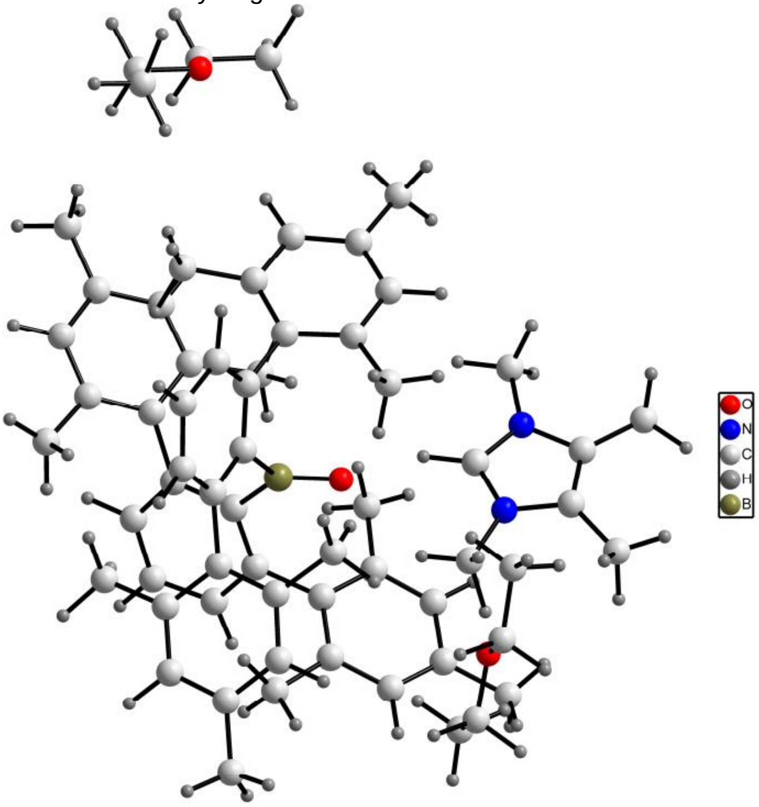

Figure S69. Asymmetric unit of **3a**.

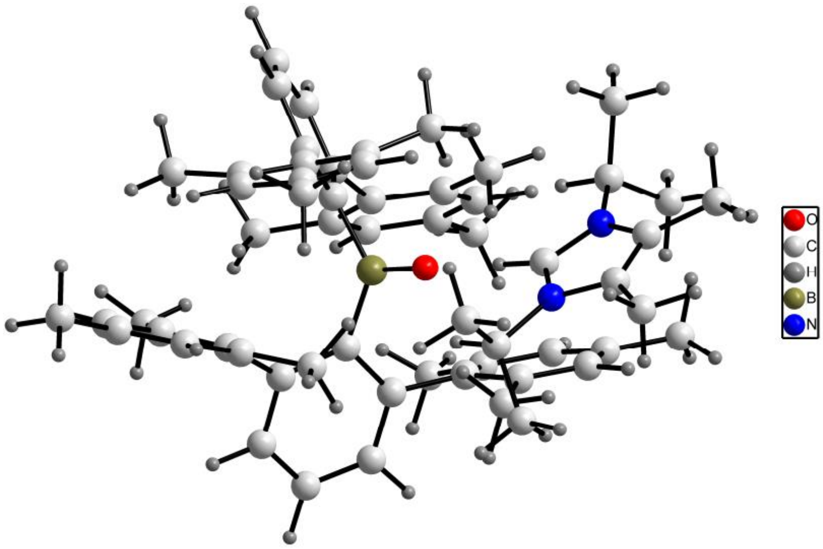

Figure S70. Asymmetric unit of **3b**.

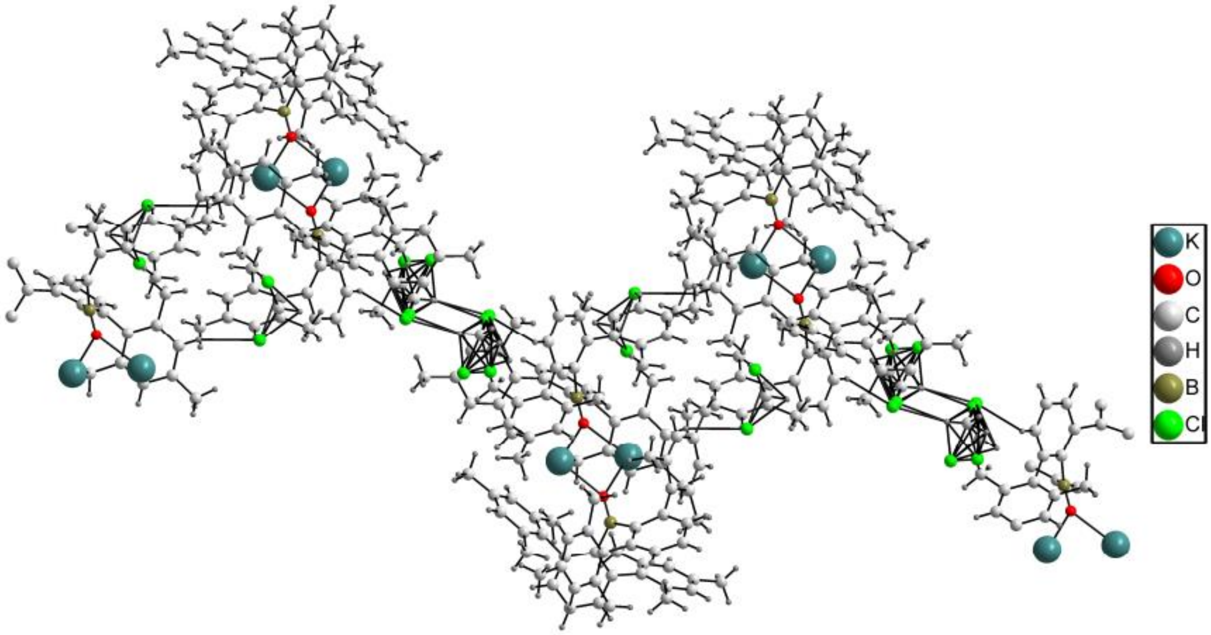

Figure S71. Asymmetric unit of **4**.

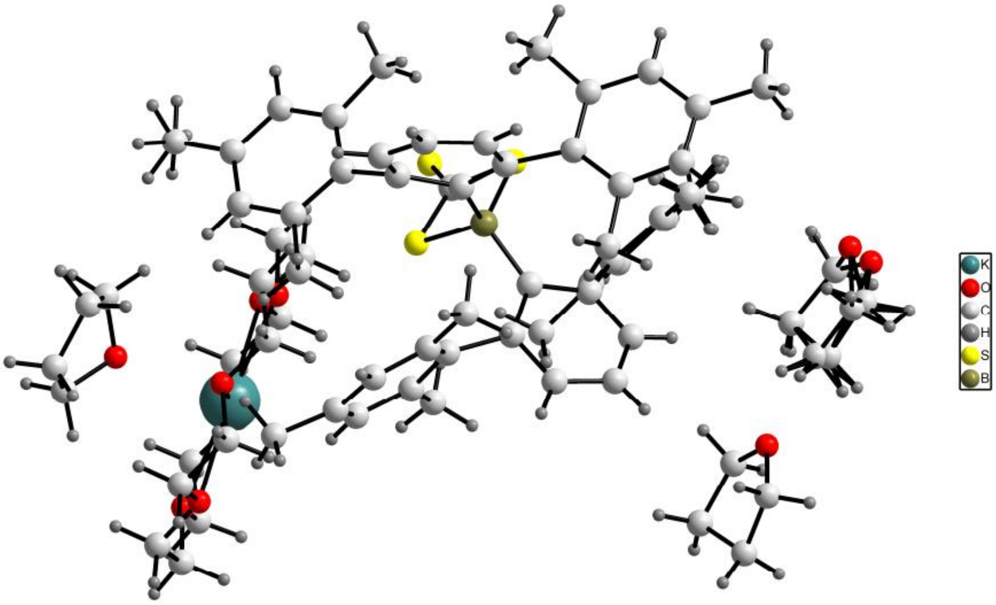

Figure S72. Asymmetric unit of **6a**.

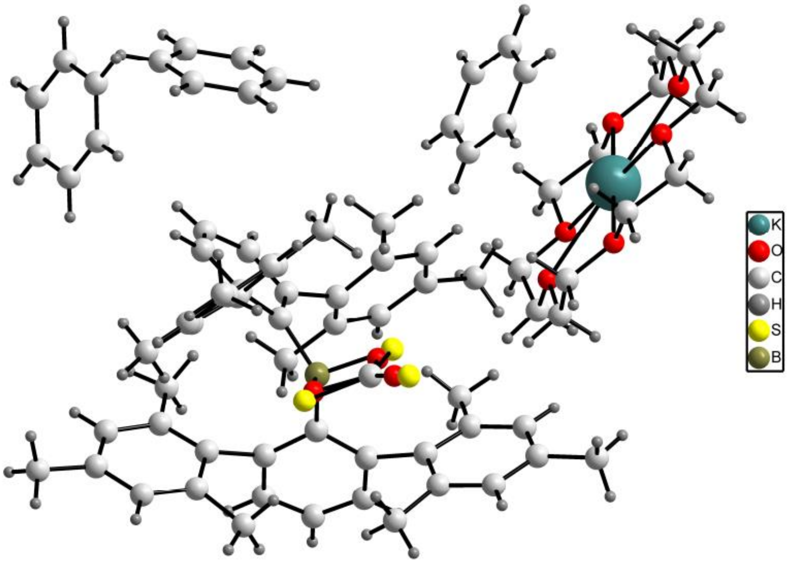

Figure S73. Asymmetric unit of **6a'**.

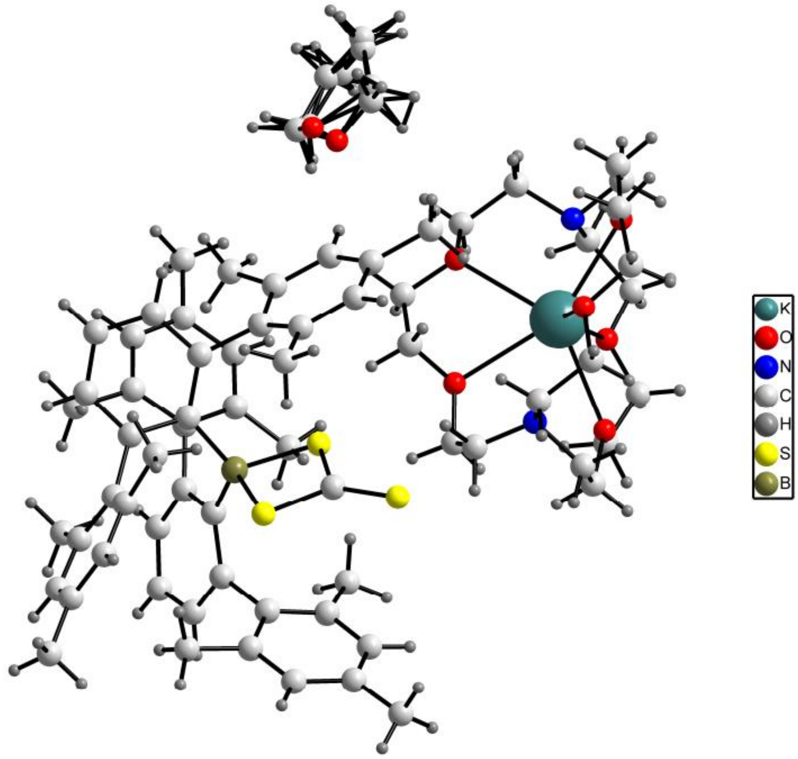

Figure S74. Asymmetric unit of **6b**.

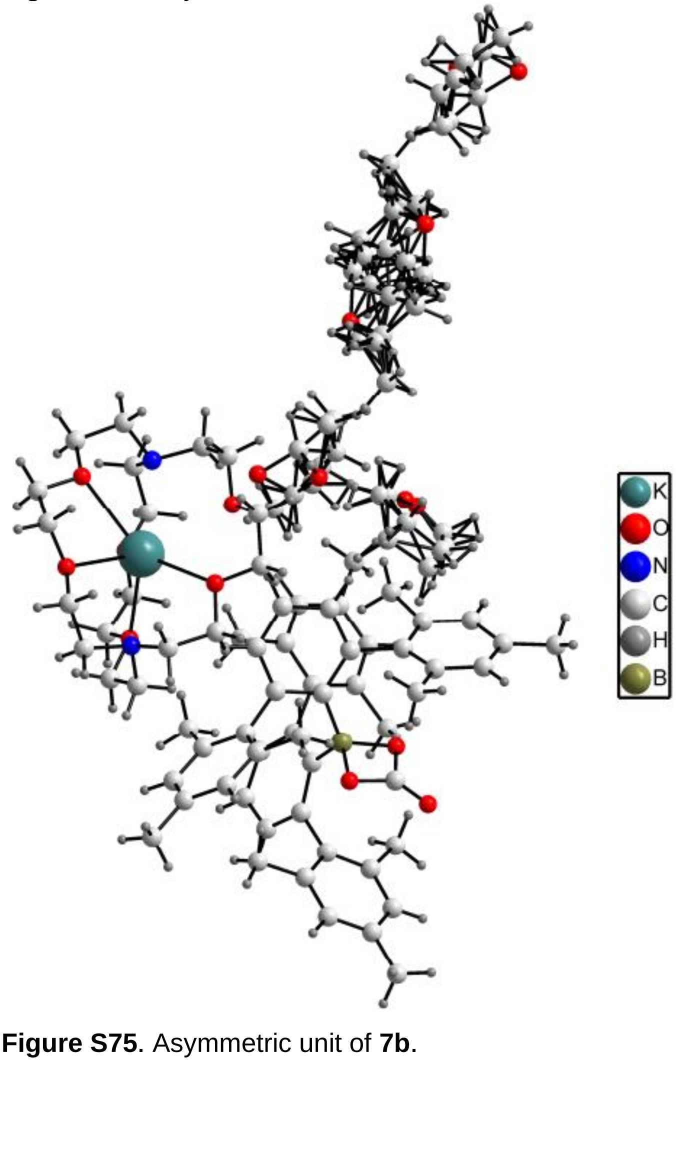

Figure S75. Asymmetric unit of **7b**.

Computational Details

Geometry optimizations were performed using density functional theory (DFT) with the B3PW91[S16,S17] functional and the 6-311+G(2df,p)[S18] basis set. Subsequent frequency analyses confirmed all obtained structures to be local minima on the potential energy surfaces. Dispersion effects were modelled using Grimme's GD3BJ.[S19] All geometry optimizations were performed with the Gaussian 16 software.[S20]

The wavefunction files from the geometry optimizations were used for a topological analysis of the electron density according to the Atoms in Molecules partitioning scheme[S21] using AIMAll,[S22] whereas DGRID[S23] was used to generate and analyze the Electron-Localizability-Indicator (ELI-D) related real-space bonding descriptors[S24] applying a grid step size of 0.05 a.u. NBO/NLMO[S25] analysis was performed using the NBO6 software.[S26] IQA[S27] analysis was performed with AIMAll. NBOs, AIM bond critical points and bond paths are displayed with Multiwfn_3.8[S28] and VMD.[S29]

Table S5. Selected computed and experimental bond lengths and angles of $(^{Mes}Ter)_2CO$, $[(^{Mes}Ter)_2BO]^{-}$, $(^{Mes}Ter)_2BF$ as well as of the phenyl substituted analogues.

Bond	$(^{Mes}Ter)_2CO$	$[(^{Mes}Ter)_2BO]^{-}$	$(^{Mes}Ter)_2BF$
C-C	1.4992		
C-B		1.6310	1.5739
C-O	1.2112		
B-O		1.2891	
B-F			1.3336
C-C-C	121.02		
C-B-C		124.99	130.98
C-C-O	119.49		
C-B-O		117.50	
C-B-F			114.51
	Ph_2CO	$[Ph_2BO]^{-}$	Ph_2BF
C-C	1.4901		
C-B		1.6212	1.5521
C-O	1.2158		
B-O		1.2874	
B-F			1.3403
C-C-C	119.20		
C-B-C		116.79	126.12
C-C-O	120.40		
C-B-O		121.61	
C-B-F			116.94

Table S6. Topological and integrated bond properties from AIM and ELI-D.

Species	d [Å]	ρ(r) [eÅ^{-3}]	∇^2ρ(r) [eÅ^{-5}]	ε	G/ρ(r) [a.u.]	H/ρ(r) [a.u.]	δ	N_{ELI} [e]	V_{ELI} [Å^3]
**$(^{Mes}Ter)_2CO$**									
**C-O**	1.211	2.84	–5.3	0.10	1.69	–1.82	1.35	2.32	3.7
**C-C**	1.499	1.79	–16.1	0.08	0.24	–0.87	0.95	2.26	4.1
**LP(O)**								2.62	6.2
**LP(O)**								2.62	6.2
**Ph_2CO**									
**C-O**	1.216	2.82	–7.0	0.08	1.64	–1.82	1.36	2.29	6.5
**C-C**	1.490	1.82	–16.6	0.09	0.24	–0.88	0.97	2.29	4.8
**LP(O)**								2.64	21.1
**LP(O)**								2.65	21.2
**$[(^{Mes}Ter)_2BO]^-$**									
**B-O**	1.289	1.76	27.2	0.03	2.13	–1.04	0.64	2.39	4.6
**C-B**	1.632	1.08	–4.1	0.11	0.75	–1.02	0.44	2.31	6.8
**LP(O)**								2.61	8.1
**LP(O)**								2.61	8.1
**$[Ph_2BO]^-$**									
**B-O**	1.287	1.77	27.5	0.03	2.14	–1.05	0.67	2.44	11.4
**C-B**	1.622	1.08	–3.3	0.08	0.80	–1.01	0.43	2.32	8.5
**LP(O)**								2.60	26.8
**LP(O)**								2.61	26.8
**$(^{Mes}Ter)_2BF$**									
**B-F**	1.334	1.35	28.5	0.18	2.25	–0.77		1.33	1.6
**C-B**	1.574	1.29	–8.9	0.09	0.63	–1.11		2.36	6.9
**LP(F)**								3.22	6.1
**LP(F)**								3.22	6.1
**Ph_2BF**									
**B-F**	1.340	1.32	27.6	0.17	2.23	–0.77	0.41	1.28	1.3
**C-B**	1.552	1.32	–7.8	0.04	0.71	–1.12	0.50	2.43	12.3
**LP(F)**								3.24	16.7
**LP(F)**								3.25	16.7

Table S7. Topological bond properties from AIM.

**Species**	$S_{C/B}$ **[%]**	$S_{O/F}$ **[%]**	E_{int} **[kcal mol^{-1}]**	E_C **[kcal mol^{-1}]**	E_{XC} **[kcal mol^{-1}]**
**CO**<br>**(Ph_2CO)**	39	54	–759.3	–503.5<br>(66%)	–255.9<br>(34%)
**BO**<br>**([Ph_2BO]$^-$)**	30	58	–1017.3	–898.4<br>(88%)	–119.0<br>(12%)
**BF**<br>**(Ph_2BF)**	27	54	–604.6	–529.7<br>(88%)	–74.9<br>(12%)

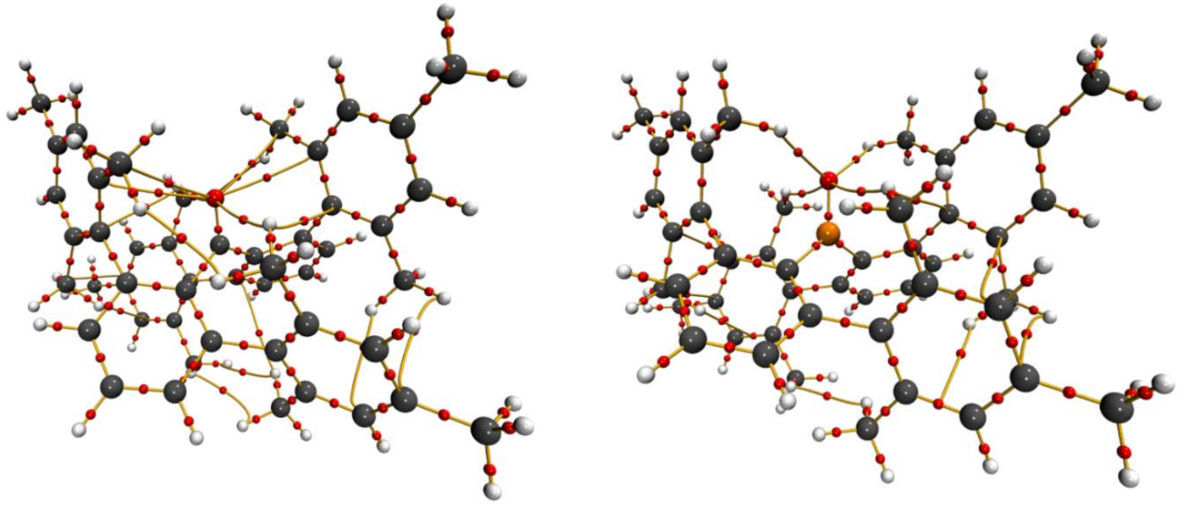

Figure S76. AIM molecular graphs of (^{Mes}Ter)$_2$CO (left), [(^{Mes}Ter)$_2$BO]$^-$ (right) with bond critical points as red spheres and bond paths in orange.

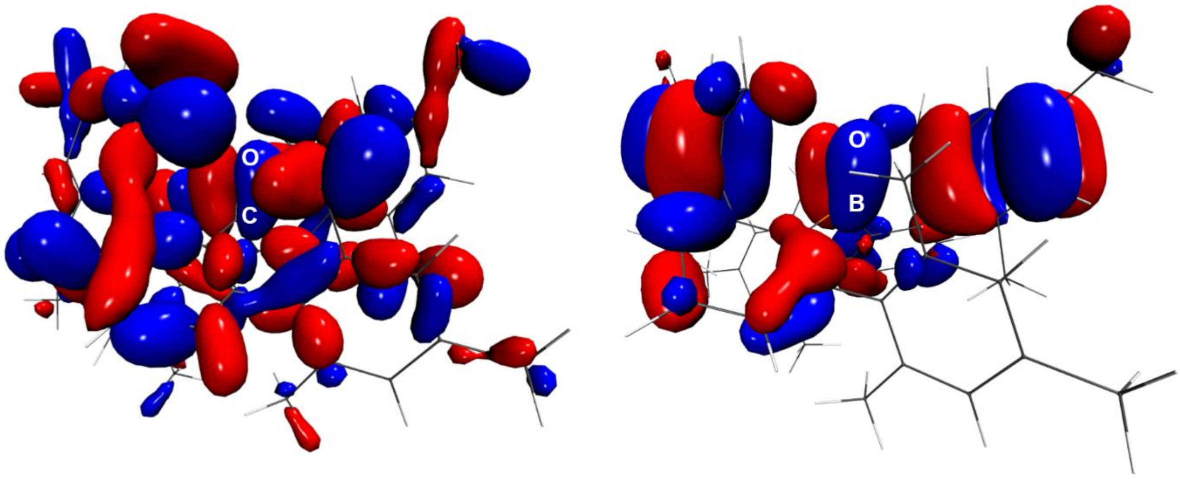

Figure S77. HOMO-25 of (^{Mes}Ter)$_2$CO (left) and HOMO-1 of [(^{Mes}Ter)$_2$BO]$^-$ (right) with *iso*-surface values of +0.02/-0.02 (blue/red).

Table S8. Wiberg Bond Indices (WBI), NLMO/NPA Bond Orders, delocalization index (DI) and atomic charges [in e].

Species	Contact	BO			Q(C/B)		Q(O/F)
		WBI	NLMO/NPA	DI	Bader	NPA	Bader
**$(^{Mes}Ter)_2CO$**	C–O	1.75	1.32	1.35	1.02	0.61	–1.14
**$(^{Mes}Ter)_2CO$**	C–C	0.99	0.97	0.95			
**$[(^{Mes}Ter)_2BO]^-$**	B–O	1.35	0.83	0.64	2.08	0.96	–1.49
**$[(^{Mes}Ter)_2BO]^-$**	C–B	0.77	0.56	0.44			
**$(^{Mes}Ter)_2BF$**	B–F	0.65	0.36		2.12	1.47	–0.86
**$(^{Mes}Ter)_2BF$**	C–B	0.73	0.53				

Table S9. NBO analysis of selected bonds of $(^{Mes}Ter)_2CO$, $[(^{Mes}Ter)_2BO]^-$, and $(^{Mes}Ter)_2BF$ with respective hybrids.

Model	Occupation	Atom 1	Hybrid	Atom 2	Hybrid
**$(^{Mes}Ter)_2CO$**					
**LP(1) O**	1.97	O			
**LP(2) O**	1.88	O			
**BD(1) O-C**	1.99	O (66.21%)	$sp^{1.33}d^{0.01}$	C (33.79%)	$sp^{2.31}$
**BD(2) O-C**	1.97	O (67.29%)	$p^{1.00}$	C (32.71%)	$p^{1.00}$
**$[(^{Mes}Ter)_2BO]^-$**					
**LP(1) O**	1.96	O			
**LP(2) O**	1.86	O			
**BD(1) O-B**	1.99	O (79.47%)	$sp^{0.73}$	B (20.53%)	$sp^{2.28}d^{0.01}$
**BD(2) O-B**	1.96	O (81.12%)	$p^{1.00}$	B (18.88%)	$p^{1.00}$
**$(^{Mes}Ter)_2BF$**					
**LP(1) F**	1.98				
**LP(2) F**	1.97				
**LP(3) F**	1.88				
**LP(4) F**	1.76				
		**Second Order Pertubation Theory**			
**Donor**	**Acceptor**	**E2**			
**NBO**	**NBO**	**[kcal/mol]**			
LP(1) F	LV(1) B	14.71			
LP(3) F	LV(2) B	35.41			
LP(4) F	LV(1) B	221.68			

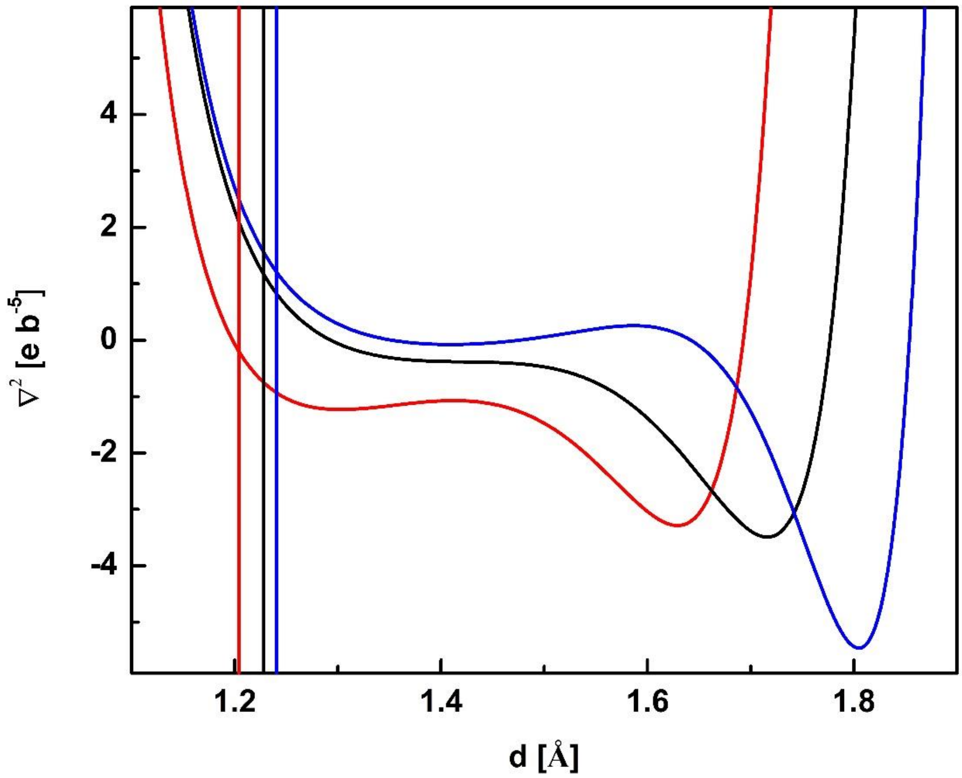

Figure S78. Laplacian of the electron density along the C–O, B–O, and B–F bond axes, respectively: red: (^{Mes}Ter)$_2$CO; black: [(^{Mes}Ter)$_2$BO]$^{–}$; blue: (^{Mes}Ter)$_2$BF. Position of each of the C and B atoms is at x = 1.5 bohr (0.79 Å). Vertical lines represent the position of the respective bond critical points.

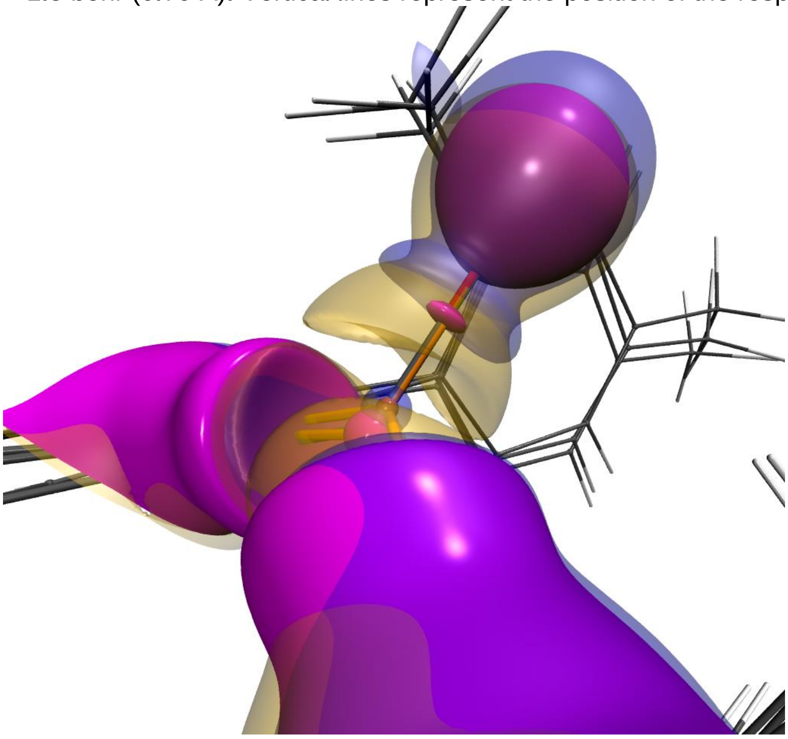

Figure S79. Laplacian of the electron density of (^{Mes}Ter)$_2$CO (transparent yellow), [(^{Mes}Ter)$_2$BO]$^{–}$ (transparent blue), and (^{Mes}Ter)$_2$BF (solid magenta) at iso-surface values of −0.03 e•bohr$^{−5}$.

References

[S1] Gupta, P.; Siewert, J.-E.; Wellnitz, T.; Fischer, M.; Baumann, W.; Beweries, T.; Hering-Junghans, C. Reactivity of phospha–Wittig reagents towards NHCs and NHOs. *Dalton Trans.* **2021**, *50*, 1838–1844.

[S2] Kuhn, N.; Kratz, T. Synthesis of Imidazol-2-ylidenes by Reduction of Imidazole-2(3*H*)-thiones. *Synthesis* **1993**, 561–562.

[S3] Duvinage, D.; Malaspina, L. A.; Grabowsky, S.; Mebs, S.; Beckmann, J. Lewis Superacidic Divalent Bis(*m*-terphenyl)element Cations $[(2,6\text{-}Mes_2C_6H_3)_2E]^+$ of Group 13 Revisited and Extended (E=B, Al, Ga, In, Tl). *Eur. J. Inorg Chem.* **2023**, *26*, e202200482.

[S4] Flörke, U.; Ayaz, M.; Henkel, G. CCDC 1521482: Experimental Crystal Structure Determination, **2016**, doi: 10.5517/ccdc.csd.cc1n2721.

[S5] Hanawalt, E. M.; Farkas, J.; Richey, H. G. Organomagnesates from Reactions of Dialkylmagnesium Compounds with Alkali-Metal Alkoxides, Potassium Hydride, and Other Salts. *Organometallics* **2004**, *23*, 416–422.

[S6] Mao, F.; Sui, D.; Qi, Z.; Fan, H.; Chen, R.; Huang, J. Heterogeneous cobalt catalysts for reductive amination with H_2: general synthesis of secondary and tertiary amines. *RSC Adv.* **2016**, *6*, 94068–94073.

[S7] Lucke, A. J.; Young, D. J. Electrophilic Cleavage of Cyclopropylmethystannanes: An Experimental Comparison of σ–σ and σ–π Conjugation. *J. Org. Chem.* **2005**, *70*, 3579–3583.

[S8] Bruker AXS Inc., in *Bruker Apex CCD, SAINT v8.40B* (Ed.: Bruker AXS Inst. Inc.), WI, USA, Madison, **2019**.

[S9] Krause, L.; Herbst-Irmer, R.; Stalke, D. Comparison of silver and molybdenum microfocus X-ray sources for single-crystal structure determination. *J. Appl. Crystallogr.* **2015**, *48*, 3–10.

[S10] Sheldrick, G. M. *SHELXT* – Integrated space-group and crystal-structure determination. *Acta Crystallogr.* **2015**, *A71*, 3–8.

[S11] Sheldrick, G. M. Crystal structure refinement with *SHELXL*. *Acta Crystallogr.* **2015**, *C71*, 3–8.

[S12] Dolomanov, O. V.; Bourhis, L. J.; Gildea, R. J.; Howard, J. A.; Puschmann, H. *OLEX2*: a complete structure solution, refinement and analysis program. *J. Appl. Crystallogr.* **2009**, *42*, 339–341.

[S13] Hübschle, C. B.; Sheldrick, G. M.; Dittrich, B. *ShelXle*: a Qt graphical user interface for *SHELXL*. *J. Appl. Crystallogr.* **2011**, *44*, 1281–1284.

[S14] Kleemiss, F.; Dolomanov, O. V.; Bodensteiner, M.; Peyerimhoff, N.; Midgley, L.; Bourhis, L. J.; Genoni, A.; Malaspina, L. A.; Jayatilaka, D.; Spences, J. L.; White, F.; Grundkötter-Stock, B.; Steinhauer, S.; Lentz, D.; Puschmann, H.; Grabowsky, S. Accurate crystal structures and chemical properties from NoSpherA2. *Chem. Sci.* **2021**, *12*, 1675–1692.

[S15] Neese, F. Software update: The ORCA program system—Version 5.0. *WIREs Comput. Mol. Sci.* **2022**, *12*, e1606.

[S16] Becke, A. D. Density functional thermochemistry. III. The role of exact exchange. *Chem. Phys.* **1993**, *98*, 5648–5652.

[S17] Perdew, J. P.; Chevary, J. A.; Vosko, S. H.; Jackson, K. A.; Pederson, M. R.; Singh, D. J.; Fiolhais, C. Atoms, molecules, solids, and surfaces: Applications of the generalized gradient approximation for exchange and correlation. *Phys. Rev. B* **1992**, *46*, 6671–6687.

[S18] (a) Krishnan, R.; Binkley, J. S.; Seeger, R.; Pople, J. A. Self-consistent molecular orbital methods. XX. A basis set for correlated wave functions. *J. Chem. Phys.* **1980**, *72*, 650–653; (b) McLean, A. D.; Chandler, G. S. Contracted Gaussian basis sets for molecular calculations. I. Second row atoms *Z*=11–18. *J. Chem. Phys.* **1980**, *72*, 5639–5648.

[S19] Grimme, S. Antony, J.; Ehrlich, S.; Krieg, H. A consistent and accurate *ab initio* parametrization of density functional dispersion correction (DFT-D) for the 94 elements H-Pu. *J. Chem. Phys.* **2010**, *132*, 154104.

[S20] Gaussian 16, Revision C.01, Frisch, M. J.; Trucks, G. W.; Schlegel, H. B.; Scuseria, G. E.; Robb, M. A.; Cheeseman, J. R.; Scalmani, G.; Barone, V.; Petersson, G. A.; Nakatsuji, H.; Li, X.; Caricato, M.; Marenich, A. V.; Bloino, J.; Janesko, B. G.; Gomperts, R.; Mennucci, B.; Hratchian, H. P.; Ortiz, J. V.; Izmaylov, A. F.; Sonnenberg, J. L.; Williams-Young, D.; Ding, F.; Lipparini, F.; Egidi, F.; Goings, J.; Peng, B.; Petrone, A.; Henderson, T.; Ranasinghe, D.; Zakrzewski, V. G.; Gao, J.; Rega, N.; Zheng, G.; Liang, W.; Hada, M.; Ehara, M.; Toyota, K.; Fukuda, R.; Hasegawa, J.; Ishida, M.; Nakajima, T.; Honda, Y.; Kitao, O.; Nakai, H.; Vreven, T.; Throssell K; Montgomery, Jr., J. A.; Peralta, J. E.; Ogliaro, F.; Bearpark, M. J.; Heyd, J. J.; Brothers, E. N.; Kudin, K. N.; Staroverov, V. N.; Keith, T. A.; Kobayashi, R.; Normand, J.; Raghavachari, K.; Rendell, A. P.; Burant, J. C.; Iyengar, S. S.; Tomasi, J.; Cossi, M.; Millam, J. M.; Klene, M.; Adamo, C.; Cammi, R.; Ochterski, J. W.; Martin, R. L.; Morokuma, K.; Farkas, O.; Foresman J. B.; Fox, D. J. Gaussian Inc., Wallingford CT, ***2019***.

[S21] Bader, R. W. F. Atoms in Molecules. A Quantum Theory; Cambridge University Press: Oxford, U.K., **1991**.

[S22] AIMAll (Version 15.09.27), Keith, T. A. TK Gristmill Software, Overland Park KS, USA, **2015**.

[S23] Kohout, M. DGrid, version 5.1, Dresden, **2019**.

[S24] (a) Kohout, M. A measure of electron localizability. *Int. J. Quantum Chem.* **2004**, *97*, 651–658; (b) Kohout, M.; Wagner, F. R.; Grin, Y. Electron localizability indicator for correlated wavefunctions. III: singlet and triplet pairs. *Theor. Chem. Acc.* **2008**, *119*, 413–420.

[S25] Glendening, E. D.; Landis, C. R.; Weinhold, F. Natural bond orbital methods. *WIREs Comput. Mol. Sci.* **2012**, *2*, 1–42.

[S26] NBO 6.0., Glendening, E. D.; Badenhoop, J. K.; Reed, A. E.; Carpenter, J. E.; Bohmann, J. A.; Morales, C. M.; Landis, C. R.; Weinhold, F. Theoretical Chemistry Institute, University of Wisconsin, Madison, **2013**.

[S27] (a) Blanco, M. A.; Pendás, A. M.; Francisco, E. Interacting Quantum Atoms: A Correlated Energy Decomposition Scheme Based on the Quantum Theory of Atoms in Molecules. *J. Chem. Theory Comput.* **2005**, *1*, 1096–1109; (b) Francisco, E.; Pendás, A. M.; Blanco, M. A. A Molecular Energy Decomposition Scheme for Atoms in Molecules. *J. Chem. Theory Comput.* **2006**, *2*, 90–92.

[S28] Lu, T.; Chen, F. Mutliwfn : A multifunctional wavefunction analyzer. *J. Comput. Chem.* **2012**, *33*, 580–592.

[S29] Humphrey, W.; Dalke, A.; Schulten, K. VMD: visual molecular dynamics. *J. Mol. Graphics* **1996**, *14*, 33–38.
